# Single-cell RNA landscape of intratumoral heterogeneity and immunosuppressive microenvironment in advanced osteosarcoma

**DOI:** 10.1038/s41467-020-20059-6

**Published:** 2020-12-10

**Authors:** Yan Zhou, Dong Yang, Qingcheng Yang, Xiaobin Lv, Wentao Huang, Zhenhua Zhou, Yaling Wang, Zhichang Zhang, Ting Yuan, Xiaomin Ding, Lina Tang, Jianjun Zhang, Junyi Yin, Yujing Huang, Wenxi Yu, Yonggang Wang, Chenliang Zhou, Yang Su, Aina He, Yuanjue Sun, Zan Shen, Binzhi Qian, Wei Meng, Jia Fei, Yang Yao, Xinghua Pan, Peizhan Chen, Haiyan Hu

**Affiliations:** 1 Oncology Department of Shanghai Jiao Tong University Affiliated Sixth People’s Hospital, Shanghai, 200233 China; 2 Orthopaedic Department of Shanghai Jiao Tong University Affiliated Sixth People’s Hospital, Shanghai, 200233 China; 3 Central Laboratory of the First Hospital of Nanchang, Nanchang, 330008 China; 4 Pathology Department of Shanghai Jiao Tong University Affiliated Sixth People’s Hospital, Shanghai, 200233 China; 5 Department of Orthopedic Oncology, Changzheng Hospital of Naval Military Medical University, Shanghai, 200003 China; 6 MRC Centre for Reproductive Health & Edinburgh Cancer Research UK Centre, Queen’s Medical Research Institute, EH16 4TJ Edinburgh, United Kingdom; 7 Department of Biochemistry and Molecular Biology, School of Basic Medical Sciences, Southern Medical University, Guangzhou, 510515 China; 8 Guangdong Provincial Key Laboratory of Single Cell Technology and Application, Guangzhou, 510515 China; 9 Department of Biochemistry and Molecular Biology, Medical College of Jinan University, 601 Western Huangpu Avenue, Guangzhou, 510632 China; 10 Clinical Research Center, Ruijin Hospital, Shanghai Jiao Tong University School of Medicine, Shanghai, 201821 China

**Keywords:** Bone cancer, Cancer microenvironment, Tumour heterogeneity, Sarcoma

## Abstract

Osteosarcoma is the most frequent primary bone tumor with poor prognosis. Through RNA-sequencing of 100,987 individual cells from 7 primary, 2 recurrent, and 2 lung metastatic osteosarcoma lesions, 11 major cell clusters are identified based on unbiased clustering of gene expression profiles and canonical markers. The transcriptomic properties, regulators and dynamics of osteosarcoma malignant cells together with their tumor microenvironment particularly stromal and immune cells are characterized. The transdifferentiation of malignant osteoblastic cells from malignant chondroblastic cells is revealed by analyses of inferred copy-number variation and trajectory. A proinflammatory FABP4^+^ macrophages infiltration is noticed in lung metastatic osteosarcoma lesions. Lower osteoclasts infiltration is observed in chondroblastic, recurrent and lung metastatic osteosarcoma lesions compared to primary osteoblastic osteosarcoma lesions. Importantly, TIGIT blockade enhances the cytotoxicity effects of the primary CD3^+^ T cells with high proportion of TIGIT^+^ cells against osteosarcoma. These results present a single-cell atlas, explore intratumor heterogeneity, and provide potential therapeutic targets for osteosarcoma.

## Abstract (web summary)

Osteosarcomas are difficult to treat and have a limited response to immunotherapy. Here, the authors analyse osteosarcomas at the single-cell level, and identify both the transdifferentiation of malignant cells and an array of immune cells that could have implications for metastasis and immunotherapy.

## Introduction

Osteosarcoma (OS) is a highly aggressive malignant bone tumor frequently occurring in children and adolescents with an annual incidence of ~4.8 per million worldwide^1,2^. According to WHO classification of tumors of soft tissue and bone, a minimal quantity identification of neoplastic bone is sufficient to render a diagnosis of OS^3^. OS is thought to arise from the primitive mesenchymal-derived bone-forming cells, and it usually occurred in the bone that is growing quickly such as the bones near the ends of the leg or arm in children and young adults^4^. The standard treatment for OS consists of extensive surgical resection and chemotherapy, whereas radiation therapy is recommended for the unresectable tumor^5^. Although certain target-based agents such as vascular endothelial growth factor receptor-tyrosine kinase inhibitors (VEGFR-TKIs) have shown promising outcomes, the 5-year overall survival rate is only 66.2%^6^. The relapse and/or metastasis rate of OS remains higher than 30%. For those relapse and/or metastasis patients, the 5-year overall survival rate was <25%, due to resistance to chemotherapy or radiation treatments^7^. The intrinsic genetic heterogeneity and dynamic immunogenic features significantly affect the therapeutic outcomes^8–11^. For instance, immune checkpoint inhibitors led to a breakthrough in immunotherapy for a variety of solid tumor; however, the anti-PD-L1 treatment only has a limited therapeutic effect on OS^12–14^. Therefore, there is an urgent need to understand the molecular mechanisms underlying the OS development and progression, and to identify more efficient targets for therapeutic treatment.

The traditional transcriptomic investigation is based on mixture cellular populations, which lacks sufficient resolution in the identification of specific cellular types and is unable to determine the complexity of intratumoral heterogeneity in OS. Recently, single-cell RNA-sequencing (scRNA-seq) has shown promising values in exploring the intra-tumor heterogeneity of a variety of cancers^15,16^, and the cellular cross-talk with the tumor microenvironment (TME)^17,18^. In this study, we present a comprehensive analysis of the transcriptomic profiling of 100,987 qualified single cells from 8 primary osteoblastic and three primary chondroblastic OS lesions. Eleven major cell clusters are identified and the cellular properties of malignant cells and the major TME cells are characterized. We detect the intratumor heterogeneity properties of OS lesions and the transdifferentiation of malignant osteoblastic cells from malignant chondroblastic cells in chondroblastic OS lesions. Through TME single-cell deconvolution, we find the diversity of the myeloid cells, tumor-infiltrating lymphocytes (TILs), mesenchymal stem cells (MSCs) and fibroblasts. With trajectory analysis of osteoclast (OC) maturation, we reveal that the antigen presentation function becomes faded and the bone absorption activities increase. Importantly, the CD8^+^ T, CD4^+^ T, regulatory T (T-reg), and NKT cells in OS lesions highly express immunoreceptor inhibitory checkpoint marker TIGIT (T cell Immunoreceptor with Ig and ITIM domains), and blocking TIGIT in vitro significantly enhance the cytotoxicity effects of CD3^+^ T cells against OS cells. The current study provides a deeper insight into the cellular and molecular characteristics of OS and its TME properties, which may helpful for therapeutic methods development in future.

## Results

### Cellular constitution of OS tumor lesions

**Fig. 1:**
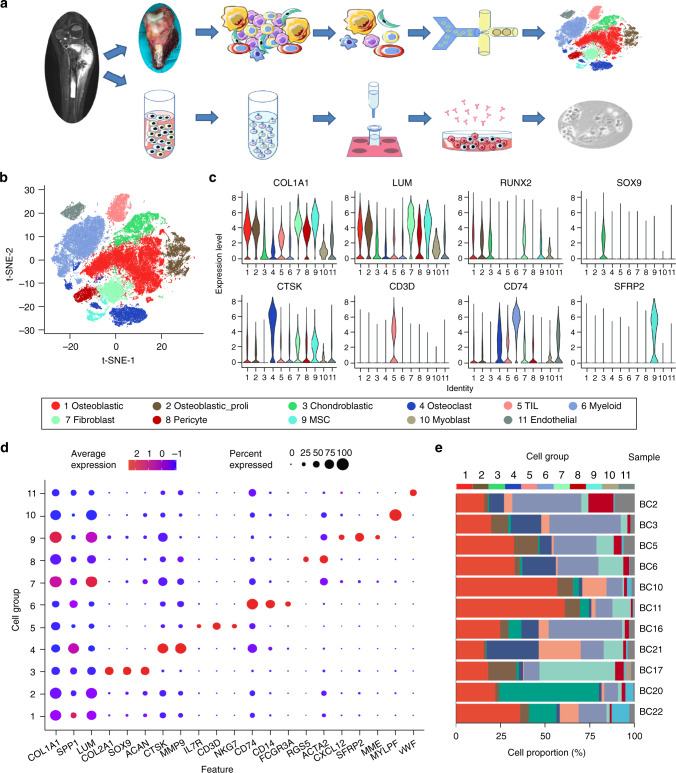
Single-cell transcriptomic analysis of OS lesions. **a** Graphical view of the study roadmap. Single-cell suspensions were collected from OS lesions of 11 patients followed by scRNA-seq on 10× Genomics platform, respectively. A total of 100,987 qualified single cells were recovered. The peripheral blood CD3^+^ T cells were isolated for cytotoxicity analysis for TIGIT blocking experiments. **b** The t-distributed stochastic neighbor embedding (t-SNE) plot of the 11 identified main cell types in OS lesions. **c** Violin plots showing the normalized expression levels of eight representative canonical marker genes across the 11 clusters. **d** Dot plots showing the 21 signature gene expressions across the 11 cellular clusters. The size of dots represents the proportion of cells expressing the particular marker, and the spectrum of color indicates the mean expression levels of the markers (log1p transformed). **e** Relative proportion of each cell cluster across 11 OS lesions as indicated. The values of the detailed relative proportion of each cell cluster are provided in the Source Data file.

We performed scRNA-seq analysis on tumor samples from 11 OS patients (five male and six female, 11–38-years old) to explore their cellular composition (Fig. 1a). Among them, eight lesions were osteoblastic OS, including six primary, one recurrent, and one lung metastatic lesions; three were chondroblastic OS with each was derived from primary, recurrent and lung metastasis site (Supplementary Table 1). After initial quality control assessment and doublet removal, we obtained single-cell transcriptomes from a total of 100,987 cells, including 65,895 cells from primary OS lesions, 17,735 cells from lung metastatic OS lesions, and 17,357 cells from recurrent OS lesions. The number of detected UMIs (unique molecular indexes) ranged from 1459 to 18,333 per cell with the mean of detected genes ranging from 704 to 4543 (Supplementary Fig. 1a, b).

Unbiased clustering of the cells identified 11 main clusters in parallel, based on t-distributed stochastic neighbor embedding (t-SNE) and uniform manifold approximation and projection (UMAP) analyses according to their gene profiles and canonical markers (Fig. 1b; Supplementary Table 2). In particular, they were as follows: (1) the osteoblastic OS cells highly expressing *COL1A1*, *CDH11*,and *RUNX2*; (2) the proliferating osteoblastic OS cells highly expressing osteoblastic cell markers and cell proliferating markers *TOP2, PCNA*, and *MKI67*; (3) the chondroblastic OS cells characterized with high *ACAN, COL2A1*, and *SOX9* expression; (4) the osteoclastic cells specifically express the markers *CTSK* and *MMP9*; (5) the TILs including T and NK cells with high expression of *IL7R, CD3D*, and *NKG7*; (6) the myeloid cells specifically expressing *CD74, CD14*, and *FCGR3A*; (7) the fibroblasts expressing *COL1A1, LUM*, and *DCN*; (8) the pericytes highly expressing α-smooth muscle actin *(α-SMA*, also known as *ACTA2*) and *RGS5*; (9) the MSCs expressing *CXCL12, SFRP*2, and *MME* (*CD10*); (10) the myoblasts specifically expressing *MYLPF* and *MYL1*; and (11) the endothelial cells specifically expressing *PECAM1* and *VWF*. The expression profiles of the representative genes in the cell populations were demonstrated (Fig. 1c, Supplementary Fig. 2). The top 20 differentially expressed genes (DEGs) for the subclusters of seven major clusters out of the 11 were also given (Supplementary Data 1–7), with more details extended in the following sections.

The dot plots compare the proportion of cells expressing cluster-specific markers and their scaled relative expression levels (Fig. 1d). We noticed that almost all types of cell populations were present in each individual lesion (Supplementary Figs. 3, 4) except for the myoblast, which were predominantly identified in the lung metastasis lesion BC17 (Fig. 1e, Supplementary Figs. 3, 4). The proportion of the cellular clusters varied significantly among the lesions (Fig. 1e, Supplementary Fig. 5a), suggesting the intertumoral heterogeneity as well as the consistency among the lesions. Regarding myoblast, of which the cell number was relatively small and the present rate is low, with 108 cells were annotated as the myoblast in BC17 (3.24%), two in BC5 and two in BC22, and not detected in other OS samples (Fig. 1e, Supplementary Figs. 3, 4). We suggested that the rarely detected myoblasts may due to heterogeneity of the OS tissues, under-sampling, but less likely contamination in sampling. The correlations of average gene expression profiles among the cellular clusters were also provided (Supplementary Fig. 5b).

### Transcriptional heterogeneity of malignant OS cells

**Fig. 2:**
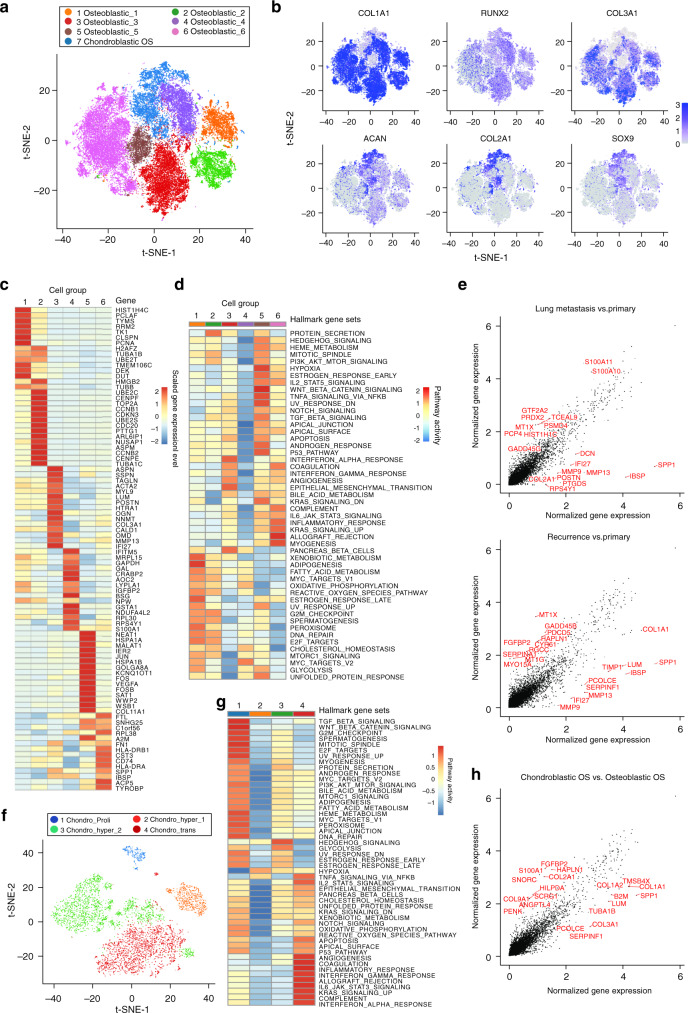
Distinct clusters of malignant cells in OS lesions. **a** Seven main malignant OS cell subclusters were identified by t-SNE analysis. **b** Feature plots for marker genes of osteoblastic (*COL1A1, RUNX2,* and *COL3A1*) and chondroblastic (*ACAN, COL2A1* and *SOX9*) tumor cells. The color legend shows the log1p normalized expression levels of the genes. **c** The heatmap of the average expression of top 15 DEGs among six subclusters of osteoblastic tumor cells. The color legend indicates normalized gene expression levels among the subclusters. **d** The heatmap of GSEA of the 50 hallmark gene sets in MSigDB database among the six osteoblastic cell subclusters. **e** The scatter plot of the DEGs between osteoblastic tumor cells from lung metastasis (upper panel) or recurrent lesion (lower panel) versus primary lesion. The top 10 DEGs in each comparison were labeled in red. **f** The t-SNE plot of the four subclusters of chondroblastic cells. **g** The heatmap of GSEA of the 50 hallmark gene sets in MSigDB among the four chondroblastic clusters. **h** The scatter plot of the DEGs between chondroblastic and osteoblastic malignant cells. The top 10 genes in each subcluster were marked in red. Relative GSEA scores for each gene set across the cell clusters (**d**, **g**) and detailing average normalized gene expression (log1p transformed) values in different tumor sites (**e**) or types (**h**) are provided in the Source Data file.

Osteoblastic and chondroblastic OS are the two major types of conventional OS in a clinic. Through the t-SNE analysis of malignant OS cells, we identified seven subclusters in total, of which six were of osteoblastic lineage and 1 belonged to the chondroblastic lineage (Fig. 2a). The distribution of each cluster of single cells from three different types of lesions, i.e., primary, recurrence, and metastasis, was shown (Supplementary Fig. 6a). The gene expressing patterns in different clusters of the malignant osteoblastic and chondroblastic OS cells were presented (Fig. 2b). The chondroblastic lineage subclusters were characterized by relatively high expression of *SOX9*, *COL2A1*, and *ACAN* (Fig. 2b). The osteoblastic lineage displayed high levels of osteoblastic maturation markers including *COL1A1*, *COL3A1*, and *RUNX2* (Fig. 2b). The numbers and proportions of the malignant OS cells varied among the patients (Supplementary Fig. 6b, c). Interestingly, BC20 and BC22, the primary and recurrent chondroblastic OS samples, respectively, had both the chondroblastic and osteoblastic lineage malignant cells. Another OS sample, the lung metastatic lesion BC17, with the corresponding in situ primary chondroblastic OS, contained predominantly the osteoblastic OS cells rather than chondroblastic OS cells (59 chondroblastic OS cells and 1,103 osteoblastic OS cells), which might attribute to the fact that chondroblastic OS cells were less aggressive and thus, identified less in the metastatic lesions. Hematoxylin-eosin (H&E) staining of the primary chondroblastic OS and the lung metastatic lesion (BC17) was performed, which confirmed the scRNA-seq results of BC17 (Supplementary Fig. 6d).

Among the six subclusters of malignant osteoblastic cells, Osteoblastic_1 and Osteoblastic_2, corresponding to the original cluster of proliferating osteoblastic cells, showed high proliferation rate, co-expressing osteoblastic cell markers (including *COL1A1*, *RUNX2,* and *COL3A1,* etc.) and cell proliferation markers (such as *TOP2A, PCNA*, and *MKI67,* etc.). As demonstrated in the list of the top DEGs (Fig. 2c; Supplementary Data 1), specifically Osteoblastic_1 expressed high levels of mitotic S phase genes including *PCNA*, *TYMS*, and *RRM*2, while Osteoblastic_2 expressed high levels of G2/M phase genes such as *UBE2C* and *HMGB2*. The other four subclusters were the typical malignant osteoblastic cells. Based on competitive gene set enrichment analysis (GSEA) analysis (Fig. 2d), Osteoblastic_3 showed heightened activities of angiogenesis, IFN-α and IFN-γ signaling pathways; Osteoblastic_4 enriched with MYC and oxidative phosphorylation signaling pathways; Osteoblastic_5 enriched with TGF-β, P53, KRAS, and hypoxia signaling pathways; and Osteoblastic_6 enriched with myogenesis, inflammatory responses, and allograft rejection signaling pathways.

We managed to find the specific gene expression pattern for lung metastasis or recurrent osteoblastic OS cells compared with the primary osteoblastic OS cells (Fig. 2e). The analysis revealed that the top over-expressed genes in the lung metastatic lesions included *S100A11*, *S100A10*, *PRDX2*, and *PSMD4*, which were reported to be promoting the metastasis and/or tumorigenesis in multiple types of cancer^19–22^. The functional gene ontologies (GOs) enriched in lung metastatic OS were oxidative phosphorylation pathway, *MYC* gene targets, reactive oxygen species pathway, mTORC1 and hypoxia signaling pathways (Supplementary Fig. 6e). In the recurrent OS lesions, the genes *MT1X*, *GADD45B*, *HAPLN1*, and *CYR61* were significantly enriched (Fig. 2e). Accordingly, hypoxia, *TNF-α*, *TGF-β*, *IL2-STAT5*, and the mTORC1 pathways were enriched in recurrent tumor cells (Supplementary Fig. 6f), suggesting that these pathways may be critical for the OS chemotherapeutic resistance and tumor recurrence.

Further t-SNE clustering of the malignant chondroblastic cells identified four subclusters with distinct gene expression patterns (Fig. 2f and Supplementary Data 2). The numbers and proportions malignant chondroblastic OS cells among the patients and tissue sites were demonstrated (Supplementary Fig. 6g, h, i). In particular, the proliferating malignant chondroblastic cells (Chondro_Proli) expressed *TOP2A, PCNA, TYMS* and *MKI67*. In addition, the two subclusters of hypertrophic chondroblastic cells exhibited relatively high expression of *MEF2C*, *PTH1R*, and *IHH* (Chondro_hyper_1 and Chondro_hyper_2). The last subcluster, Chondro_trans cells were under trans-differentiation into osteoblastic cells with high *RUNX2*, *SPP1*, and *COL1A1* levels, and relatively low levels of *COL2A1* and *SOX9*, suggesting that the malignant osteoblastic cells were possibly derived from the chondroblastic cells (Supplementary Fig. 7; Supplementary Data 2). The GSEA analysis for chondroblastic cells (Fig. 2g), revealed relatively high gene expression associated with IL-2/STAT5, Hedgehog, and Notch pathway in the subcluster Chondro_hyper_1, while the subcluster Chondro_hyper_2 exhibited higher inflammation responses and IL-6/JAK/STAT3 pathways (Fig. 2g). The top 10 DEGs of malignant chondroblastic and the osteoblastic cells were identified (Fig. 2h). The GO enrichment analysis suggested that the chondroblastic cells were enriched with genes related to chondrocyte differentiation, collagen fibril organization, and cartilage development etc. compared to the osteoblastic cells (Supplementary Fig. 6j).

### Clonality analysis of malignant OS cells

**Fig. 3:**
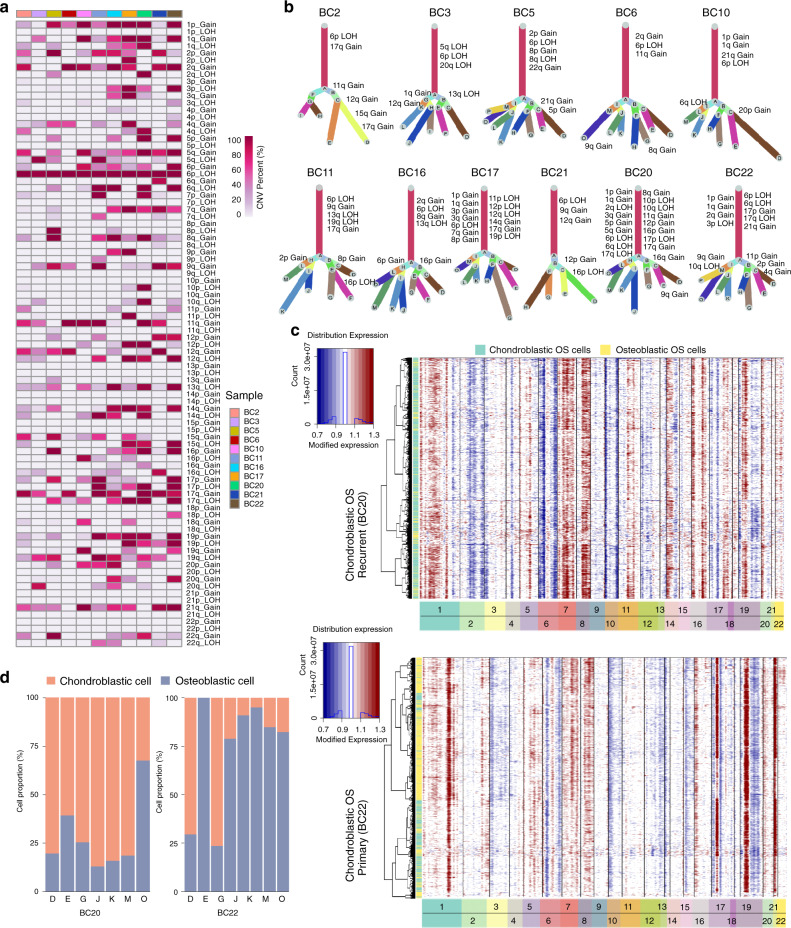
Copy-number variation and clonal evolution analysis of OS cells. **a** The summary CNV profiles of the OS cells for the 11 OS samples inferred from inferCNV analysis. The CNV levels were categorized by the chromosome arm and simplified as gain or loss in single cells. Color in the heatmap indicated the percent of the CNV events in the single cells from each individual sample. **b** Clonality trees of the single cells from each patient. The branches are delineated according to the percentage of cells in the subclone containing the corresponding CNVs. The canonical CNV events in each lesion were labeled in the clonality tree. **c** The hierarchical heatmap showing large-scale CNVs in chondroblastic OS lesions form one primary (BC22) and one recurrent (BC20) OS sample (see Supplementary Fig. 8 for the other nine OS samples). **d** The percentage of chondroblastic and osteoblastic OS cells in each branch of clonality tree as indicated in (**b**) for the two lesions (BC20 and BC22). The percent value of the chromosomal CNV events (**a**) in the single cells from each individual sample is provided in the Source Data file.

OS is characterized by significant somatic copy-number alteration (SCNA) and structural variation (SV) with few recurrent point mutations in protein-coding genes^23^. To probe the clonal structure of OS cells, we applied inferCNV algorithm to analyze the copy number variations (CNVs) of the single cells from each lesion (Fig. 3a, Supplementary Fig. 8)^24,25^. The 1p gain, 1q gain, 2q gain, 17q gain and 21q gain were the mostly noticed canonically chromosomal variations among the lesions, which were consistent with previously reported genomic CNVs observed in the comparative genomic hybridization (CGH) and whole-genome sequencing (WGS) studies^23,26,27^. Based on the aggregated CNV results, the clonality tree for OS lesion was generated using the UPhyloplot2 plotting algorithm^28^. Multiple canonical (CNV percent > 90%) and non-canonical CNVs (CNV percent < 90%) in subclones were noticed in each of the lesions (Fig. 3b). The clonality analysis results revealed the previously unappreciated complexity of both canonical and non-canonical CNVs in OS (Fig. 3a, b). As expected, the canonical CNVs dominated the chromosomal landscape. Nevertheless, there are still multiple subclonal canonical and non-canonical CNVs across OS patients, which underlie the subclonal cellular populations in tumor cells evolution (Fig. 3a, b).

Interestingly, more canonical CNVs were noticed in the primary chondroblastic OS lesion BC22 and recurrent chondroblastic OS lesion BC20 than osteoblastic OS samples (Fig. 3a, b); however further studies are required to confirm the CNVs. Previous studies suggested that normal chondroblastic cells could be trans-differentiated into osteoblastic cells during epiphyseal formation and fracture healing^29,30^. Based on the clonal tree, the mutual chromosomal alternation patterns were noticed in the branches of both lesions BC20 and BC22 (Fig. 3b–d), implying that the malignant osteoblastic cells might be derived from the malignant chondroblastic cells during the chondroblastic OS development and progression.

We further analyzed the tumor trajectory of the chondroblastic and osteoblastic OS cells in BC20 and BC22 using the Monocle 2 algorithm and slingshot algorithm, which are popular tools for the bifurcation trajectory analysis^31,32^. The bifurcation trajectory was noticed in both samples using these two algorithms (Supplementary Figs. 9a–d, 10a, b). The BC20 trajectory suggested that a branch of the chondroblastic cells could be transdifferentiated into the osteoblastic cells (cell fate 1; Supplementary Figs. 9a, 10a), during which the genes involved in the osteoblast differentiation, ossification, collagen organization, histone methylation, histone H3 K4 trimethylation and histone H4 acetylation were significantly increased (Supplementary Fig. 9e) and genes related to skeletal system morphogenesis and stem cell differentiation were reduced (Supplementary Figs. 9e, 10c). The other branch of the cells showed endochondral bone formation (cell fate 2) with the increased expression of genes associated with p53 mediated apoptosis and a reduction of the genes related to bone morphogenesis and stem cell differentiation (Supplementary Figs. 9e, 10c). For BC22, we noticed that two branches of osteoblastic tumor cells could be derived from the malignant chondroblastic cells (cell fate 1 and 2; Supplementary Figs. 9c, 10b) through activating distinct gene signaling pathways involved in the histone deacetylation, bone morphogenesis, and osteoblast differentiation processes (Supplementary Figs. 9f, 10d). Consistent with the analysis results of CNV patterns, these results again suggested that the malignant chondroblastic cells could be transdifferentiated into the malignant osteoblastic cells in OS TME.

### Trajectory of OC maturation in OS lesions

**Fig. 4:**
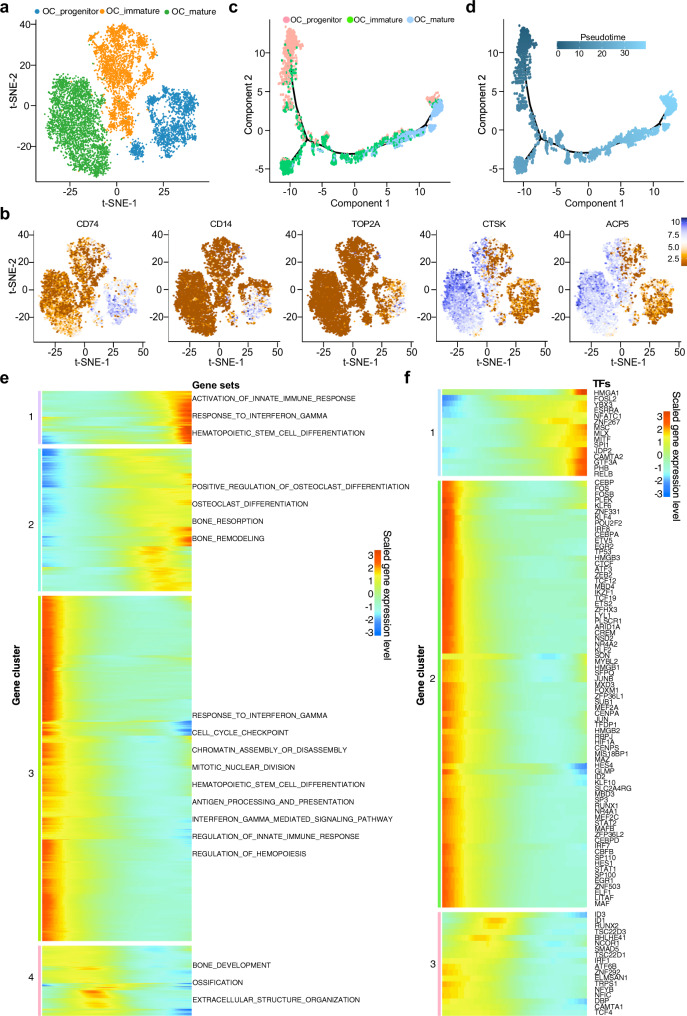
Trajectory analysis of osteoclast cells (OC) in OS lesions. **a** t-SNE plot showing the three main subclusters of osteoclasts. **b** Feature plots showing the normalized expression levels of myeloid and osteoclast markers *CD74, CD14, ACP5, CTSK*, and *TOP2A* in these subclusters. **c**, **d** The Monocle 2 trajectory plot showing the dynamics of osteoclast subclusters (**c**) and their pseudotime curve (**d**). **e** The DEGs (in rows, *q*-value < 10^−10^) along the pseudotime were hierarchically clustered into four subclusters. The top annotated GO terms in each cluster were provided. **f** Heatmap hierarchical clustering showing differentially expressed transcription factor genes along with the pseudotime curve in (**e**).

OC, a set of relatively large multinucleated, specialized monocyte-macrophage lineage, plays a vital role in the osteolysis and tumor growth supporting in OS tissues^33^. Based on t-SNE and UMAP algorithm (Fig. 4a, Supplementary Fig. 11a, b), three distinct subclusters of OCs were identified with a distinct expression of both myeloid markers such as *CD74, CD14,* and/or mature osteoclastic markers including *CTSK* and *ACP5* (Fig. 4b; Supplementary Data 3). These subclusters were described as: (1) OC_progenitor cells expressed high levels of myeloid markers *CD74* and *CD14* with dim OC markers *CTSK* and *ACP5*. This subcluster cells also showed hyperproliferative phenotype with significantly high levels of *TOP2A*, alluding that they were under the stimulation of osteogenesis; (2) OC_immature cells co-expressed both of the myeloid and OC markers; and (3) OC_mature cells demonstrated high levels of *CTSK* and *ACP5* and low *CD74* and *CD14*. The cell numbers and proportion of these three subclusters of OC cells varied dramatically among different lesions (Supplementary Fig. 11c, d). We also detected OC in lung metastatic and recurrent lesion, which hinted that the function of OC as a key component in TME of an advanced OS; however, the proportion of mature OC cells was relatively lower in the chondroblastic, lung metastatic, and recurrent lesions compared to the primary OS lesions. This phenomenon indicated that the OC status may depend on the chemotactic signal strength of osteogenesis in OS lesions^34–36^.

We also performed the trajectory analysis of the OCs based on the Monocle 2 algorithm and SCORPIUS algorithm to infer OC maturation course in OS lesions (Fig. 4c, d, Supplementary Fig. 12a). Both trajectory algorithms showed that gene expression signature patterns were in concordance with the distribution of the three subclusters identified by the t-SNE plots (Supplementary Figs. 11e, f, 12b). Particularly, the gene patterns involved in the OC cell state transition were dissected (Fig. 4e, Supplementary Figs. 11e, 12b). The genes related to antigen processing and presentation, IFN-γ response, hematopoietic stem cell differentiation, mitotic nuclear division, and cell cycle checkpoint were significantly reduced, whereas the genes related to OC differentiation, bone resorption and bone remodeling were significantly increased (Fig. 4e, Supplementary Fig. 12b). Meanwhile, the transcriptional factors related to immune cell proliferation and differentiation, such as *HMGB1*, *HMGB2*, *MEF2C, ID1, ID3, CREM,* and *LITAF,* etc. (Fig. 4f), were gradually down-regulated along with trajectory differentiation process. Conversely, some well-known factors such as *NFATC1, SPI1*, and *FOSL2* were upregulated in the process (Fig. 4f), which are involved in regulating differentiation, survival and size of OC^37,38^. We also found some unidentified regulators such as *JDP2, ZNF267, CAMTA2, MLX, HES4,* and *GLMP* in OS lesions, which are potentially engaged in the cellular transition from the myeloid monocytes into mature OC cells (Fig. 4f).

With the immunohistochemical staining method, we confirmed that the cells highly positive for *CD74* (myeloid cells) were small and mononuclear, while the *CD74* levels were markedly reduced in multinuclear OCs in OS lesions (Supplementary Fig. 13a). Aiming to validate our trajectory observations, we detected co-expression of *CD74* and *CTSK* in OS samples by immunofluorescence staining (Supplementary Fig. 13b). The co-expressing of CD74 and CTSK in the same cells further underscored the transitional status of the myeloid cells into OC cells.

### Diversity of stromal MSCs and cancer-associated fibroblasts (CAFs)

MSCs in the TME had been proved to stimulate the tumor cellular proliferation, metastasis and drug resistance in various types of cancer including OS^39^. It is well known that MSCs are the multipotent stem cells that can differentiate into the osteoblasts, chondrocytes, and adipocytes under specific microenvironmental contexts^40^. Previous study suggested that the MSCs in the OS microenvironment still hold the multipotent activities^41^. In the OS lesions, we characterized MSCs by the proposed markers including *MME (CD10)*, *THY1 (CD90)*, and *CXCL12* (Fig. 1, Supplementary Fig. 2).

**Fig. 5:**
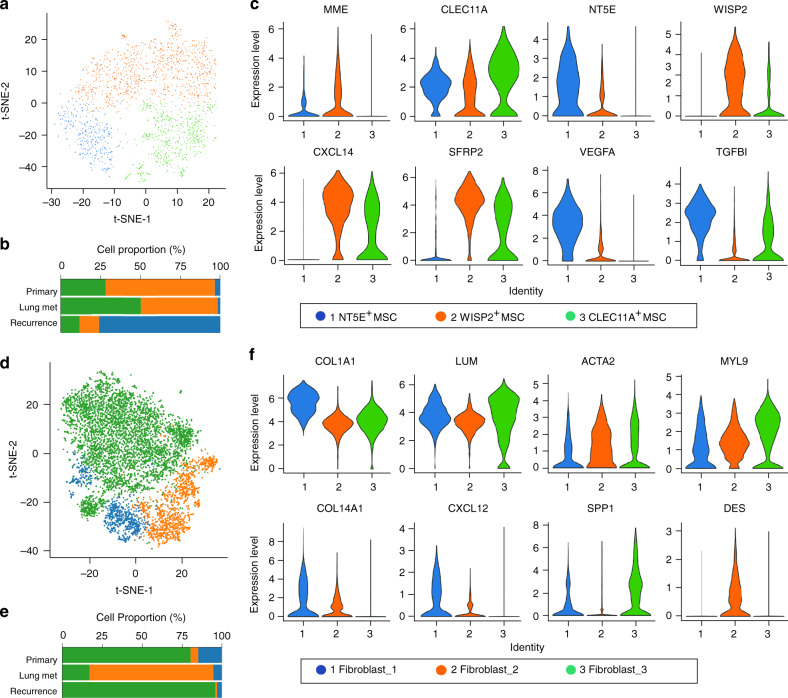
Clustering and identification of mesenchymal stem cells (MSCs) and cancer-associated fibroblasts (CAFs) subclusters in OS lesions. **a** t-SNE plot of MSCs identified in the 11 OS lesions, colored by the three subclusters of cells as indicated. **b** The mean percent of the 3 MSC subclusters in primary, lung metastasis and recurrent samples. **c** Violin plots showing the normalized expression levels of marker genes across the clusters. **d** t-SNE plot of three subclusters of CAFs identified in the 11 OS lesions. **e** The mean percent of CAF subclusters in the three types of lesions. **f** Violin plots showing the normalized expression levels of marker genes across the three subclusters of CAFs. The values of mean proportions of MSCs (**b**) and CAFs (**e**) subclusters are provided in the Source Data file.

With the t-SNE method, three cellular subclusters of MSCs were identified (Fig. 5a), which were termed as the NT5E^+^ MSCs, WISP2^+^ MSCs and CLEC11A^+^ MSCs based on the gene expression feature, representing MSC subcluster 1, 2 and 3, respectively. The proportion of each subcluster varied in the studied lesions (Fig. 5b, Supplementary Fig. 14a, b). We analyzed the expression characteristics for a set of featured genes in each MSC subcluster (Fig. 5c, Supplementary Fig. 14c; Supplementary Data 4) including: (1) the subcluster 1, predominantly observed in the chondroblastic OS lesions BC 20 and BC22 (Supplementary Fig. 14a, b), exhibited relatively high expression of MSC marker *NT*5*E (CD73)* with genes *VEGFA* and *TGFBI* (Fig. 5c, Supplementary Fig. 14c). Therefore, the NT5E^+^ MSCs may stimulate the angiogenesis and metastasis of the OS cells; (2) subcluster 2 was characterized by relatively high expression of the *WISP2* together with *CXCL14* (Fig. 5c, Supplementary Fig. 14c). Previous study reported that the secreted protein encoded by *WISP2* from MSC promoted the proliferation of MSCs rather than inhibition of the adipogenic commitment and differentiation^42^, while *CXCL14* was suggested to promote the metastasis of the OS cells^43^; (3) the subcluster 3 predominantly presented in the osteoblastic OS lesions, and showed a high level of *CLEC11A* (Fig. 5c), accompanied by relatively high expression levels of osteoblastic differentiation markers *SPP1* and *IBSP* (Supplementary Fig. 14c). *CLEC11A*, also known as osteolectin for the protein, promotes the differentiation of mesenchymal progenitors into mature osteoblasts in vitro and plays an important role in the maintenance of adult skeleton age-related bone loss and fracture repair^44^.

CAFs are another important component of the TME, which could stimulate the tumor progression, growth and metastasis^45^. It was reported that the malignant OS cells directly induced the differentiation of mesenchymal stem cells (BMSCs) into CAFs^46^. In this study, the CAFs exhibited remarkably high levels of fibroblast markers decorin (*DCN*) and lumican (*LUM*) with reduced osteoblast and MSC markers particularly *PTH1R*, *MME,* and *CXCL12* (Fig. 1a, Supplementary Fig. 2). Based on the t-SNE analysis, the CAFs were categorized into three subclusters (Fig. 5d, e). The cell number and proportion of all three subclusters in each lesion were detailed (Supplementary Fig. 14d, e). The classification of 3 CAF subclusters allowed the subtle features to be identified, despite the high expression of general markers for fibroblast, *COL1A1*, and *LUM* in all three subclusters (Fig. 5f, Supplementary Data 5). The fibroblast_1 was characterized by *COL14A*1, suggesting that they were *COL14A1*^+^ matrix fibroblasts. The fibroblast_2 was characterized by *DES*, coupled with low level of *ACTA2* and *COL14A1*, indicating that they could be the smooth muscle-like cells. The fibroblast_3 expressed relatively high levels of *MYL9* and *LUM*, with positive *ACTA2* but no expression of *COL14A1* and *DES*. It is similar to myofibroblasts, but showed relatively high expression of osteoblast markers *IBSP* and *SPP1*, suggesting that the cluster 3 CAFs in the OS lesions may play an osteoblast-like function. Nevertheless, the fibroblast_1 and fibroblast_3 cells were the main CAFs in both primary and recurrent lesions, and the fibroblast_2 cells were the main component of CAFs in lung metastasis lesions (Fig. 5c). The specific gene expression profiling of each CAF subcluster was also determined (Supplementary Fig. 14f; Supplementary Data 5).

### Heterogeneity of tumor-associated macrophages (TAMs) and dendritic cells (DCs)

**Fig. 6:**
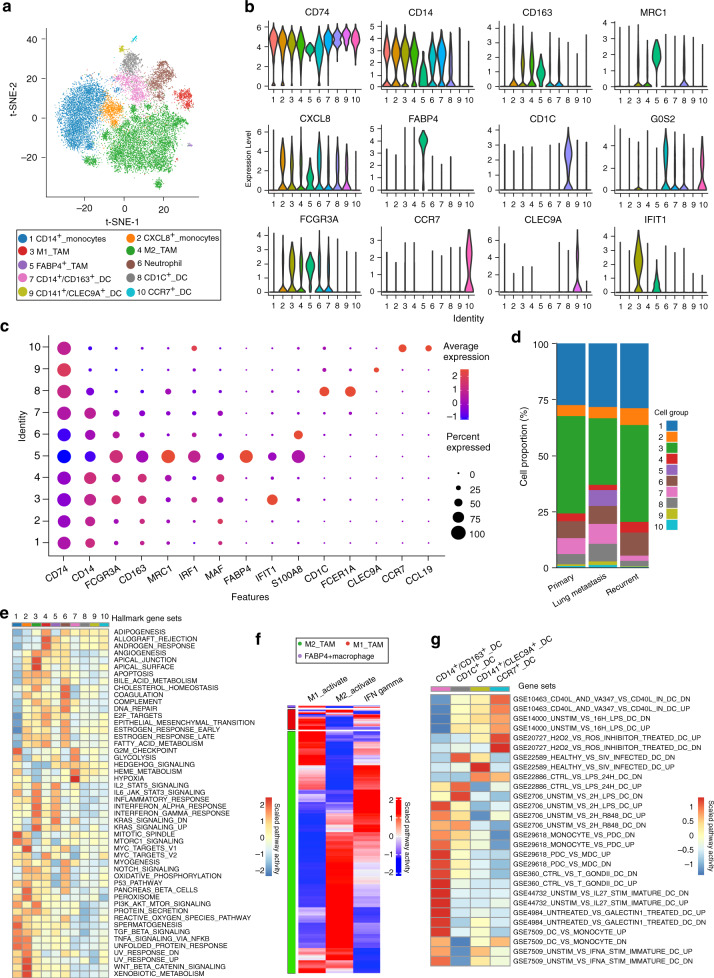
Comprehensive dissection of myeloid cells in OS lesions. **a** t-SNE plot separated 10 subclusters of the myeloid cells in OS lesions. **b** The violin plots showing the normalized expression levels of signature genes across the myeloid subclusters. **c** Dot plots showing cluster signature genes in myeloid cells. The size of dots represents the proportion of cells expressing the particular marker, and the spectrum of color indicates the mean expression levels of the markers (log1p transformed). **d** The proportion of the myeloid cells in the OS lesions with different types of lesions. **e** The heatmap of GSEA of 50 hallmark gene sets in MSigDB database between the 10 subclusters of myeloid cells. **f** Heatmap of the gene sets or signaling pathways specific for M1-activation, M2-activation, and IFN-γ activation for the macrophages in OS lesions, based on GSVA enrichment scores. **g** Heatmap of the gene sets or signaling pathways specific for each of the four subclusters of DC-activation based on GSEA enrichment scores with MSigDB database. The detailed cell proportion values of (**d**) and the relative GSEA scores for each gene set across the cell clusters of (**e**, **g**) are provided in the Source Data file.

Tumor-infiltrating myeloid cells are the critical abundant components of TME, which are a heterogeneous mixture of cell types with both tumor stimulating and suppressing activities^47^. Analysis of the myeloid cells revealed 10 distinct subclusters comprising monocytes, TAMs, DCs, and neutrophils (Fig. 6a–c; Supplementary Data 6). For OS lesions, the monocytes and the macrophages account for 70–80% of the total myeloid cells, while a minority of the cells (<5%) were identified as DCs (Fig. 6d**;** Supplementary Fig. 15a–c). We also identified specific gene sets for these myeloid cells that allow a more in-depth analysis of regulatory pathways (Fig. 6e, Supplementary Fig. 15d).

Three subsets of TAMs were identified in OS lesions, namely M1-, M2-, and M3-TAMs. It is known that M2-TAMs are the main tumor-associated anti-inflammatory macrophages with relatively high expression of *CD163, MRC1, MS4A4,* and *MAF* (Supplementary Fig. 15a). M1-TAMs are associated with inflammatory factors including *CCL2, CCL3, CCL4, CXCL2,* and *CXCL3*, which could attract NK cells, T cells and immature DCs in TME^48,49^. GSEA analysis suggested that M1-TAMs display enhanced activities of the signaling pathways stimulated by IFN-α, IFN-γ, IL2/STAT5, IL6/JAK/STAT3, and inflammatory responses, suggesting that these macrophages may be derived from a proinflammatory microenvironment in the OS lesions under IFN-α and IFN-γ stimulation (Fig. 6e). Interestingly, M1-TAMs showed elevated activities of the TGF-β and Hedgehog signaling pathways, which could induce the M2 polarization (Fig. 6e). Through the GSVA analysis, a small proportion of the M2-TAMs with relatively high expression levels of M1-TAMs marker genes was noticed (Fig. 6f), confirming the dynamic transformation between M1- and M2-TAMs in the TME of OS lesions. The M3-TAMs, actually the FABP4^+^ TAMs previously identified as the alveolar macrophages in the lung, were dominant in the lung metastatic OS lesion BC17 (Fig. 6d, Supplementary Fig. 15b, c)^50^. GSVA suggested that the FABP4^+^ TAMs in OS expressed remarkably high levels of M1 marker gene sets (Fig. 6f), indicating that the FABP4^+^ TAMs may contribute to the pro-inflammatory TME in lung metastasis OS cells. In addition, neutrophils characterized by *S100A8, S100A9,* and *G0S2* were detected (Fig. 6b, c). Our data displayed more neutrophils infiltrating the primary lesion than recurrent or lung metastatic lesions (Supplementary Fig. 15a–c); however, the clinical significance remains to be further addressed.

Recently, DCs, the sentinels of the immune system, were adopted as targets of immunotherapeutic treatment strategies due to their powerful antigen-presenting features. We identified four distinct subclusters of DCs in the OS lesions, the monocyte-derived CD14^+^CD163^+^ DCs, the conventional myeloid-derived CD1c^+^ DCs (cDC2), CD141^+^CLEC9A^+^ DCs (cDC1) lung and the activated CCR7^+^ DCs (Fig. 6g). CCR7 takes part in chemotaxis, survival, migratory speed, cytoarchitecture, and endocytosis of DCs, which are closely related to tumor metastasis^51^. We found that the ratio of CD1c^+^ DC was higher in lung metastatic lesions than the primary and recurrent lesions. CD1c^+^ DC has been administered as a source for vaccine immunotherapy that has shown encouraging immunological and clinical outcomes^52^. Thus, the infiltrated DCs in OS lesions may serve as immunotherapy targets in the future.

### Heterogeneity of the TILs

**Fig. 7:**
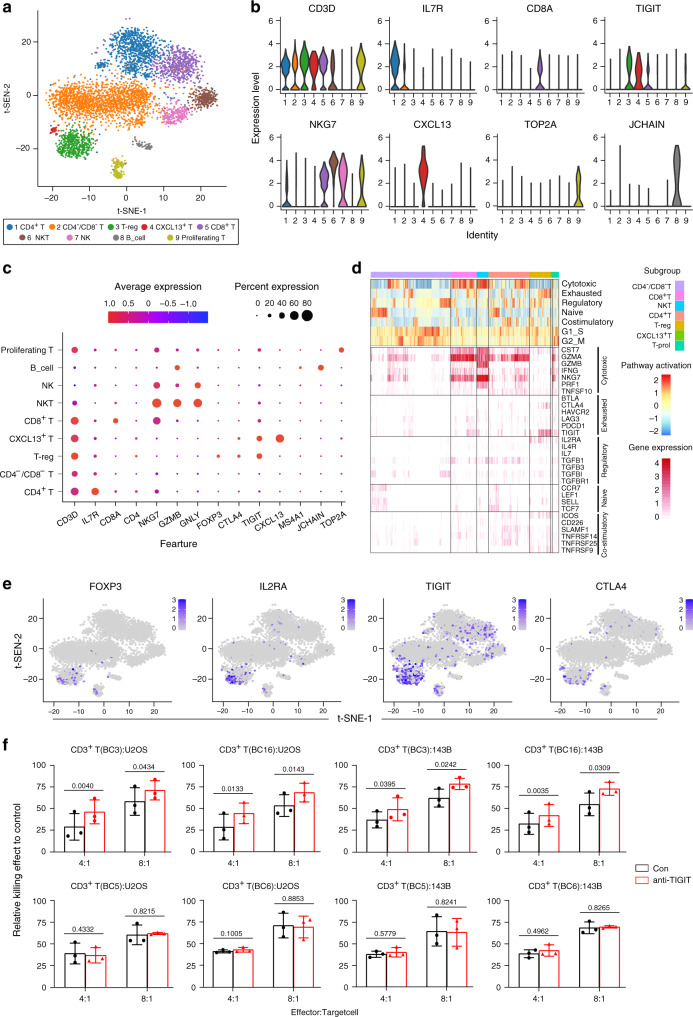
Cell clustering and functional annotation of tumor-infiltrating lymphocytes (TILs) in OS lesions. **a** t-SNE plot for TILs in OS lesions, and the cells were classified into seven subclusters. **b** The violin plots showing the normalized expression levels of 8 signature genes across the TIL subclusters. **c** Dot plots showing 14 signature genes among the TIL subclusters. The size of dots represents the proportion of cells expressing the particular marker, and the spectrum of color indicates the mean expression level of the markers (log1p transformed). **d** The t-SNE plot showing the expression profiles of the four selected well-known marker genes for exhausted T cells. **e** Heatmap of the gene sets of T-cell cytotoxicity, exhaustion, regulatory cytokines and receptors, naive T cells, and T-cell costimulation, based on GSVA enrichment analysis. **f** Blockade of TIGIT increases the CD3^+^ T cells mediated cellular cytotoxicity activities on U2OS and 143B cells derived from patients BC3 and BC16, but not for BC5 and BC6 (*n* = 3). Error bar: mean value ± SD. *P* values were determined by paired two-sided Student’s *t*-test. The source data for the relative T cellular cytotoxicity activities on OS cell lines (**f**) are provided in the Source Data file.

T cells are the key elements of cancer immunotherapy^53^; however, their high heterogeneity with respect to their cell-type compositions, gene expression patterns and functional properties significantly influence the outcomes of the T cells based immunotherapy^54^. From the OS lesions, we identified a total of 5420 TILs with high heterogeneity, which were classified into eight subclusters as CD4^−^CD8^−^ T, CD8^+^ T, CD4^+^ T, T-reg, proliferating T, NKT, NK, and B cells according to the specific markers (Fig. 7a–e; Supplementary Data 7). The cell number and proportion of each cellular subcluster was shown (Supplementary Fig. 16a–c). Lower proportion of CD4^+^ and CD8^+^ TILs was detected in the recurrent and metastatic OS lesions than in primary lesions, which has been validated by the IHC staining methods (Supplementary Fig. 16d). The low tumor-suppressive status in recurrent and metastatic OS lesions suggested that the T cell-based immunotherapy might be inefficient in the metastatic and recurrent OS patients.

The CD8^+^ T cells in the OS tissues were characterized with relatively high expression of the cytotoxicity markers granzyme A (*GZMB*), *GZMK,* and *GZMH*^55^ (Fig. 7d). Importantly, we also found that these cells positively expressed the T cell exhaustion inhibitory receptors including *TIGIT* and *LAG3*^56,57^ (Fig. 7d), suggesting that the CD8^+^ T cells become exhausted after the initial activation phase in OS. The CD4^+^ T cells expressed high cytotoxicity genes including *GZMA*^58^, and they also expressed relatively high levels of costimulatory molecules including *TNFRSF14, TNFRSF25*, and *ICOS5*^59^, which stimulate the cytotoxic activities of the T cells (Fig. 7d).

We noticed two cell subclusters expressing the NK cell markers including *NKG7* and *GNLY*. One subcluster expressing the T-cell specific markers including *CD3D* and *CD8A* was termed as the NKT cells, and the other subcluster was termed as the NK cells (Supplementary Fig. 16e). Most of the NKT cells were activated and strongly expressed the *GZMB, GZMA,* and *IFNG* genes^60^, indicating that they are performing tumor cytotoxicity activities in the OS (Fig. 7d). Interestingly, only a small fraction of the NK cells was positive for *GZMB, IFNG,* and *PRF1*, which could be the non-activated state of the NK cells in the OS lesions. In addition, we also identified a small proportion of B cells in the OS cells (87/5,420) with at least two subsets exclusively expressing the canonical markers *MS4A1* and *JCHAIN*, suggesting that even B cells, although very few, were involved in the OS lesion (Supplementary Fig. 16e, f).

Recently, the anti-TIGIT therapeutics have drawn great attention in treating colorectal cancer, breast cancer, and melanoma through modulating the activities of CD8^+^ T, T-reg, and NK cells^61^. Blockage of CD112R and TIGIT signaling sensitizes human natural killer cell cytotoxicity functions on melanoma^62^. The OS T-reg cells expressed the canonical gene signature including the *FOXP3* and *IL2RA* (Fig. 7d, e), and they also showed relatively high immune-inhibitory molecules including the *CTLA4* and *TIGIT*, which may contribute to T-reg cell-mediated suppression of anti‐tumor immune responses in OS lesions. We also noticed that *TIGIT* was widely expressed in CD8^+^ T, CD4^+^ T, and NKT cells in the OS (Fig. 7d, e), suggesting that *TIGIT* blocking could be an effective therapeutic method for OS. Further, we isolated peripheral blood CD3^+^ T cells from two patients (BC3 and BC16) with relatively high TIGIT^+^CD3^+^ T cells infiltration in OS tissues and two patients (BC5 and BC6) with relatively low TIGIT^+^CD3^+^ T cells infiltration in OS tissues to determine the cellular cytotoxic activities of the CD3^+^ T cells using anti-TIGIT antibodies in vitro. The results showed that blocking TIGIT substantially enhanced the death of OS cells triggered by CD3^+^ T cells derived from BC3 and BC16 in the co-culture system (*P* < 0.05, Fig. 7f). In contrast, anti-TIGIT treatment did not enhance the cytotoxicity of CD3^+^ T cells derived from patients BC5 and BC6 (*P* > 0.05, Fig. 7f). These suggested that targeting TIGIT may have potential therapeutic values for OS in the future.

## Discussion

Highly heterogeneous OS was characterized with complex SVs^63^, localized hypermutation, and abundant CNVs but relatively few point mutations on genomic level^64^. However, the current clinical studies could only reflect the average measurements of gene mutation and expression profiling across the tumor cells, and the cell-type composition, dynamics and characteristics in OS tumor lesions are largely undetermined. In this report we identified 11 main clusters of cells with t-SNE clustering in combination, contributing to OS lesions, and further analyzed the subclusters for seven of the main cell types (Supplementary Fig. 17). Their cellular and molecular features, regulators, and dynamics were also analyzed with regard to their role in the progression of OS. All these clusters of cells were progeny of adult stem cells either MSC or hematopoietic stem cells (HSCs). The current study dissected the complexity of the cellular landscape of the OS ecosystem encompassing the primary, lung metastasis and recurrent lesions based on scRNA-sequencing. The cellular atlas of malignant cells and TME components had revealed the intratumor heterogeneity characteristics, which may provide therapeutic targets for OS in the future.

The malignant osteoblastic cells may be originated from any type of cells along with the osteogenic differentiation linkage from MSCs. These cells were characterized with relatively high expression levels of mesenchymal markers *COL1A1, LUM, COL3A1*, and *RUNX2*, along with the transcriptional factors essential for osteoblast differentiation^65^. Based on the scRNA-seq data, the osteoblastic malignant cells were classified into six subclusters in light of the comprehensive gene expression atlas in tumorigenesis and oncogenesis gene expression atlas of the cells. The first were the two proliferating subclusters with the high expression levels of S and G2/M phase-related genes. The other four osteoblastic subclusters were characterized with differentially enhanced signaling pathways related to angiogenesis, *MYC, IFN-α, KRAS, TP53,* and other hallmark gene sets. Further, we found that the genes overexpressed in the lung metastasis and recurrence cells enriched Myc, mTORC1, hypoxia, and the oxidative phosphorylation signaling pathways. MYC and CCNE1 were the highly expressed or muted genes identified in the OS lesions^66,67^. The mTORC1 pathway was hyperactivated in clinical OS samples^68^, which is consistent with the scRNA-seq results. These results highlighted the intratumoral heterogeneity and the signaling pathways that may drive the progression and recurrence of the OS.

Chondroblastic OS ranks as the second commonly diagnosed (~25%) OS in children and adolescents^69^. The cellular origin of the chondroblastic OS is still debatable. It is important to note that there were malignant osteoblastic cells in the chondroblastic OS lesions based on H&E staining examination, which were further supported by the scRNA-seq that the cells expressing both *COL1A1* and *SOX9* were identified in the chondroblastic OS lesions BC20 and BC22. Previous study suggested that chondrocytes could undergo a direct transdifferentiation process into osteoblasts during the endochondral ossification in bone formation^70^. With the CNV clonal analysis by inferCNV with the scRNA-seq revealed a large scale genomic copy message, we found a great portion of genomic CNV pattern shared between the chondroblastic and osteoblastic malignant subclones in chondroblastic OS lesions. This result, together with the finding revealed in the trajectory analysis based on RNA expression profile at single cells level strongly suggests that transdifferentiation of malignant osteoblastic cells from malignant chondroblastic cells. Furthermore, with the cellular trajectory analysis, we demonstrated that during tumor cellular transdifferentiation in chondroblastic tumor cells, not only the genes involved in the osteoblast differentiation, ossification, and bone morphogenesis are significantly increased but also the genes related to histone methylation and acetylation. These results suggested that the epigenetic modifications may largely contribute to the transdifferentiation of malignant chondroblastic cells into osteoblastic cells.

In addition, we found both the chondroblastic and osteoblastic malignant cells were localized in the primary site while only the uniformed osteoblastic malignant cells were detected in the lung metastasis lesions of the chondroblastic OS of patient BC17, suggesting that the osteoblastic OS cells have more aggressive tumoral activities and are capable of distant metastasis compared to the corresponding chondroblastic tumor cells. This is supported by the reports that the malignant chondroblastic cells grew slow and were less sensitive to the chemotherapy treatments than malignant osteoblastic cells^71^. These results suggested that chemotherapy reagents that targeting both the chondroblastic and osteoblastic tumor cells are warranted for efficient treatment for chondroblastic OS in clinic.

Bone homeostasis reveals systematically subtle coordination of the osteogenesis and osteolysis^72,73^. There are clear imaging evidences supporting the contribution of osteolysis to bone remodeling in primary OS^74^. We noticed that the OCs were not only present in the primary and recurrent OS tumors but also in the lung metastasis OS lesion, further supporting that OC is essential and supportive for OS cell growth and dissemination, corroborating the previous^75^. Interestingly, the overall status and the subgroup cellular proportion of OCs were variable among the patients as annotated by the t-SNE results. According to the subcluster t-SNE analysis, mature OC was showed neither in the chondroblastic OS patients BC20 and BC22, nor in the osteoblastic OS patients BC11, and fewer total OC number was detected in lung metastatic lesions of patients BC10 and BC17. It is well known that osteoblast cells can stimulate the OC differentiation and activation through the RANKL/RANK signaling pathway^34^, suggesting that the OC maturation can be stimulated by the RANKL expressed by malignant osteoblast cells in OS tissue. Compared to osteoblastic cells, the chondroblast cells were found to have lower RANKL expression. And compared to the osteoblastic cells enriched in calcification zone in human growth plate^76^, fewer OCs were found in the chondroblastic cells enriched resting zone and hypertrophic zone. Therefore, the TME may hinder the OC maturation by the chondroblastic cells. In addition, chemotherapeutics may also hamper OC maturation in OS tissues. For example, the chemotherapeutic agent gemcitabine used in the treatment of OS patients was found to reduce the number of myeloid-derived OC progenitor cells^77^. In agreement with these reports, three patients in our study (BC10, BC11, and BC17) who received gemcitabine treatment showed relatively lower levels of total number of OCs and the mature status OCs. Altogether, it is reasonable to hypothesize that the TME and the chemotherapy treatments may modulate the OC maturation in OS tissues; however, studies are warranted to confirm these results. Further, through the cellular trajectory analysis of the OC maturation, we noticed the loss of antigen-presenting activities and the gain and elevated osteolysis activities from the OC progenitor cells to the mature OC cells, suggesting a switch from tumor-suppressive activities to tumor-promoting activities during the OC maturation. Interactions between OCs and the immune system encourages the identification of new therapeutic targets for OS treatments.

The immune cells, as the significant component of the TME usually show the immunosuppressive activities^78^. The TME consists of various types of cells mediating the communication between malignant cells, immune cells and stromal cells^79^. TAMs, especially the M2-TAMs relatively express high *CD206* and *MRC1*, and play critical roles in tumor growth, angiogenesis, invasion and metastasis especially^80^. In the present study, we identified three distinct TAM populations in OS, and the majority TAMs were M2. Interestingly, we have identified alveolar *FABP4*^+^ macrophage in lung metastatic OS tissues, which showed proinflammation properties as suggested by the GSVA analysis. These results suggested that the tissue-resident macrophages may also involve in the OS progression; however, their roles and the underlying mechanisms need further investigation.

T cell immune checkpoint molecules are a set of promising immunotherapeutic targets for cancer^81^. In the OS lesions studied here, seven subclusters of T cells were identified and their expression features of gene sets related to T cell cytotoxicity, exhaustion, regulation, naive and co-stimulation were assessed using GSVA enrichment analysis. T cells with predominant exhaustion signature are the major subpopulation in TME of OS, which was also commonly noticed in the other cancer tissues^82^. Our data depicted that T cell-mediated tumor-suppressive activities in the TME is weaker in lung metastasis and recurrent lesions compared to primary lesions. Of the immune checkpoint molecules, we noticed a relatively low mRNA level of *PDCD1, HAVCR2* and *LAG3* in the CD3^+^ T cells, whereas the *TIGIT* showed the most abundant expression levels in T-reg cells. Recently, the anti-TIGIT therapeutics have got great attention as a therapeutic marker of check point through modulating the activities of CD8^+^ T, T-reg and NK cells activities^83–86^. In the current scRNA-seq data, *TIGIT* was widely expressed in T-reg, CD8^+^ T, CD4^+^ T, and NKT cells in OS, suggesting that OS patients may benefit from TIGIT blocking therapeutics. Our experiments with the in vitro cellular models using TIGIT blockage antibody reinforced the cytotoxicity of CD3^+^ T cells against OS cells, providing a primary evidence supporting the potential of TIGIT inhibition for OS treatments in future.

Several limitations in the current study should be acknowledged. First, the sample size was relatively small, particularly only two samples were collected for the recurrent and the lung metastatic lesions, without matched primary OS samples due to the clinical nature of OS. We realized significantly cellular heterogeneity between the patients, an independent comparison with a panel of paired samples or more samples may help to further validate the results and to eliminate any possible bias due to heterogeneity between the individuals. Second, all patients had received combined chemotherapy before surgical operation according to the National Comprehensive Cancer Network (NCCN) treatment guidelines^87^, and the influence of the gene expression pattern of the cells by chemotherapy was unable to determine. However, these results reflected the status of OS cells and TME that encountered in clinic practice, especially for the advanced OS samples, which may provide therapeutic targets in future. Third, the scRNA-seq was performed with 10× Genomics chromium platform, which usually gives a relatively lower gene coverage for a larger number of single cells compared to the plate-based scRNA-seq methods (such as the Smart-seq). Smart-seq method may generate more refined cellular atlas of OS with deeper message of transcripts. At last but not least, a set of markers were identified in this study, exemplified by the TIGIT blocking therapeutics, are yet to be intensively studied with more models.

In conclusion, the current scRNA-seq analysis demonstrated the intratumoral heterogeneity of OS cells and their TME in OS tissues. Distinct clusters and subclusters for various types of cells in OS lesions were identified with their corresponding molecular features determined. The scRNA-seq revealed the cellular lineage transdifferentiation of osteoblastic cells from chondroblastic cells in OS tissues. Meanwhile, the cellular constitutions of immune and stromal cell types and their properties suggesting the immunosuppressive and tumor progression supportive activities in OS tissues. This study provided the preliminary cellular atlas for OS tissue, and paved a way for the identification of therapeutic targets to improve the OS treatment.

## Methods

### Participants

Eleven patients hospitalized from October 2017 to April 2019 in Shanghai Sixth People’s Hospital were prospectively enrolled in the study, which was approved by the Shanghai Sixth People’s Hospital Ethics Committee. Each patient provided written informed consent. All 11 patients (five male and six female, age range from 11 to 38-years old) had been diagnosed with osteoblastic or chondroblastic OS according to the NCCN Clinical Practice Guidelines in Oncology (https://www.nccn.org/). Samples for scRNA-seq were derived from the primary tumor sites of seven patients who had received traditional first-line adjuvant and neo-adjuvant chemotherapy composed of a cocktail of four drugs (doxorubicin, cisplatin, methotrexate and ifosfamide), as well as surgical therapy. Two patients with lung metastasis and two with recurrent disease had received gemcitabine in combination with docetaxel (GT). In addition to other patients enrolled for our study, we recruited one patient (BC17) from the clinical trial NCT03676985, in which all patients had undergone neoadjuvant chemotherapy, surgery, and adjuvant chemotherapy, and they all had received anti-PDL-1 therapy for one year until the disease progressed. Patient BC17 provided written informed consent to participate in the clinical trial NCT03676985 and the current study. Four patients BC3, BC5, BC6, and BC16 agreed to donate peripheral blood to explore the efficacy of anti-TIGIT therapy in vitro. Detailed clinical characteristics information about patients is provided (Supplementary Table 1).

### Sample preparation and cell isolation for scRNA-seq

Fresh tumor lesions were stored in GEXSCOPETM tissue preservation solution (Singleron Bio Com, Nanjing, China) and processed on ice after the surgery within 30 mins. The specimens were washed with Hanks Balanced Salt Solution (HBSS) three times and minced into 1–2 mm pieces. Then, the tissue pieces were digested with 2 mL of GEXSCOPETM tissue dissociation solution (Singleron) at 37 °C for 15 min with sustained agitation. After digestion, the samples were filtered through 40-µm sterile strainers and centrifuged at 800 × *g* for 5 min. Subsequently, the supernatants were discarded, and the cell pellets were suspended in 1 mL phosphate-buffered saline (PBS; HyClone, United States). To remove red blood cells, 2 mL of GEXSCOPETM red blood cell lysis buffer (Singleron) was added, and cells were incubated at 25 °C for 10 min. The solution was then centrifuged at 500 × *g* for 5 min and resuspended in PBS. The samples were stained with trypan blue (Sigma, United States) and the cellular viability was evaluated under the phase contrast light microscope (Nikon, Japan).

### Library preparation and scRNA-seq

Single cells were encapsulated into emulsion droplets using the Chromium Controller (10× Genomics). The scRNA-seq libraries were constructed using the Chromium Single Cell 3ʹ Library, Gel Bead & Multiplex Kit (10× Genomics, V2 and V3) following the manufacturer’s instructions. In brief, the sample volume was decreased, and the cells were examined with a light microscope and counted with a hemocytometer. Cells were loaded in each channel with a target output of ~5000 cells. The cells were partitioned into Gel Beads in Emulsion in the Chromium^TM^ Controller instrument where cell lysis and barcoded reverse transcription of RNA were performed. The sequencing libraries were constructed using the amplified cDNA with the Nextera XT DNA sample Pre-Kit (FC-131-1024, Illumina), and final libraries of individual samples were evaluated on the Agilent Bioanalyzer using a High Sensitivity DNA Kit (Agilent Technologies). Individual libraries were pooled for sequencing with 75 cycle run kits on the Illumina HiSeq X platform with 150-bp paired-end reads.

### Pre-processing of scRNA-seq data

Raw reads were processed to generate gene expression profiles using the standard internal pipeline based on the Cell Ranger toolkit (version 2.1.1). The raw base call (BCL) files were used to generate the FASTQ files with the “mkfastq” command. After filtering read 1 without poly T tails, cell barcode and unique molecular identifiers (UMIs) were extracted. Adapters and poly A tails were trimmed (fastp V1) before aligning the read 2 to GRCh38 Ensemble build 92 genome (fastp 2.5.3a and featureCounts 1.6.2). Reads with the same cell barcode, UMIs and gene were grouped together to calculate the number of UMIs per gene per cell using the “count” command. The UMI count tables of each cellular barcode were used for further analysis.

The raw output data were processed with the Seurat package (version 3.1.5; http://satijalab.org/seurat/) in R software (version 3.6.1) for each individual sample^88^. We filtered out the cells with no. of expressed genes <300 genes or the percent of mitochondrial genes over 10% of total expressed genes. Further, we removed the potential doublets (and to an even lesser extent of higher-order multiplets) that occurred in the encapsulation step and/or as occasional pairs of cells that were not dissociated in sample preparation using the DoubletFinder package (version 2.0.2) of the R^89^. A total of 10,987 filtered cells were used for further bioinformatic analysis.

### Data integration and the dimensionality reduction

The Seurat object with gene expression data from individual samples was processed with the Read10× () function. For each sample, the gene expression was represented as the fraction of the gene and multiplied by 10,000, which were converted into natural logarithm and normalized after adding 1 to avoid taking the log of 0. The top 3000 highly variable genes (HVGs) from the normalized expression matrix were identified, centered, and scaled before we performed the principal component analysis (PCA) based on these HVGs. The batch effects were removed by the Harmony package (version 1.0) of R based on the top 50 PCA components identified^90^.

### Cell-clustering and annotation

The clustering analysis was performed based on the integrated joint embedding produced by Harmony with the Louvain algorithm after computing a shared nearest-neighbor graph with the Louvain algorithm that was implanted in the “FindClusters” function of the Seurat package. The identified clusters were visualized on the 2D map produced with the t-distributed t-SNE or UMAP method. For sub-clustering analysis, we applied a similar procedure including the variable genes identification, dimension reduction, cell integration with Harmony and the clustering identification to the restricted cluster derived from the overall analysis. To annotate the cell clusters, DEGs with high discrimination abilities between the groups were identified with the FindAllMarkers() function in Seurat using the default non-parametric Wilcoxon rank sum test with Bonferroni correction. The cell groups were annotated based on the DEGs and the well-known cellular markers from the literature. Detailed information of the cellular biomarkers was provided in Supplementary Table 2.

### DEGs identification and GO enrichment analysis

We identified the differentially over-expressed genes in the specific cluster when compared to other remaining clusters with the Wilcoxon Rank-Sum Test with the FindMarkers function in Seurat (adjusted *P*-value < 0.05, only.pos = T and logfc.threshold = 0.1), and the cluster-specific overrepresented GO biological process was calculated with the compareCluster function in the clusterProfiler package (version 3.14.3) of R^91^. We also used the GSEA with the curated gene sets to identify the pathways that were induced or repressed in between the cell clusters. The gene set enrichment was performed following the modification of the competitive gene set enrichment test CAMERA developed by Cillo et al.^92^ that have been embedded in the SingleSeqGset (version 0.1.2) R package. In brief, the mean gene expression level was calculated and the log twofold change (FC) between the specific cell cluster and the other cells was applied as the test statistic^92^. The 50 hallmark gene sets in the MSigDB databases (https://www.gsea-msigdb.org/gsea/msigdb) were used for the GSEA analysis^93^.

We also applied the non-parametric and unsupervised algorithm named gene set variation analysis (GSVA) to assess the relative pathway activities in the T, macrophage, and the DC cells. The signature gene lists of T cell (cytotoxic, exhausted, regulatory, naive, costimulatory, G1/S and G2/M) and the tumor macrophage (M1- or M2- type) were derived from the study performed by Chung et al.^94^. The gene set lists for the IFN-γ signaling pathway activity (HALLMARK_INTERFERON_GAMMA_RESPONSE) in T cells and the DC activities were derived from the MSigDB collections as indicated^93^.

### Single-cell copy-number variation (CNV) and clonality analysis

Initial CNVs for each cell in the osteoblastic and chondroblastic tumor cells were estimated with the inferCNV package of R (version 1.2.2; https://github.com/broadinstitute/inferCNV/wiki)^24^. The CNVs of osteoblastic and chondroblastic tumor cells were calculated and the immune cells were applied as the reference. After filtering the unqualified cells with < 2000 UMIs, the inferCNV analysis was performed with parameters including “denoise”, default hidden markov model (HMM) settings, and a value of 0.1 for “cutoff”. To reduce the false positive CNV calls, the default Bayesian latent mixture model was implemented to identify the posterior probabilities of the CNV alterations in each cell with the default value of 0.5 as the threshold. To infer the clonal single-cell CNV changes, the “subcluster” method was applied to infer the subcluster cells based on the CNV values generated by HMM. Consulting with the genomic cytoband information, each p- or q-arm level change was simply converted to equivalent CNV based on its location. Each CNV was annotated to be either a gain or a loss. After data conversion, subclones containing identical arm level CNVs were collapsed and trees were restructured to represent subclonal CNV architecture. For data visualization, we followed the UPhyloplot2 algorithm developed by Durante et al.^28^ (https://github.com/harbourlab/UPhyloplot2) to automate generation of intra-tumor evolutionary trees. The arm level CNV calls curated from the inferCNV HMM subcluster CNV predictions algorithm and the percentage of cells in each of the subclones were used as inputs. A scalable vector graphics (.svg) file visualizing the phylogenetic tree was generated for each sample and the arm length is proportional to the percentage of cells plus a spacer (circle diameter + 5 pixels)^28^.

### Trajectory analysis of single cells

The single-cell pseudotime trajectories were generated with the Monocle2 package (v2.8.0) in R^95^. The gene-cell matrix in the scale of raw UMI counts derived from the Seurat processed data were used as the inputs. The newCellDataSet function was applied to create an object with the parameter expressionFamily = negbinomial.size. Only genes with the mean expression ≥ 0.1 were used in the trajectory analysis. DEGs with *q*-value < 0.01 between the cell groups were applied for dimension reduction the reduceDimension() function using the parameters reduction_method = “DDRTree” and max_components = 2. The cells were ordered and visualized with the plot_cell_trajectory() function. Genes that changed along with the pseudotime were calculated (*q*-val < 10^−10^) and visualized with the plot_pseudotime_heatmap and the genes were clustered into subgroups according to the gene expression patterns. To identify the genes that separate cells into branches, the branch expression analysis modeling (BEAM) analysis were performed and genes resulting from the BEAM analysis with a *q*-value < 10^−10^ were separated into groups and visualized with the plot_genes_branched_heatmap() function. The enrichment GO terms of the genes in each cluster were calculated with the clusterProfilter (version 3.14.3) package^91^. Differentially expressed transcriptional factors were retrieved from the DEGs list based on the AnimalTFDB (v3.0) database (http://bioinfo.life.hust.edu.cn/AnimalTFDB/#!/).

To validate the results from the Monocle 2, the slingshot algorithm, another popular trajectory analysis tool that fits the bifurcation trajectory, was reanalyzed the cellular trajectory of chondroblast OS cells^31^. The SCORPIUS algorithm, an unsupervised approach for inferring linear developmental chronologies from single-cell RNA-sequencing data^32^, was performed to validate the Monocle 2 inferred linear transition of OCs from the OC progenitor cells and OCs immature cells into mature OCs in OS tissues.

### Immunohistochemistry and immunofluorescence staining

Tissue sectioning and immunohistochemistry staining of formalin-fixed, paraffin-embedded OS specimens were performed. All sections were deparaffinized, rehydrated, and washed. Endogenous peroxidase was blocked using 3% hydrogen peroxide for 10 min. After water-bath heating for antigen retrieval, slides were incubated with primary antibodies followed by horseradish peroxidase (HRP)-linked secondary antibodies and diaminobenzidine staining (ready-to-use #ZLI-9018, ZhongShan Golden Bridge Biotechnology, China). Counterstaining was done with hematoxylin. Slides were dehydrated with sequential ethanol washes (75%, 80, and 100%) for 1 min each. Two pathologists blinded to clinical data independently assessed staining results for TIGIT (1:200, #ab243903, Abcam, USA), CD3 (1:100, #ab5690, Abcam), CD4 (1:50, #ab213215, Abcam), CD8 (1:50, #ab17147, Abcam), CD74 (1:200, #ab22603, Abcam) and CTSK (1:200, #ab37259, Abcam).

For immunofluorescence staining, the process was similar up to the incubation with primary antibodies of CD74 (1:200) and CTSK (1:200), which was performed overnight at 4 °C. Samples were incubated for 1 h at room temperature after washing with fluorescently labeled secondary antibodies including donkey anti-rabbit Alexa Fluor 488 (#A21202, 1:1000; Molecular Probes) and goat anti-mouse Alexa Fluor 514 (#A31555, 1:1000; Molecular Probes). Nuclei were counterstained with 4’,6-diamidino-2-phenylindole (DAPI; #D9542, Millipore Sigma). Sections were mounted using fluorescence mounting medium (#S3023, Dako, Denmark).

### CytoTox 96® non-radioactive cytotoxicity assay

Peripheral blood mononuclear cells (PBMCs) were collected from two OS samples (BC3 and BC16) using density centrifugation with Lymphocyte Separation Medium (MP Biomedicals). Then CD3^+^ T cells were isolated with magnetic-activated cell sorting system (MACS; Miltenyi Biotec) according to the manufacturer’s protocol. For the T-cell activation assays, CD3^+^ cells were seeded in 24-well plates and stimulated for three days with interferon-γ (IFN-γ, 1000 U/mL; Peprotech), IL-2 (600 U/mL, Peprotech), and anti-CD3 antibody (5 ng/mL, clone OKT3; Biolegend). Subsequently, TIGIT was blocked for 24 h with anti-TIGIT antibodies (50 μg/mL, clone #A15153G, Biolegend). 143B and U2OS cells were seeded in 96-well plates, incubated overnight, and then added to CD3^+^ T cells at effector-to-target (E:T) ratios of 4:1 and 8:1. Co-culture systems were incubated for 8 h. The supernatant was harvested and analyzed using the CytoTox 96® non-radioactive cytotoxicity assay (Promega, CTB163, USA). Each group had three parallel wells, and all experiments were performed at least three times^96^. According to the manual, the killing effect of T cells against target cells was assessed with the following equation: Cytotoxicity = (Experimental–Effector Spontaneous–Target Spontaneous)/(Target Maximum–Target Spontaneous) × 100%.

### Statistical analysis

The statistical analysis was performed using SPSS 21.0 (IBM, Armonk, NY, USA). Continuous data were expressed as mean ± standard deviation (SD). The significance of differences was determined using the unpaired or paired Student’s *t*-test as indicated, and differences with *P* < 0.05 were considered as statistically significant.

### Reporting summary

Further information on research design is available in the Nature Research Reporting Summary linked to this article.

## Supplementary information

Supplementary Information

Peer Review File

Description of Additional Supplementary Files

Supplementary Data 1

Supplementary Data 2

Supplementary Data 3

Supplementary Data 4

Supplementary Data 5

Supplementary Data 6

Supplementary Data 7

Reporting Summary

## Data Availability

The Single-cell expression data have been deposited in the NCBI Gene Expression Omnibus database under the accession code GSE152048. All the other data supporting the findings of this study are available within the article and its supplementary information files without any restrictions or from the corresponding author upon reasonable request. A reporting summary for this article is available as a Supplementary Information file. Source data are provided with this paper.

## Source data

Source Data
