# Author Correction: Single-cell RNA landscape of intratumoral heterogeneity and immunosuppressive microenvironment in advanced osteosarcoma

**DOI:** 10.1038/s41467-021-23119-7

**Published:** 2021-04-30

**Authors:** Yan Zhou, Dong Yang, Qingcheng Yang, Xiaobin Lv, Wentao Huang, Zhenhua Zhou, Yaling Wang, Zhichang Zhang, Ting Yuan, Xiaomin Ding, Lina Tang, Jianjun Zhang, Junyi Yin, Yujing Huang, Wenxi Yu, Yonggang Wang, Chenliang Zhou, Yang Su, Aina He, Yuanjue Sun, Zan Shen, Binzhi Qian, Wei Meng, Jia Fei, Yang Yao, Xinghua Pan, Peizhan Chen, Haiyan Hu

**Affiliations:** 1 Oncology Department of Shanghai Jiao Tong University Affiliated Sixth People’s Hospital, Shanghai, 200233 China; 2 Orthopaedic Department of Shanghai Jiao Tong University Affiliated Sixth People’s Hospital, Shanghai, 200233 China; 3 Central Laboratory of the First Hospital of Nanchang, Nanchang, 330008 China; 4 Pathology Department of Shanghai Jiao Tong University Affiliated Sixth People’s Hospital, Shanghai, 200233 China; 5 Department of Orthopedic Oncology, Changzheng Hospital of Naval Military Medical University, Shanghai, 200003 China; 6 MRC Centre for Reproductive Health & Edinburgh Cancer Research UK Centre, Queen’s Medical Research Institute, EH16 4TJ Edinburgh, United Kingdom; 7 Department of Biochemistry and Molecular Biology, School of Basic Medical Sciences, Southern Medical University, Guangzhou, 510515 China; 8 Guangdong Provincial Key Laboratory of Single Cell Technology and Application, Guangzhou, 510515 China; 9 Department of Biochemistry and Molecular Biology, Medical College of Jinan University, 601 Western Huangpu Avenue, Guangzhou, 510632 China; 10 Clinical Research Center, Ruijin Hospital, Shanghai Jiao Tong University School of Medicine, Shanghai, 201821 China

**Keywords:** Bone cancer, Cancer microenvironment, Tumour heterogeneity, Sarcoma

Correction to: *Nature Communications* 10.1038/s41467-020-20059-6, published online 10 December 2020.

The original version of this Article contained an error in Fig. 4a. The labels of the t-SNE plot in Fig. 4a incorrectly corresponded to those of Fig. 4c.

This has been corrected in both the PDF and HTML versions of the Article.

