# Supplementary material for: Single-cell RNA landscape of intratumoral heterogeneity and immunosuppressive microenvironment in advanced osteosarcoma
Source: Nat Commun. 2020 Dec 10;11:6322. doi: 10.1038/s41467-020-20059-6 (PMC7730477; doi:10.1038/s41467-020-20059-6)
Supplement: Supplementary file 1 — Supplementary Information [file 41467_2020_20059_MOESM1_ESM.pdf]

Single-cell RNA landscape of intratumoral heterogeneity and immunosuppressive microenvironment in advanced osteosarcoma

Supplementary Information

Supplementary Figure 1

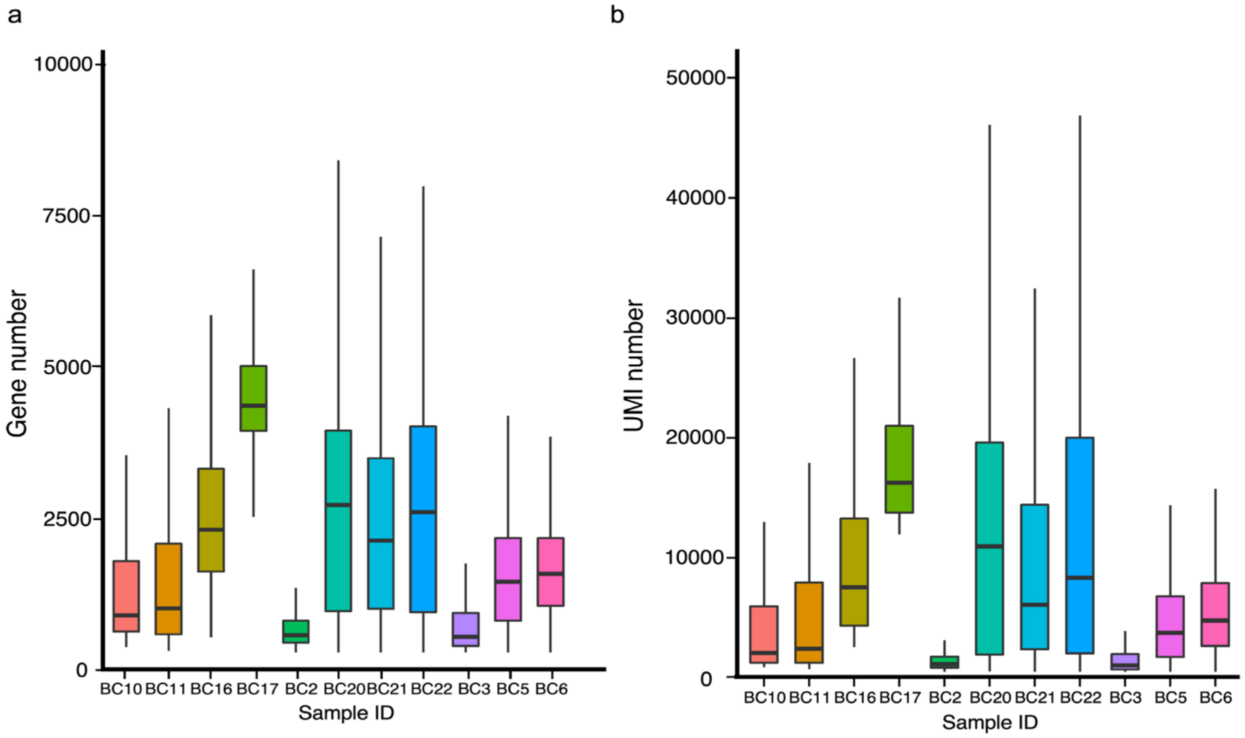

Supplementary Fig. 1. Detected gene numbers and total UMIs of each patient. a, b The box plot showing the distribution of detected gene numbers (**a**) and total UMIs per cell (**b**) of the single cells in each of the 11 OS patients (BC2, n = 5,297; BC3, n = 7,268; BC5, n = 15,817; BC6, n = 18,396; BC10, n = 14,404; BC11, n = 8,580; BC16, n = 8,412; BC21, n = 3,935; BC17, n = 3,331; BC20, n = 8,777 and BC22, n = 6,770). Each box indicates the interquartile range (IQR, the range between the 25th and 75th percentile) with mid-point data, and the whiskers represents the upper and lower value within 1.5 times the IQR.

Supplementary Figure 2

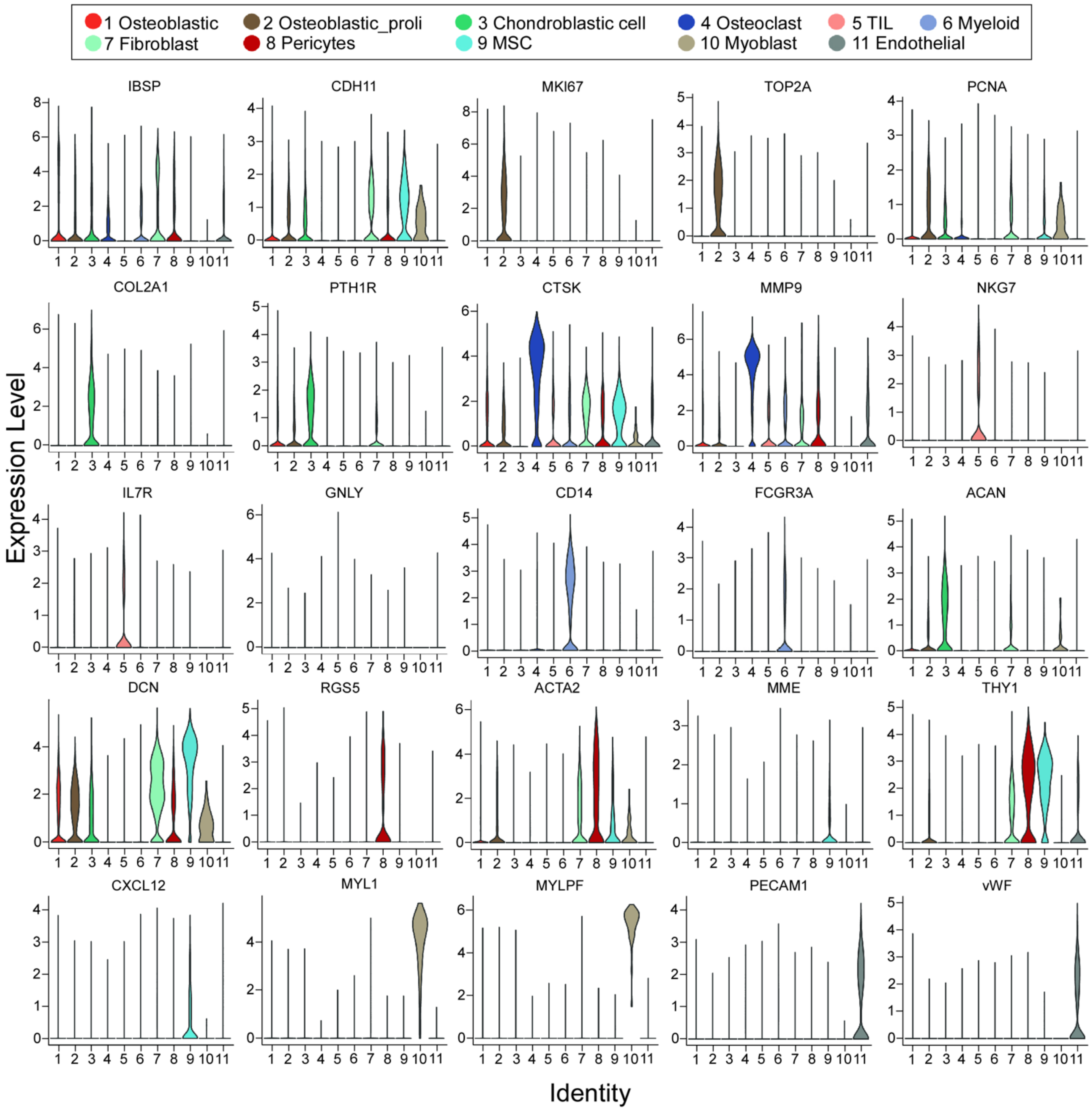

Supplementary Fig. 2. Expression level of canonical biomarkers of the subclusters. The violin plot showing the normalized expression levels of 25 specific signature genes for each of the 11 clusters as indicated.

Supplementary Figure 3

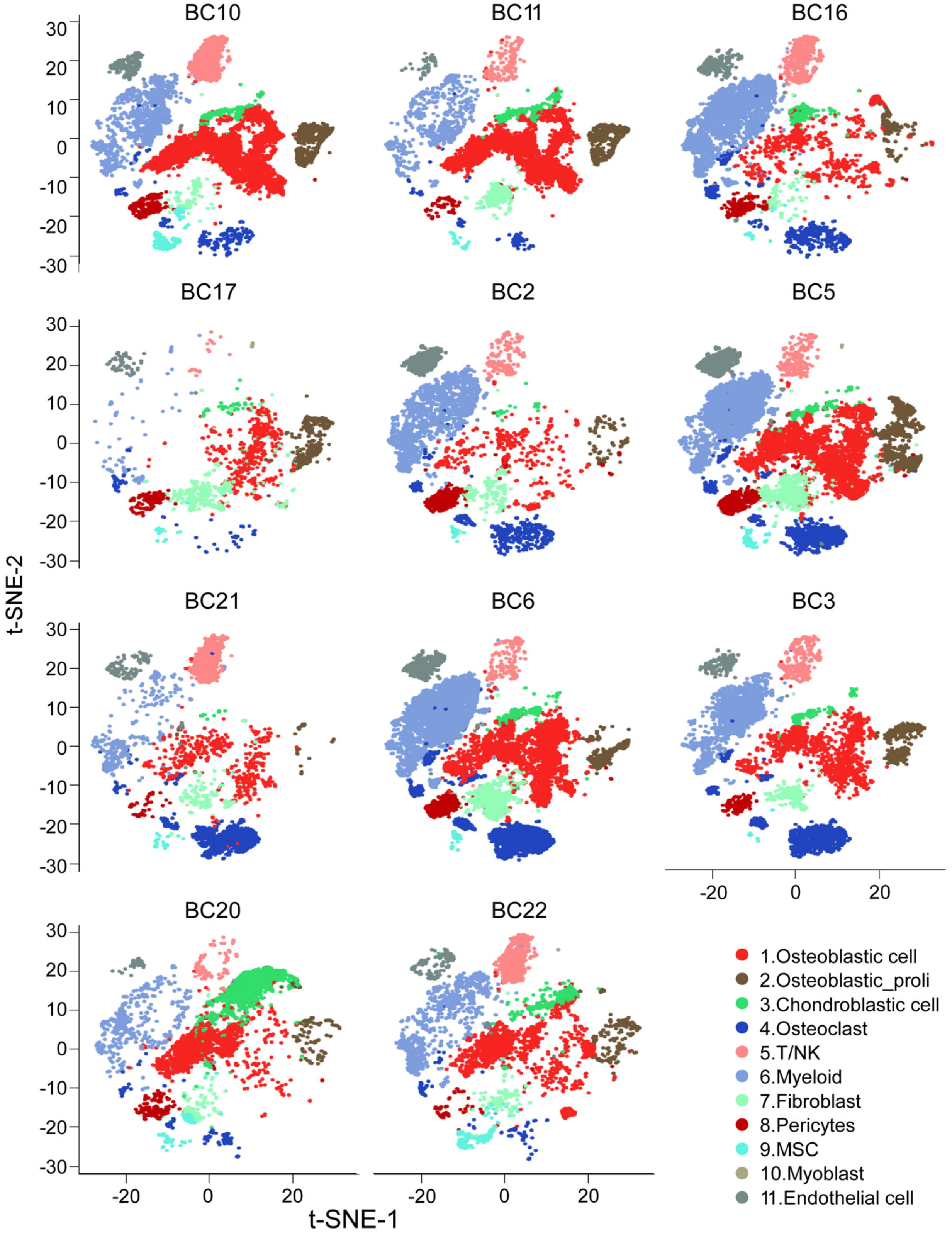

Supplementary Fig. 3. t-SNE plot of the identified cell clusters. t-SNE plot showing the distribution of the 11 cellular clusters in each OS sample, color-coded by cell subclusters.

Supplementary Figure 4

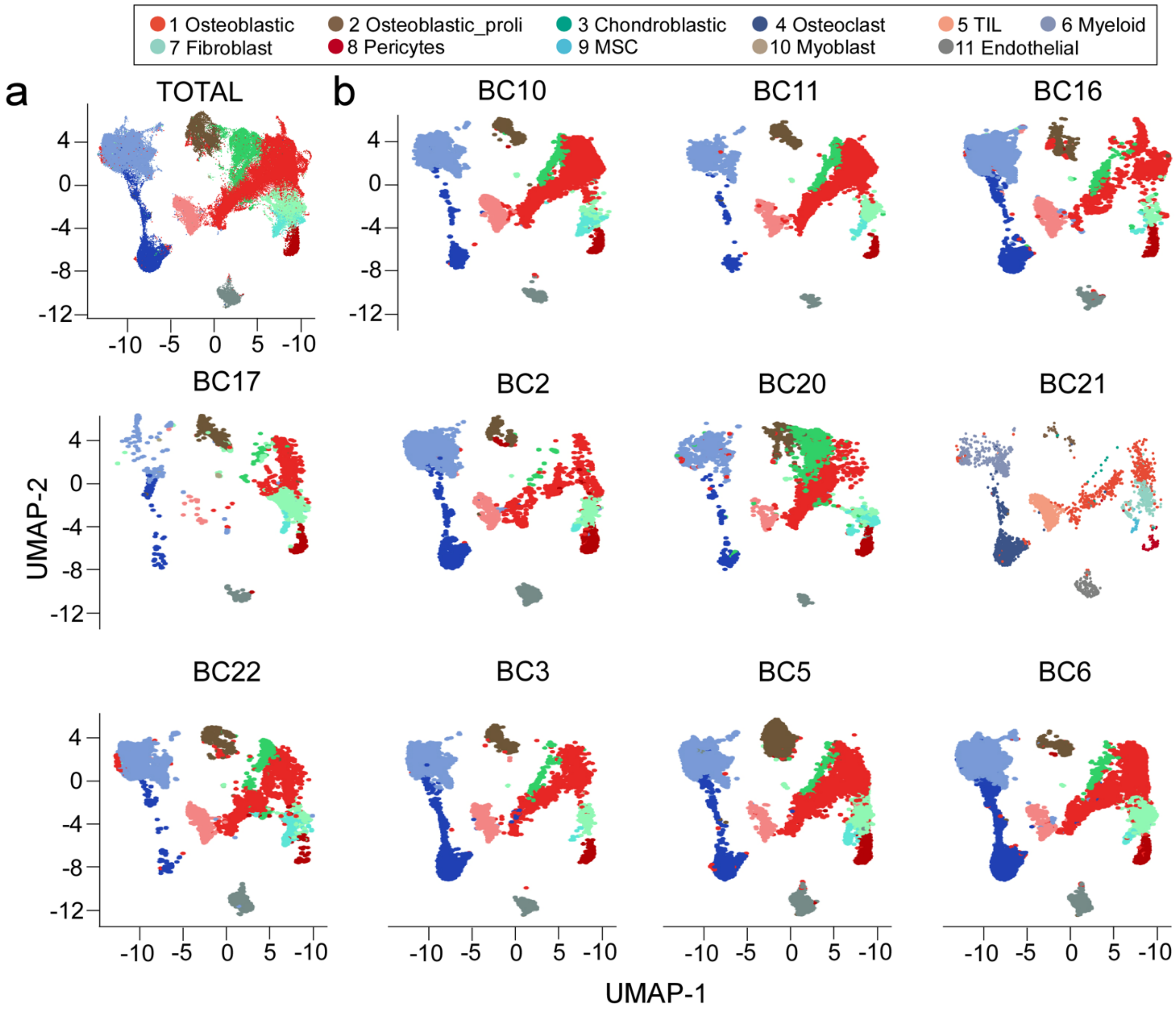

Supplementary Fig. 4. UMAP plot of the identified cell clusters. a, b The UMAP plot showing the distribution of the 11 cellular clusters in the combined (**a**) and each OS sample (**b**), color-coded by cell subclusters.

Supplementary Figure 5

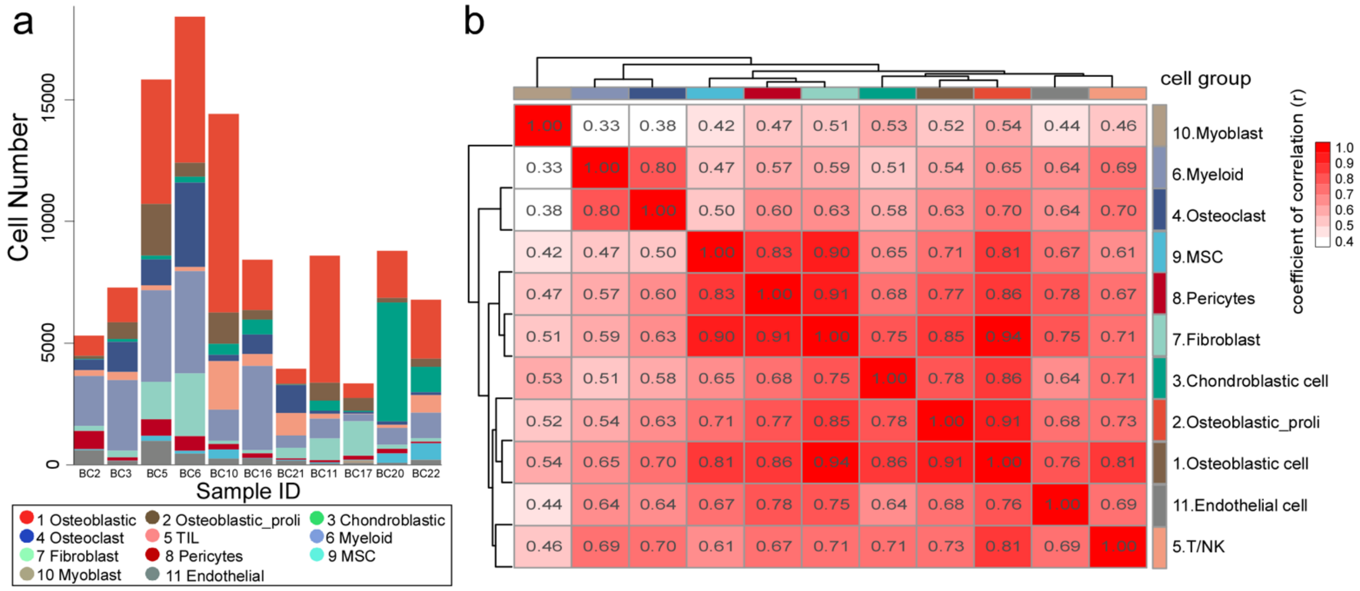

Supplementary Fig. 5. The cellular cluster distribution and the gene expression correlation among the OS samples. **a** The bar chart showing the cell numbers of the 11 subclusters detected in each of the 11 OS samples. **b** The Pearson's correlation among the 11 clusters based on the average gene-expression profiles of the top 3000 highly variable genes among the clusters. The color key (from white to red) indicates the value of the Pearson's correlation coefficient (r).

Supplementary Figure 6

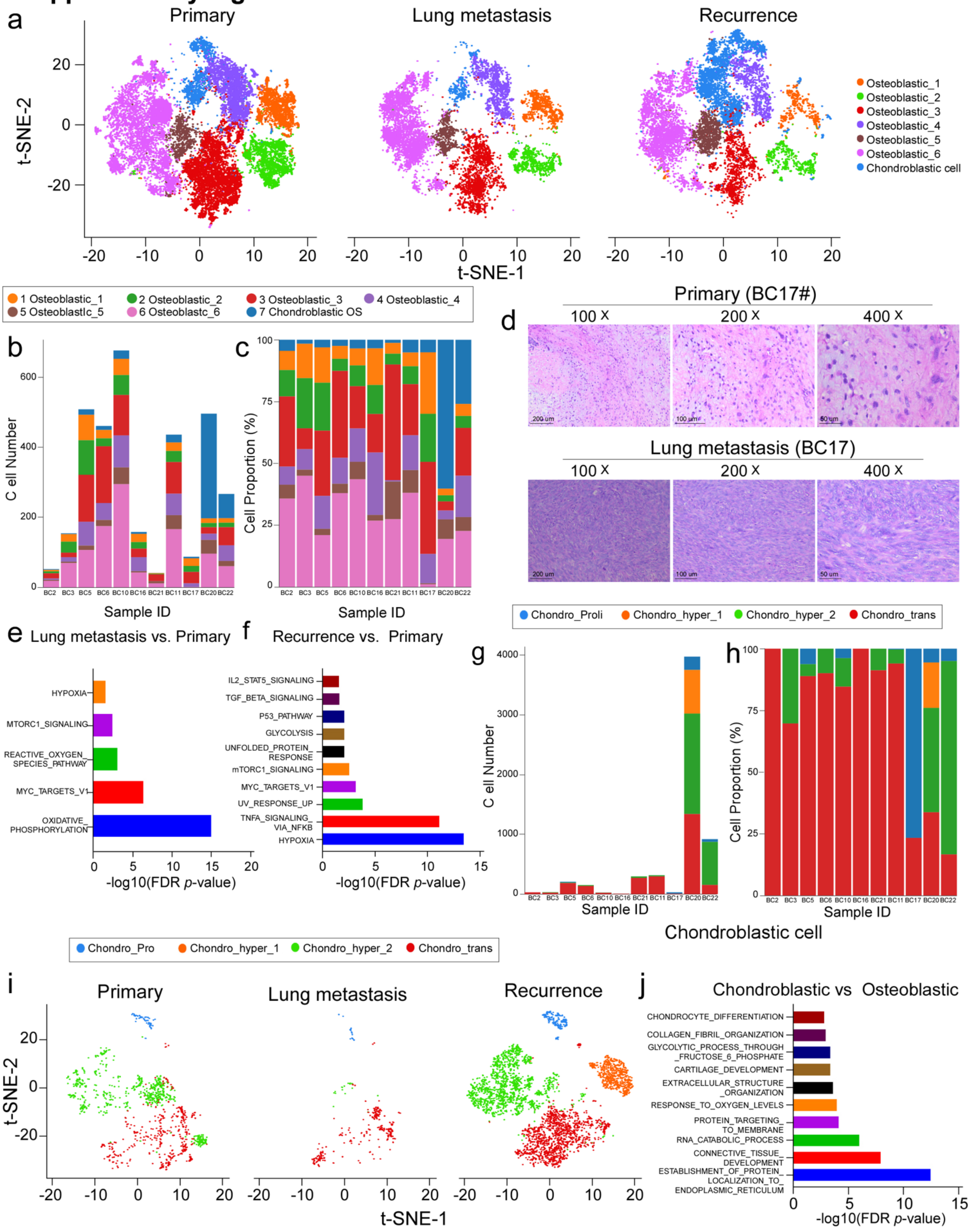

Supplementary Fig. 6. Intratumoral heterogeneity of OS cells in 3 different types of lesions. a t-SNE plot showing the 7 malignant cell subclusters from primary, lung metastasis and recurrent tumor lesions. **b, c** The bar chart shows cell number (**b**) and percentage (**c**) of the seven malignant subclusters of each OS samples (n = 11). **d** Hematoxylin-eosin staining of the 2 types of OS lesions, the lung metastasis OS lesion (BC17) and its counterpart primary lesion (here labeled as BC17#, which was lost early and not studied in any other way). **e, f** The gene ontology (GO) biological process enrichment analysis for the differentially expressed genes between the lung metastasis (**e**), recurrent (**f**) versus primary lesion. The *x*-axis indicates the -log10 transformed false-discovery rate (FDR) adjusted p-values. **g, h** The cell number (**g**) and proportion (**h**) of chondroblastic subclusters in individual patients as indicated. **i** t-SNE plot showing the 4 chondroblastic subclusters in the 3 types of OS lesions. **j** The GO biological process enrichment analysis for the genes differentially expressed between the chondroblastic versus osteoblastic tumor lesions. The *x*-axis indicates the -log10 transformed FDR adjusted p-values of GO analysis. Source data indicating the distinct cellular number (**b, g**) or proportion (**c, h**) of the malignant osteoblastic or chondroblastic cells are provided in the Source Data file.

Supplementary Figure 7

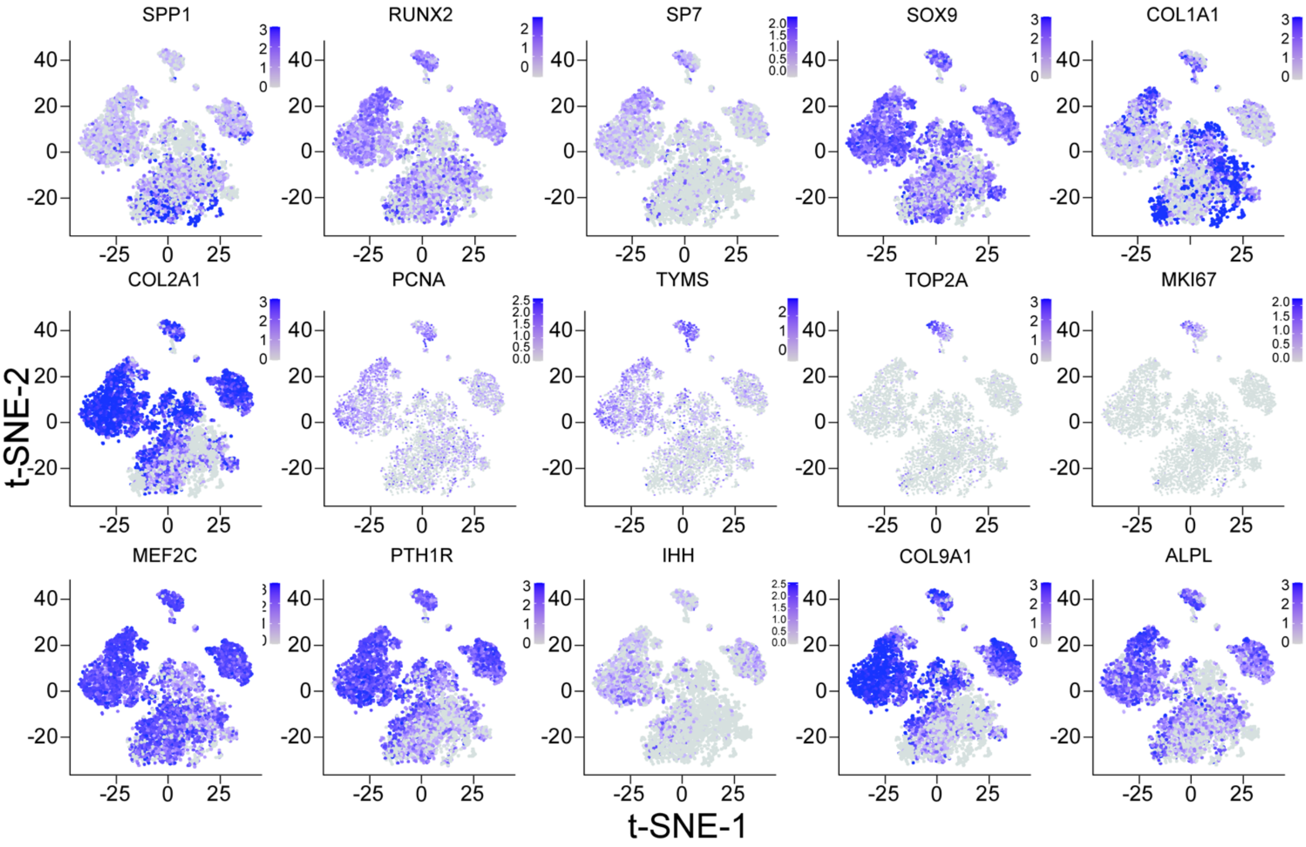

Supplementary Fig. 7. Subcluster diversity of chondroblastic OS cells. tSNE projection colored by log-normalized gene expression levels of 15 signature genes specific for each of the 4 chondroblastic subclusters.

Supplementary Figure 8

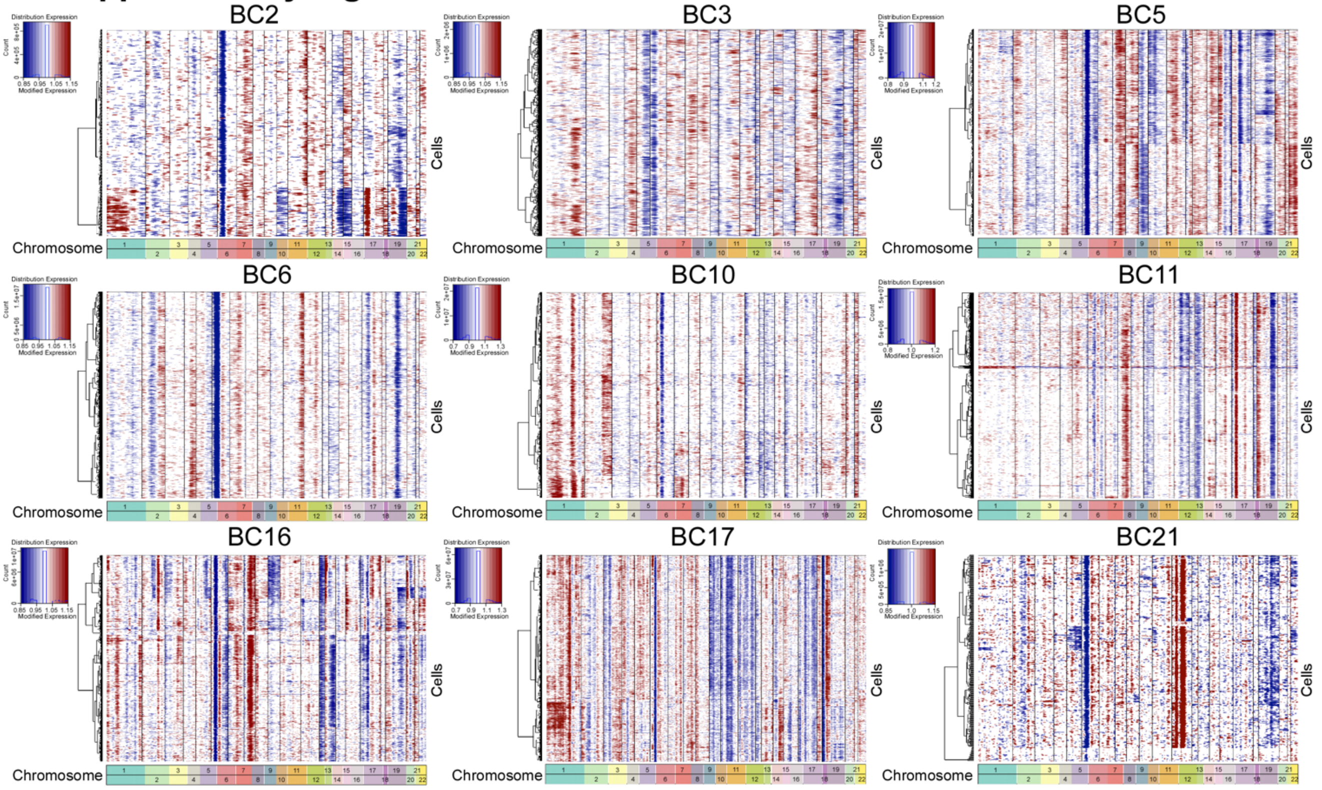

Supplementary Fig. 8. Copy-number variation analysis for 9 OS samples. The heatmaps showing the CNV profiles for the other 9 OS samples, to combine with the CNVs for 2 samples (BC20 and BC22) provided in Fig. 3. The CNVs were annotated by the hierarchical clustering to sign the 22 chromosomes according to the inferCNV analysis from each gene expression pattern. The bar showed the scaled modified CNV score of the chromosome of each single cell.

Supplementary Figure 9

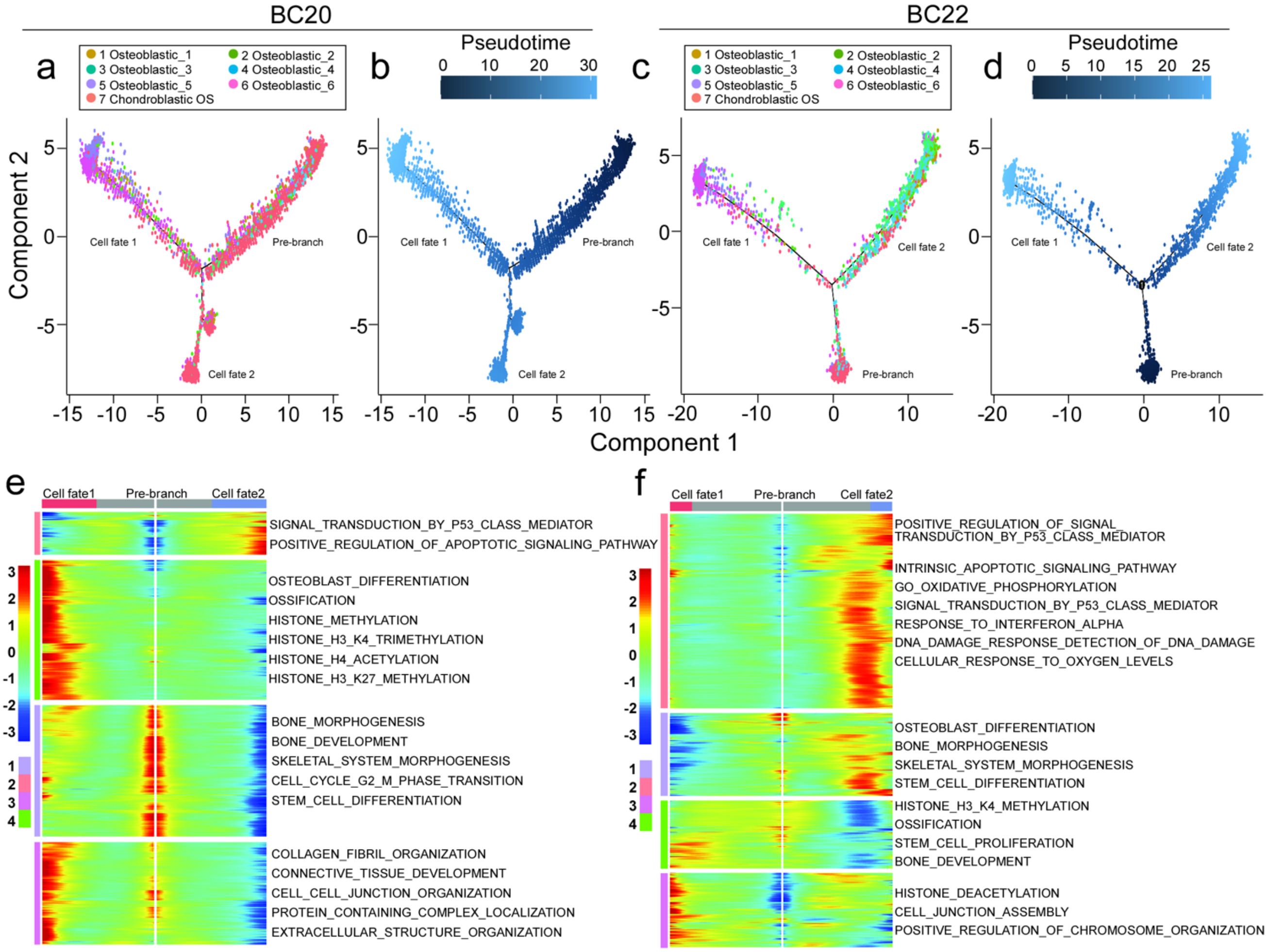

Supplementary Fig. 9. The trajectory analysis for the chondroblastic OS cells by Monocle 2 algorithm. a-d, The ordering of malignant subclusters along pseudotime is showed in a two-dimensional state space for lesion BC20 (**a, b**) and BC22 (**c, d**). **a, c** showing the distribution of the 7 subclusters in BC20 (**a**) and BC22 (**c**), respectively; (**b**) and (**d**) showing the pseudotime course of the single cells. Each dot indicates a single cell. **e, f** The pseudotime heatmap (in row) of BC20 (**e**) and BC22 (**f**) displayed the differentially expressed genes between the branches according to the BEAM analysis. The indicated cell fates in (**e**) and (**f**) were equivalent to (**a**) and (**c**), respectively.

Supplementary Figure 10

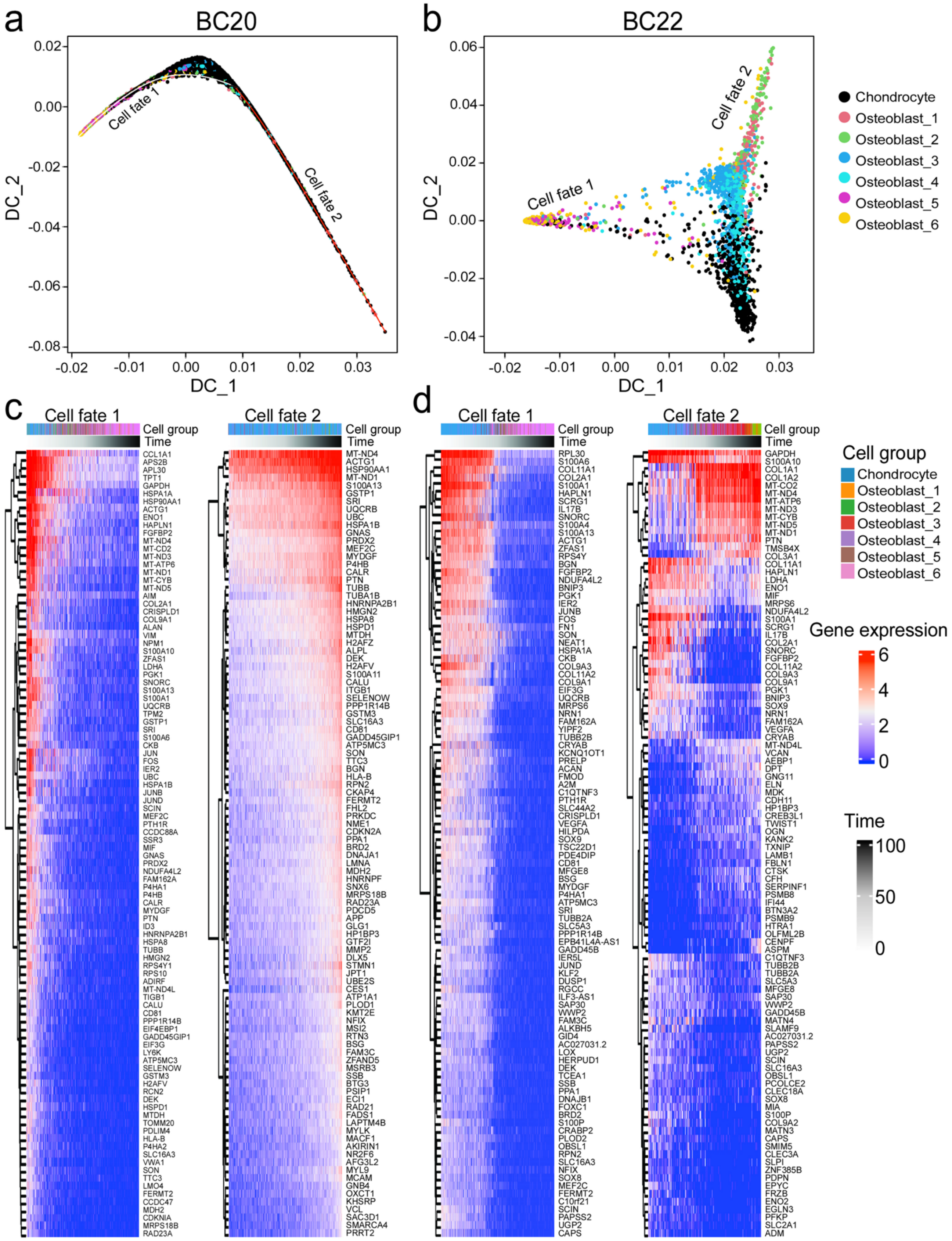

Supplementary Fig. 10. The trajectory for the chondroblastic OS cells inferred by slingshot algorithm. a, b The bifurcation cellular trajectory inferred by slingshot algorithm in patients BC20 (**a**) and BC22 (**b**). The dot color indicates the cellular subgroup identified by the t-SNE analysis. **c** Heatmap of the top 100 genes that were differentially expressed along the pseudotime in each cell fate branch as indicated in patient BC20. **d** heatmap of the top 100 genes that were differentially expressed along the pseudotime in each cell fate branch as indicated in patient BC22.

Supplementary Figure 11

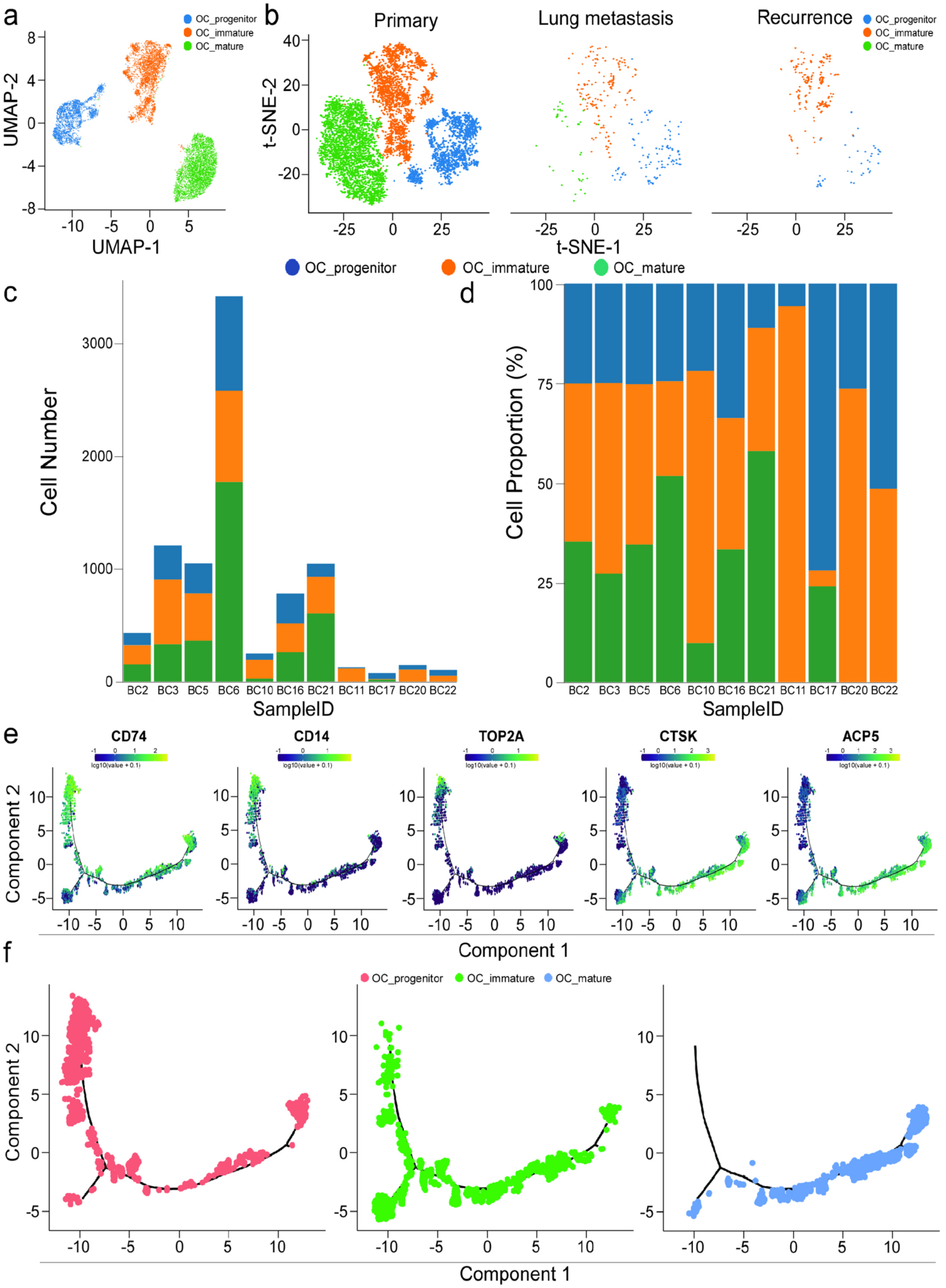

Supplementary Fig. 11. The differentiation and development of osteoclasts (OCs) in OS tissues. **a** The UMAP plot showing the 3 OC subclusters, color-coded by cell types. **b** The t-SNE plot showing the 3 OC subclusters from the primary (n = 7), lung metastasis (n = 2) and recurrent (n = 2) lesions, color-coded by cell types. **c, d** The bar chart showing the cell number (c) and proportion (d) of 3 OC subclusters in each of the 11 OS samples. **e** The expression levels of the genes *CD74, CD14, TOP2A, CTSK* and *ACP5* along with the pseudotime course of OC differentiation as determined by the Monocle 2 trajectory analysis. Color key from blue to green indicates log10 (gene expression count value + 0.1). **f** The distribution of the 3 OC subclusters identified by t-SNE analysis in the differentiation trajectory as suggested by the Monocle 2 algorithm analysis. The cell number (**c**) or proportion (**d**) values of the osteoclast subclusters in each OS patient are provided in the Source Data file.

Supplementary Figure 12

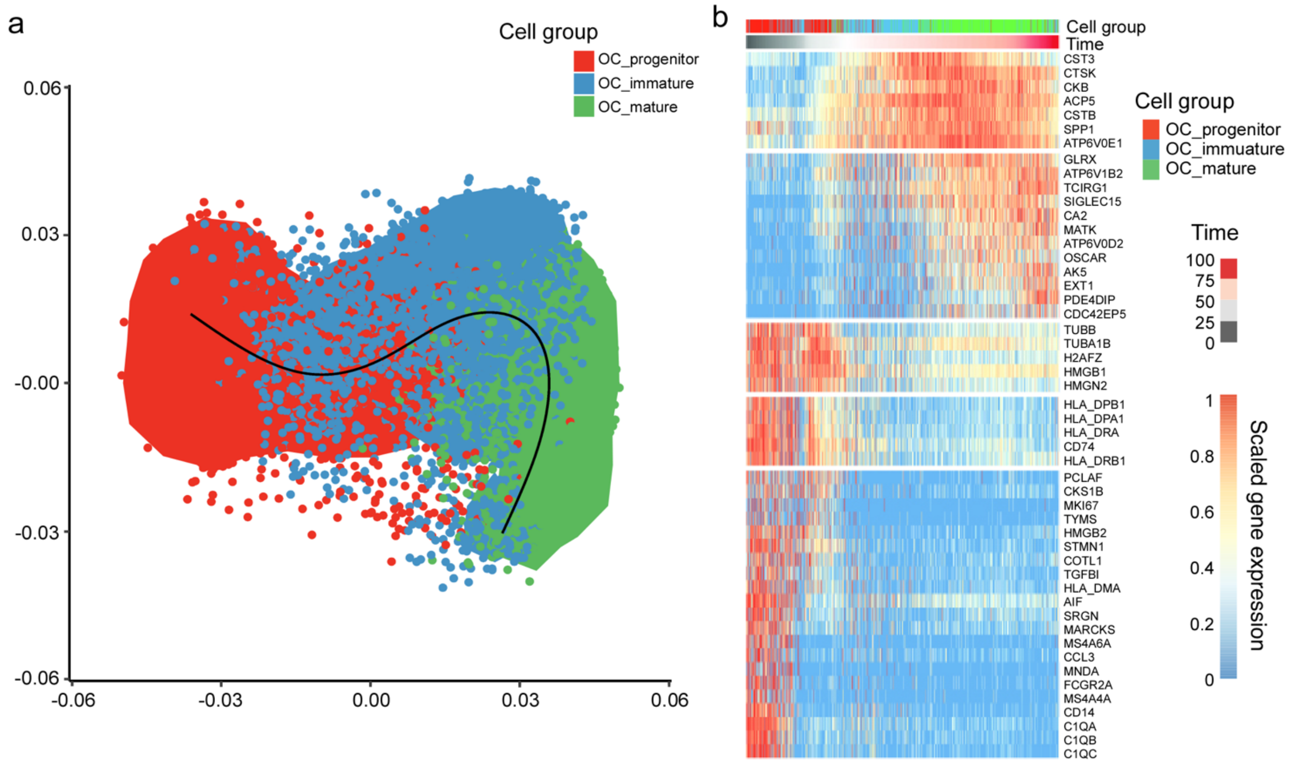

Supplementary Fig. 12. Trajectory analysis of osteoclasts differentiation using the SCORPIUS algorithm. a The trajectory plot of the osteoclasts differentiation inferred by the SCRORPIUS algorithm. The cell types defined by the t-SNE analysis were color-coded. **b** Heatmap of the gene expression profiling of the top 50 differently expressed genes (in rows) along the pseudotime trajectory of osteoclast maturation as indicated. These genes were grouped into five clusters based on their expression pattern. Color key from blue to red indicates the scaled gene expression levels of the osteoclasts.

Supplementary Figure 13

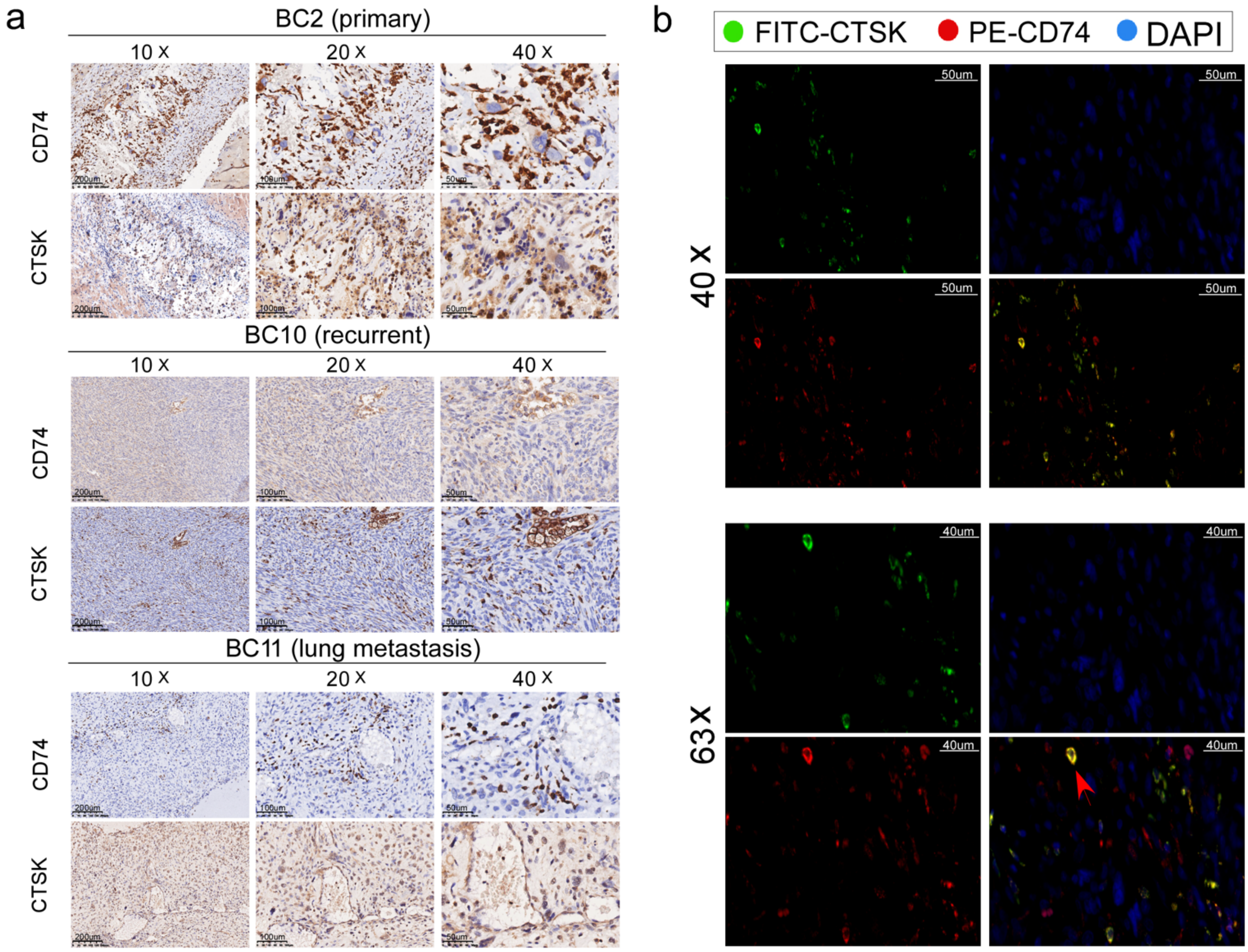

Supplementary Fig. 13. The morphological imaging analysis for OCs in OS tissues. a Representative IHC staining of the CD74 and CTSK on formalin-fixed and paraffin-embedded slides from primary (BC2; total n = 7 samples), recurrent (BC10; total n = 2 samples) and lung metastatic (BC11; total n = 2 samples) OS lesions. All replicates showed similar results. The scale bar is 50 (400 ×), 100 (200 ×) and 200 μM (100 ×) respectively. **b** The immunofluorescence staining of CTSK and CD74 in the OS lesions (total sample size n = 11). Similar results were obtained from the replicates. The red arrow indicates the co-localization of CD74 and CTSK in the OC cells. The scale bar is 50 μM (400 ×) and 40 μM (630 ×), respectively.

Supplementary Figure 14

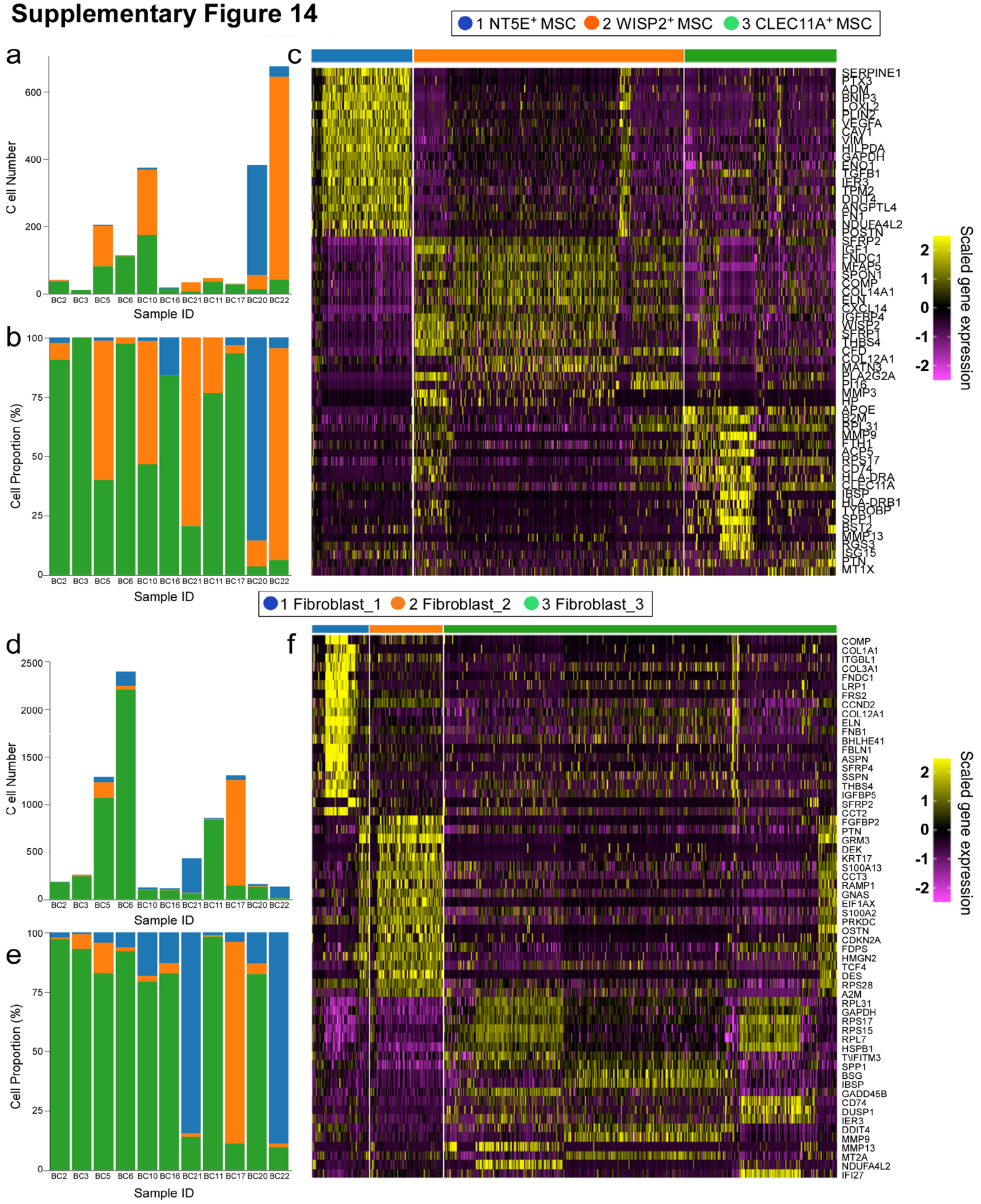

Supplementary Fig. 14. Cell subclusters of MSCs and CAFs in OS tissues. a, b The bar chart showing the cell number (**a**) and proportion (**b**) of the 3 subclusters of MSCs in each of the 11 individual samples. **c** Heatmap for the top 20 genes differentially expressed genes among the 3 MSC subclusters. The gene expression values are scaled by mean-centering, and transformed to a scale of -2 to 2. **d, e** The bar chart showing the cell number (**d**) and proportion (**e**) of 3 CAF subclusters in each of the 11 individual samples. **f** Heatmap for the top 20 differentially expressed genes among the 3 CAF subclusters. The gene expression values are scaled by mean-centering, and transformed to a scale of -2 to 2. The values of the cellular number (**a, d**) and number (**b, e**) are provided in Source Data file.

Supplementary Figure 15

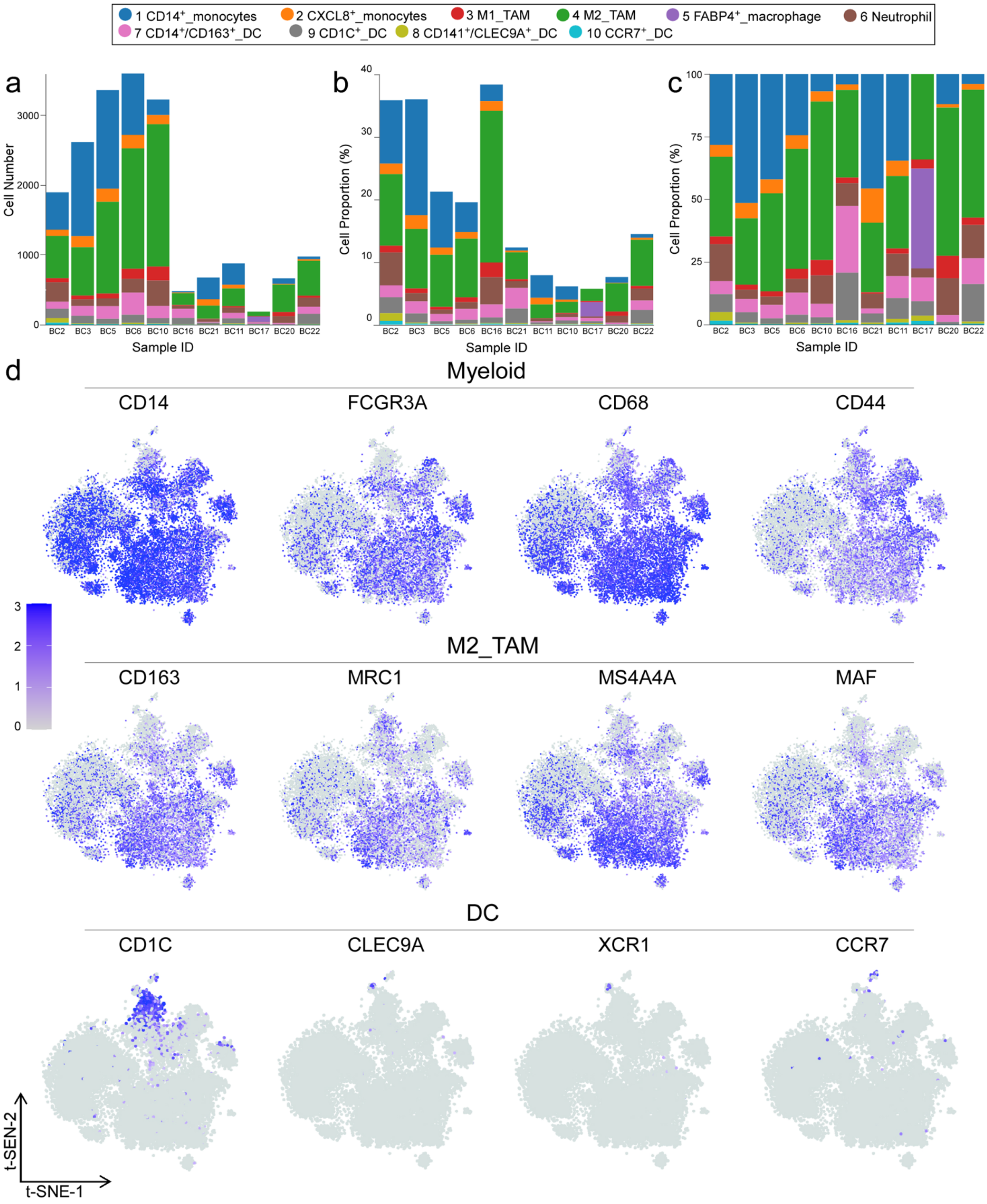

Supplementary Fig. 15. Diversity of myeloid cell subclusters in OS TME. a The bar chart showing the cell number of 10 subclusters of myeloid cells in each of the 11 OS lesions. **b** The bar chart showing the proportion of the 10 subclusters versus total captured single cells in the 11 OS samples. **c** The bar-graph presents the proportion of the 10 myeloid cell subclusters across the samples. **d** The t-SNE feature plot (from grey to blue) displays the normalized signature gene expression levels (log1p transformed) of each subclusters as indicated. The values of the cellular proportion (**b, c**) and number (**a**) are provided in Source Data file.

Supplementary Figure 16

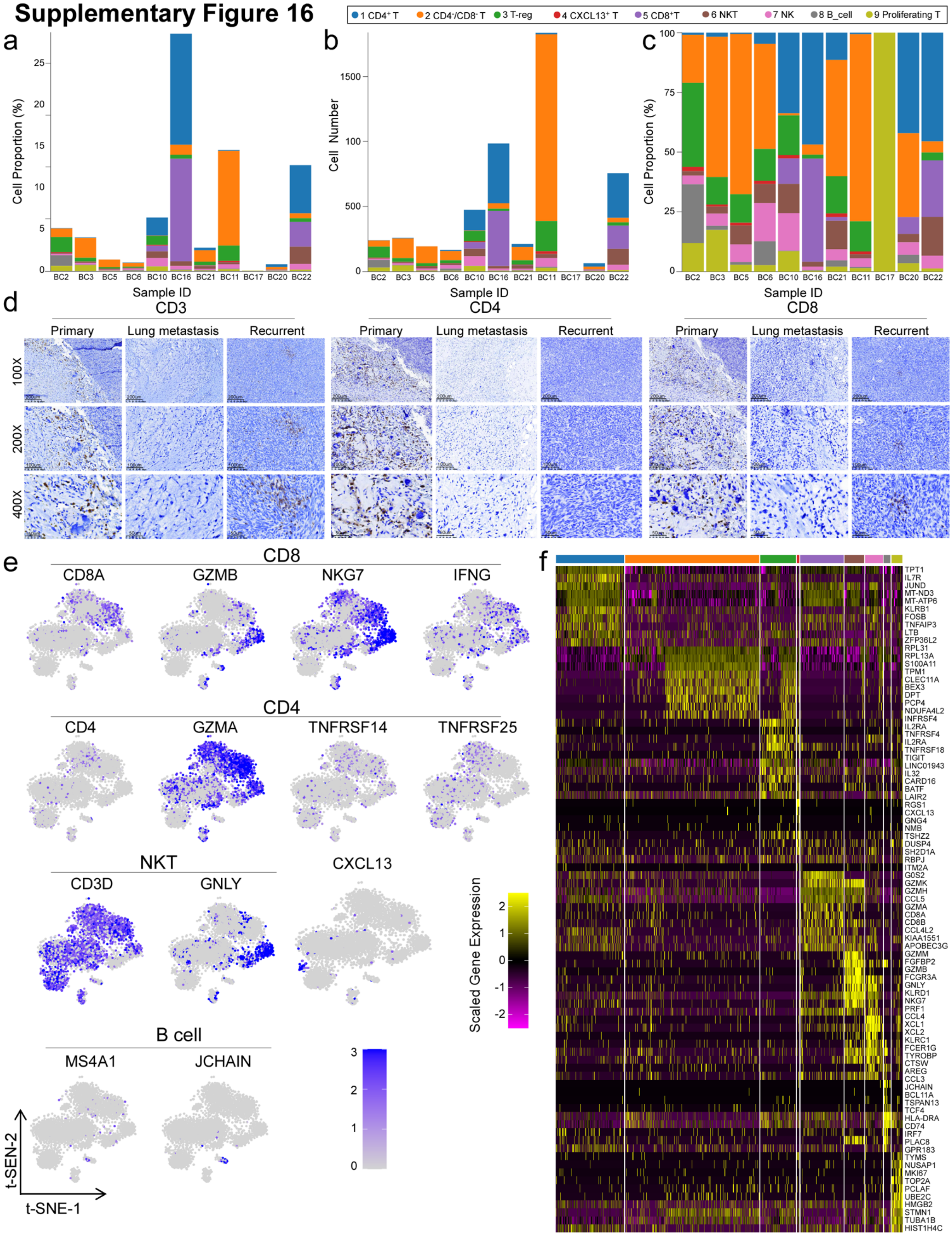

Supplementary Fig. 16. Analysis of TIL subclusters in OS TME. a The bar chart showing the percentage of the 9 TIL subclusters versus the total captured single cells in 11 OS samples. **b** The bar chart showing the cell number of 9 subclusters of TIL cells in each of the 11 OS lesions. **c** The bar-graph presents the proportion of the 9 TIL subclusters across the 11 OS lesions. **d** IHC staining of CD3, CD4 and CD8 expression levels on formalin-fixed and paraffin-embedded slides from OS lesions as indicated. Primary, n = 7 samples; Lung metastasis, n = 2 samples; Recurrent, n = 2 samples. All replicates showed similar results. The scale bar is 50 μM (400 ×), 100 μM (200 ×) and 200 μM (100 ×), respectively. (**e**) The t-SNE feature plot displays the signature gene expression levels in each cellular subcluster. The color bar (from grey to blue) indicates the normalized gene expression levels (log1p transformed). (**f**) Heatmap for the top 10 differentially expressed genes (in rows) among the 9 subclusters. The bar on the heatmap plot indicates distinct cellular subclusters as in (**a**, **b**, **c**). The gene expression values are scaled by mean-centering, and transformed to a scale of -2 to 2. The values of the cellular proportion (**a, c**) and number (**b**) are provided in Source Data file.

Supplementary Figure 17

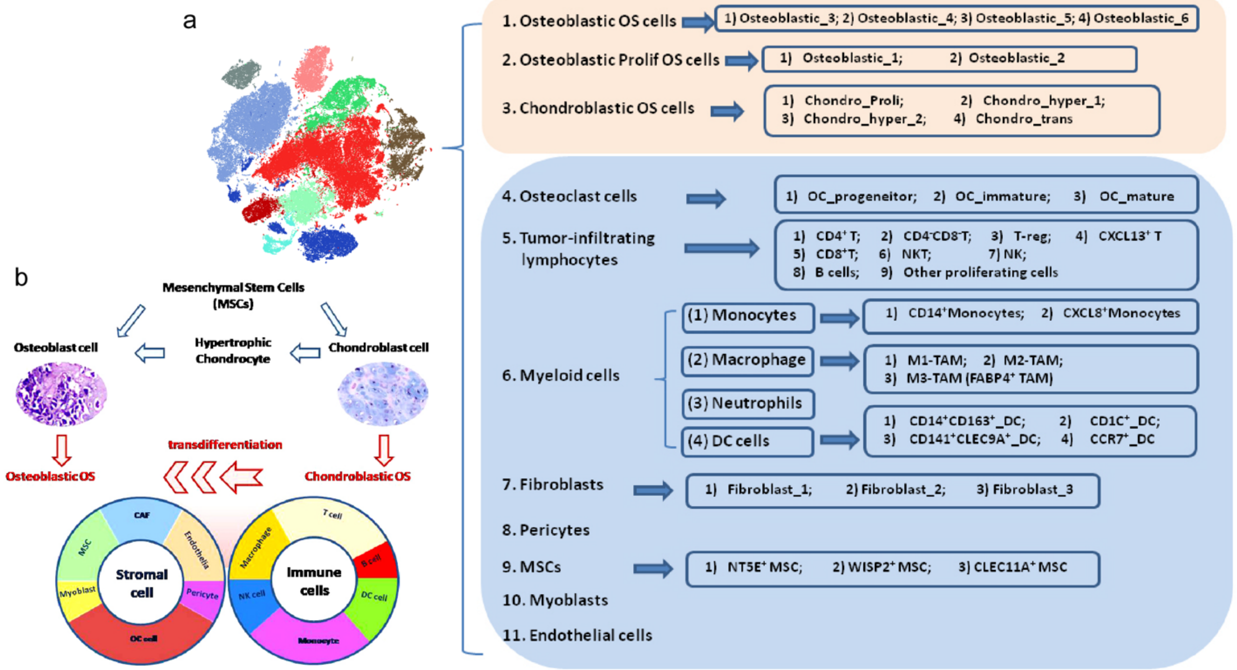

Supplementary Fig. 17. Overview of the identified cellular subclusters in scRNA-seq data of the OS lesions. a A summary of the cellular clusters and the subclusters of the 11 main cell types identified in OS lesions. **b** A schematic diagram displayed the malignant OS transdifferentiation cells and tumor microenvironment components.

Supplementary Table 1. Clinical characteristics of the osteosarcoma patients.

Sample	Pathological type	Type	Location	Preoperative chemotherapy	Size (cm)	Necrosis rate	Ki67+
BC2	Conventional	Primary	Femur	4 times (MTX, AP, IFO, MTX)	5.5*5*3	< 90%	50%
BC3	Conventional	Primary	Tibia	6 times (MTX, AP, MTX, AP, MTX, MTX)	8*6*6	< 90%	70%
BC5	Conventional	Primary	Femur	3 times (MTX, IFO, AP)	8*7.5*6	≥ 90%	80%
BC6	Conventional	Primary	Ulna	3 times (MTX, IFO, AP)	7*7*4	≥ 90%	15%
BC10	Conventional	Metastasis (Lung)	Femur	2 times (GT, GT)	3.5*3*2	< 90%	8%
BC11	Conventional	Recurrent	Femur	3 times (GT, GT, GT)	20*11*10	< 90%	30%
BC16	Conventional	Primary	Tibia	4 times (IFO, AP, MTX, MTX)	6*4*2.5	< 90%	40%
BC17	Chondroblastic	Metastasis (Lung)	Tibia	3 times (GT, GT, GT)	2*2*1.5	< 90%	40%
BC20	Chondroblastic	Recurrent	Femur	4 times (AP, IE, AP, IE)	10*8*5	< 90%	50%
BC21	Intraosseous osteosarcoma	Primary	Femur	4 times (MTX, AP, IFO, AP)	5*4*1	< 90%	10%
BC22	Chondroblastic	Primary	Femur	4 times (MTX, AP, IFO, MTX)	18*15*12	< 90%	20%

Abbreviations: MTX, Methotrexate; AP, Doxorubicin + Cisplatin; IE, IFO + VP-16; IFO, Ifosfamide; GT, Gemcitabine + Docetaxel; VP-16, Etoposide.

Supplementary Table 2. The canonical markers for the 11 cell clusters in osteosarcoma tissues.

Cell cluster	Marker genes	Supplementary Reference
Osteoblastic cells	Runx2, Col1a1, Cdh11, Ibsp	[1-3]
Chondroblastic cells	Sox9, Acan, Pth1r	[1, 4]
Osteoclasts	ACP5, Ctsk, Mmp9	[5]
Myeloid cells	Cd74, Cd14, Fcgr3a	[6, 7]
T cells	CD3, IL7R, CD8a, CD4, Nkg7	[8]
NK cells	Nkg7, Gnly	[7]
NKT cells	Nkg7, Gnly, Cd3	[9]
DC cells	Cd1c, Fcer1a, Clec9a, Ccr7, Cd14, Cd163	[7, 8]
Fibroblasts	Dcn, Col1a1	[8, 10]
Pericytes	Rgs5, Acta2	[8]
Mesenchymal stem cells	Mme, Thy1, Cxcl12, Sfrp2	[1]
Endothelial cells	Pecam1, Vwf	[8]
Myoblasts	Myl1, Mylpf	[11]
B cells	Ms4a1, Cd19, Jchain	[8, 12]
Proliferating cells	Mki67, Top2a, Pcna	[8, 13]

This table is generated from the public literature cited.

Supplementary References

1. Baryawno, N., et al., A Cellular Taxonomy of the Bone Marrow Stroma in Homeostasis and Leukemia. Cell, 2019. **177**(7): p. 1915-1932 e16.
2. Alimperti, S. and S.T. Andreadis, CDH2 and CDH11 act as regulators of stem cell fate decisions. Stem Cell Res, 2015. **14**(3): p. 270-82.
3. Kalajzic, I., et al., Expression profile of osteoblast lineage at defined stages of differentiation. J Biol Chem, 2005. **280**(26): p. 24618-26.
4. Vortkamp, A., et al., Regulation of rate of cartilage differentiation by Indian hedgehog and PTH-related protein. Science, 1996. **273**(5275): p. 613-22.
5. Aliprantis, A.O., et al., NFATc1 in mice represses osteoprotegerin during osteoclastogenesis and dissociates systemic osteopenia from inflammation in cherubism. J Clin Invest, 2008. **118**(11): p. 3775-89.
6. Guo, X., et al., Global characterization of T cells in non-small-cell lung cancer by single-cell sequencing. Nat Med, 2018. **24**(7): p. 978-985.
7. Zhang, Q., et al., Landscape and Dynamics of Single Immune Cells in Hepatocellular Carcinoma. Cell, 2019. **179**(4): p. 829-845 e20.
8. Kim, N., et al., Single-cell RNA sequencing demonstrates the molecular and cellular reprogramming of metastatic lung adenocarcinoma. Nat Commun, 2020. **11**(1): p. 2285.
9. Godfrey, D.I., et al., NKT cells: what's in a name? Nat Rev Immunol, 2004. **4**(3): p. 231-7.

10. Elyada, E., et al., Cross-Species Single-Cell Analysis of Pancreatic Ductal Adenocarcinoma Reveals Antigen-Presenting Cancer-Associated Fibroblasts. Cancer Discov, 2019. **9**(8): p. 1102-1123.
11. Niro, C., et al., Six1 and Six4 gene expression is necessary to activate the fast-type muscle gene program in the mouse primary myotome. Dev Biol, 2010. **338**(2): p. 168-82.
12. MacParland, S.A., et al., Single cell RNA sequencing of human liver reveals distinct intrahepatic macrophage populations. Nat Commun, 2018. **9**(1): p. 4383.
13. Patel, A.P., et al., Single-cell RNA-seq highlights intratumoral heterogeneity in primary glioblastoma. Science, 2014. **344**(6190): p. 1396-401.
