# Supplementary material for: Single-cell RNA landscape of intratumoral heterogeneity and immunosuppressive microenvironment in advanced osteosarcoma
Source: Nat Commun. 2020 Dec 10;11:6322. doi: 10.1038/s41467-020-20059-6 (PMC7730477; doi:10.1038/s41467-020-20059-6)
Supplement: Supplementary file 3 — Description of Additional Supplementary Files [file 41467_2020_20059_MOESM3_ESM.pdf]

Description of Additional Supplementary Files

File name: Supplementary Data 1

Description: The top 20 differentially expressed genes of the osteoblastic OS subclusters. The "pct.1" and "pct.2" refer to the percent of cells (%) that are positive for the indicated genes among all cells in the subjected subcluster and the other subclusters beyond, respectively. The "avg_logFC" refers to the log fold-change of the average gene expression levels between the subjected cell subcluster and the other subclusters, while the "p_val" refers to the Wilcoxon Rank Sum test results (two-sided test) and "p_val_adj" is the corresponding Bonferroni-corrected "p_value".

File name: Supplementary Data 2

Description: The top 20 differentially expressed genes of the chondroblastic OS subclusters. The "pct.1" and "pct.2" refer to the percent of cells (%) that are positive for the indicated genes among all cells in the subjected subcluster and the other subclusters beyond, respectively. The "avg_logFC" refers to the log fold-change of the average gene expression levels between the subjected cell subcluster and the other subclusters, while the "p_val" refers to the Wilcoxon Rank Sum test results (two-sided test) and "p_val_adj" is the corresponding Bonferroni-corrected "p_value".

File name: Supplementary Data 3

Description: The top 20 differentially expressed genes of the osteoclast subclusters. The "pct.1" and "pct.2" refer to the percent of cells (%) that are positive for the indicated genes among all cells in the subjected subcluster and the other subclusters beyond, respectively. The "avg_logFC" refers to the log fold-change of the average gene expression levels between the subjected cell subcluster and the other subclusters, while the "p_val" refers to the

Wilcoxon Rank Sum test results (two-sided test) and "p_val_adj" is the corresponding Bonferroni-corrected "p_value".

File name: Supplementary Data 4

Description: The top 20 differentially expressed genes of the MSC subclusters. The "pct.1" and "pct.2" refer to the percent of cells (%) that are positive for the indicated genes among all cells in the subjected subcluster and the other subclusters beyond, respectively. The "avg_logFC" refers to the log fold-change of the average gene expression levels between the subjected cell subcluster and the other subclusters, while the "p_val" refers to the Wilcoxon Rank Sum test results (two-sided test) and "p_val_adj" is the corresponding Bonferroni-corrected "p_value".

File name: Supplementary Data 5

Description: The top 20 differentially expressed genes of the fibroblast subclusters. The "pct.1" and "pct.2" refer to the percent of cells (%) that are positive for the indicated genes among all cells in the subjected subcluster and the other subclusters beyond, respectively. The "avg_logFC" refers to the log fold-change of the average gene expression levels between the subjected cell subcluster and the other subclusters, while the "p_val" refers to the Wilcoxon Rank Sum test results (two-sided test) and "p_val_adj" is the corresponding Bonferroni-corrected "p_value".

File name: Supplementary Data 6

Description: The top 20 differentially expressed genes of the myeloid cell subclusters. The "pct.1" and "pct.2" refer to the percent of cells (%) that are positive for the indicated genes among all cells in the subjected subcluster and the other subclusters beyond, respectively.

The “avg_logFC” refers to the log fold-change of the average gene expression levels between the subjected cell subcluster and the other subclusters, while the “p_val” refers to the Wilcoxon Rank Sum test results (two-sided test) and “p_val_adj” is the corresponding Bonferroni-corrected “p_value”.

File name: Supplementary Data 7

Description: The top 20 differentially expressed genes of the T&NK subclusters. The “pct.1” and “pct.2” refer to the percent of cells (%) that are positive for the indicated genes among all cells in the subjected subcluster and the other subclusters beyond, respectively. The “avg_logFC” refers to the log fold-change of the average gene expression levels between the subjected cell subcluster and the other subclusters, while the “p_val” refers to the Wilcoxon Rank Sum test results (two-sided test) and “p_val_adj” is the corresponding Bonferroni-corrected “p_value”.
