# Supplementary material for: Single-cell RNA landscape of intratumoral heterogeneity and immunosuppressive microenvironment in advanced osteosarcoma
Source: Nat Commun. 2020 Dec 10;11:6322. doi: 10.1038/s41467-020-20059-6 (PMC7730477; doi:10.1038/s41467-020-20059-6)
Supplement: Supplementary file 11 — Reporting Summary [file 41467_2020_20059_MOESM11_ESM.pdf]

nature research

Corresponding author(s): Haiyan Hu, Peizhan Chen, Xinghua Pan, and Yang Yao.

Last updated by author(s): Oct 15, 2020

Reporting Summary

Nature Research wishes to improve the reproducibility of the work that we publish. This form provides structure for consistency and transparency in reporting. For further information on Nature Research policies, see our Editorial Policies and the Editorial Policy Checklist.

Statistics

For all statistical analyses, confirm that the following items are present in the figure legend, table legend, main text, or Methods section.

n/a	Confirmed	
☐	☒	The exact sample size (n) for each experimental group/condition, given as a discrete number and unit of measurement
☐	☒	A statement on whether measurements were taken from distinct samples or whether the same sample was measured repeatedly
☐	☒	The statistical test(s) used AND whether they are one- or two-sided<br>*Only common tests should be described solely by name; describe more complex techniques in the Methods section.*
☒	☐	A description of all covariates tested
☐	☒	A description of any assumptions or corrections, such as tests of normality and adjustment for multiple comparisons
☐	☒	A full description of the statistical parameters including central tendency (e.g. means) or other basic estimates (e.g. regression coefficient) AND variation (e.g. standard deviation) or associated estimates of uncertainty (e.g. confidence intervals)
☐	☒	For null hypothesis testing, the test statistic (e.g. F, t, r) with confidence intervals, effect sizes, degrees of freedom and P value noted<br>*Give P values as exact values whenever suitable.*
☒	☐	For Bayesian analysis, information on the choice of priors and Markov chain Monte Carlo settings
☐	☒	For hierarchical and complex designs, identification of the appropriate level for tests and full reporting of outcomes
☐	☒	Estimates of effect sizes (e.g. Cohen's d, Pearson's r), indicating how they were calculated

Our web collection on statistics for biologists contains articles on many of the points above.

Software and code

Policy information about availability of computer code

Data collection	No specific code and software were used for data collection. All scRNA-seq in the study were generated by the research groups.
Data analysis	The sequencing data from 10x Genomics platform were processed to generate gene expression profiles using the standard internal pipeline based on the Cell Ranger toolkit (version 2.1.1). All single cell data analysis were performed with the R software (v3.6.1) and related packages including Seurat (v3.1.5), Monocle 2 (v2.14.0), inferCNV (v1.2.2), DoubletFinder (v2.0.2), singleseqgsea (v 0.1.2), clusterProfiler (v3.14.3), GSVA (v1.34.0) and pheatmap (v1.0.12). Data from different patients were integrated with the Louvain algorithm implemented in R package Harmony (v1.0). The intra-tumor evolutionary tree of the patients were analyzed and plotted with UPhyloplot2 algorithm (https://github.com/harbourlab/UPhyloplot2). General continuous variables are expressed as mean ± standard deviation (SD). The significance of differences was determined using the paired or unpaired Student's t-test as indicated, and differences with two-sided p < 0.05 were considered as statistically significant.<br>The code generated for the analysis of the scRNA-seq data were provided at: https://github.com/ChenPeizhan/osteosarcoma.

For manuscripts utilizing custom algorithms or software that are central to the research but not yet described in published literature, software must be made available to editors and reviewers. We strongly encourage code deposition in a community repository (e.g. GitHub). See the Nature Research guidelines for submitting code & software for further information.

Data

Policy information about availability of data

All manuscripts must include a data availability statement. This statement should provide the following information, where applicable:

- Accession codes, unique identifiers, or web links for publicly available datasets
- A list of figures that have associated raw data
- A description of any restrictions on data availability

Single-cell expression data can also be accessed from the NCBI Gene Expression Omnibus database (accession code GSE152048 [https://www.ncbi.nlm.nih.gov/geo/query/acc.cgi?acc=GSE152048]). The transcriptional factors were retrieved from the differentially expressed genes based on the AnimalTFDB (v3.0) database (http://bioinfo.life.hust.edu.cn/AnimalTFDB/#!/). The activation of the gene sets were retrieved from the MSigDB database (https://www.gsea-msigdb.org/gsea/msigdb). The source data underlying Figs1e, 2d, 2e, 2g, 2h, 3a, 5b, 5e, 6d, 6e, 6g, 7f and Supplementary Figs 5a, 6b, 6c, 6g, 6h, 11c, 11d, 14a, 14b,14d, 14e, 15a, 15b, 15c, 16a, 16b, 16c are provided as a Source Data file. All the other data supporting the findings of this study are available within the article and its supplementary information files without any restrictions or from the corresponding author upon reasonable request.

Field-specific reporting

Please select the one below that is the best fit for your research. If you are not sure, read the appropriate sections before making your selection.

- [x] Life sciences
- [ ] Behavioural & social sciences
- [ ] Ecological, evolutionary & environmental sciences

For a reference copy of the document with all sections, see nature.com/documents/nr-reporting-summary-flat.pdf

Life sciences study design

All studies must disclose on these points even when the disclosure is negative.

Sample size	11 patients (8 primary osteoblastic osteosarcoma patients and 3 primary chondroblastic osteosarcoma patients) were randomly recruited for the 10xGenomics scRNA-seq analysis, and no power calculation was done as the current study was an exploratory project. These patients represented the most commonly identified osteosarcoma types in clinic. The circulating T cells from 4 independent donors (2 with higher tumor TIGIT+ T cells infiltration and 2 with lower tumor TIGIT+ T cells infiltration) were randomly collected to test the cellular cytotoxic activities for T cells under the blockage of TIGIT.
Data exclusions	We applied pre-established quality assessment and data excluded criteria on raw gene matrix data for the single cells: (1) cells were assigned as the potential doublets based on the DoubletFinder algorithm; (2) cells with detected genes less than 300, and (3) cells with the mitochondrial genes over 10% of the total counts.
Replication	As the expected cellular numbers were collected from the scRNA-seq data based on the 10x Genomics platform, the scRNA-seq for each patients was not repeatedly performed. The cellular cytotoxicity assays were repeated for 3 times and similar results were obtained.
Randomization	As the patients were not deemed to receive any specific intervention treatments by groups, the patients were not randomly assigned into groups. All the patients in the current study were recruited randomly.
Blinding	As the study was not an intervention study, the blinding is not necessary for the current this study. The aim of the current study was to evaluate the tumor cellular heterogeneity and tumor micro-environment of the osteosarcoma leisions.

Reporting for specific materials, systems and methods

We require information from authors about some types of materials, experimental systems and methods used in many studies. Here, indicate whether each material, system or method listed is relevant to your study. If you are not sure if a list item applies to your research, read the appropriate section before selecting a response.

Materials & experimental systems

n/a	Involved in the study	
☐	☒	Antibodies
☐	☒	Eukaryotic cell lines
☒	☐	Palaeontology and archaeology
☒	☐	Animals and other organisms
☐	☒	Human research participants
☐	☒	Clinical data
☒	☐	Dual use research of concern

Methods

n/a	Involved in the study	
☒	☐	ChIP-seq
☒	☐	Flow cytometry
☒	☐	MRI-based neuroimaging

Antibodies

Antibodies used	The antibodies used in the current study are listed below:<br>TIGIT (cat # ab243903, Abcam, USA)<br>CD3 (cat # ab5690, Abcam)<br>CD4 (cat # ab213215, Abcam)<br>CD8 (cat # ab17147, Abcam)<br>CD74 (cat # ab22603, Abcam)<br>CTSK (cat # ab37259, Abcam)<br>anti-CD3 antibody (clone OKT3; cat # 317302; Biolegend)<br>anti-TIGIT antibody (clone A15153G; cat # 372702; Biolegend)<br>donkey anti-rabbit Alexa Fluor 488 (# A21202, 1:1000; Molecular Probes)<br>goat anti-mouse Alexa Fluor 514 (#A31555, 1:1000; Molecular Probes)
Validation	All antibodies used in this study are commercially available and validated by manufacturers. Detailed information of each commercially available antibody including their applications, specificity, protocols and properties etc. were provided by the manufacturers' websites (https://www.abcam.com, https://www.biolegend.com, and https://www.thermofisher.com/us/en/home/brands/molecular-probes.html).

Eukaryotic cell lines

Policy information about cell lines

Cell line source(s)	The osteosarcoma cell lines 143B and U2OS cells were provided by the Cell Bank of China Academy of Sciences (Shanghai, China).
Authentication	The cell lines were authenticated at the cell bank of China Academy of Sciences with the DNA fingerprinting methods using primer sets DXS52, Apo-B, MD17S5 and D2S44.
Mycoplasma contamination	Cell lines used in this study tested negative for mycoplasma contamination.
Commonly misidentified lines<br>(See ICLAC register)	No commonly misidentified lines were used in the study.

Human research participants

Policy information about studies involving human research participants

Population characteristics	Of the 11 patients, 5 were male and 6 were female, with the age range from 11 to 38 years old. Eight were diagnosed with the primary osteoblastic sarcoma and three were diagnosed with primary chondroblastic sarcoma. Primary tumor tissues were collected from 7 patients who had received traditional first-line adjuvant and neo-adjuvant chemotherapy composed of a cocktail of four drugs (including doxorubicin, cisplatin, methotrexate and ifosfamide), as well as surgical therapy. The lung metastasis tumor tissues were collected from 2 patients received lung lobectomy treatments and another 2 tumor tissues were collected from the recurrent tumor tissues who had received gemcitabine combined with docetaxel (GT) treatments or sequential treatments including doxorubicin/cisplatin and ifosfamide/VP-16. Detailed information of the individual patients were provided in Supplementary Table 1 of the manuscript. Each patient provided written consent.
Recruitment	Eleven patients hospitalized from October 2017 to April 2019 in Shanghai Sixth People's Hospital were prospectively enrolled in the study. All patients had received the surgery treatments in our clinical department were randomly recruited. Those patients that were not eligible for the surgery treatment or were unwilling to participate in the research were excluded in the current study.
Ethics oversight	The study was approved by the Shanghai Sixth People's Hospital Ethics Committee.

Note that full information on the approval of the study protocol must also be provided in the manuscript.

Clinical data

Policy information about clinical studies

All manuscripts should comply with the ICMJEguidelines for publication of clinical research and a completedCONSORT checklist must be included with all submissions.

Clinical trial registration	N/A
Study protocol	N/A
Data collection	N/A
Outcomes	N/A
