# Supplementary material for: Single-cell RNA landscape of intratumoral heterogeneity and immunosuppressive microenvironment in advanced osteosarcoma
Source: Nat Commun. 2020 Dec 10;11:6322. doi: 10.1038/s41467-020-20059-6 (PMC7730477; doi:10.1038/s41467-020-20059-6)
Supplement: Supplementary file 2 — Peer Review File [file 41467_2020_20059_MOESM2_ESM.pdf]

Reviewers' comments:

Reviewer #1 (Remarks to the Author):

This manuscript presents a novel and worthwhile study of heterogeneity in osteosarcoma, using single cell RNA sequencing suggesting the presence of various cell types in osteosarcoma tumors. While the results are interesting several issues need to be addressed in order for the manuscript to be considered for publication. Specifically, critical methodological details are missing, and the data and results need to be re organized and presented in a way that conveys the most important biologic or clinical inferences from the data. Critical algorithms and methods are not explained in adequate detail and abbreviations are not spelled out. The contrast between the various cell types is not presented in a clear enough manner, or in a manner that relates to features of osteosarcoma biology or clinical behavior. Finally, some terms and vocabulary is not always clearly explained.

Some specific issues and examples that need to be addressed.

Perhaps a "summary figure" can be shown presenting all the subgroups and cell types and the methods used to identify them in one graph with their relative abundance as well as the main biologic themes represented and whether each subgroup corresponds to any specific biologic or clinical feature of the disease. Such a figure can be shown at the end of the Results, and may provide a more clear summary of the most important findings of the study. The currently presented detailed Figures, while containing potentially important information, do not contribute to a clear enough understanding of the main and more relevant findings of the study.

Line 76-78: "We found that the TME....suppressivity than primary tumor tissue". This meaning of this sentence is unclear.

Line 130: What is UMI?

Line 135. The Seurat program needs to be referenced with appropriate literature.

Details of how the Seurat algorithm and the t-SNE method were applied are necessary. If these details are too many to easily report, they should be listed in a Supplementary Methods file. Findings from these methods are often very sensitive to specific parameters and tuning. Without more details of how the algorithms were applied, it is difficult to assess the validity of all results. For example, how did the algorithms decide about the number of subgroups and objects in each analysis and how was it concluded that these subgroups are statistically robust?

Line 142: t-SNE needs to be spelled out.

How was copy number variation (CNV) estimated using RNAseq data? Were there genomic DNA sequencing data also obtained?

Line 169. What is the Monocle 2 program and is there a citation for this?

Line 255: The concept of trajectory analysis is unclear. What is "pseudo-time"? This analysis may also benefit from a dedicated graph showing how it is done and summarizing what it reveals.

Abbreviations such as CAFs (and all its variants such as capCAFs, myCAFs, apCAFs) need to be spelled out not just in the Abstract, but also the first time they appear in the main manuscript.

The TIGIT blockade section is confusing. What type of cells were used? The body of the manuscript reports that this was done on "OS cells", whereas Figure 7 legend states that it was done on "breast cancer cell lines" and the Figure text reports the U2OS cell line.

What was the blood that one study subject donates (line 104) used for? In the Methods section, it is stated that it was used to explore the effect of anti-TIGIT blockage but the respective paragraph in the Results section does not include any reference to blood.

The overall language (use of English) of the manuscript needs to be reviewed in detail and improved.

Reviewer #2 (Remarks to the Author):

The authors correctly claim that their study is the first to employ single-cell RNA-sequencing to study Osteosarcoma (OS). They furthermore claim to utilize this technology to explore the molecular mechanism of OS as well as the tumor microenvironment (TME). They then describe the clusters they found which are formed by the 110 745 single cell profiles as well as state that TIGIT is expressed in 11 samples and is particularly overexpressed in T-reg cells. The then isolate CD3+ T cells and block TIGIT signaling in those cells which according to the authors yields improved cytotoxicity of Cytokine induced T cells. Their approach to use single cell resolution combined with trajectory inference methods to study OS cell heterogeneity is very interesting in particular since this study provides novel insight into the transcriptional cell types in OS and may indeed be of relevance as a reference for other studies.
However, there are some shortcomings of the present study that need to be addressed. First of all, while the total number of single cell transcriptomes profiled appears reasonable, the representation of primary OS tumors (7) compared to recurrent OS tumors (2) and pulmonary metastasis (2) is much higher and it is absolutely crucial to include similar numbers of cells from each of the conditions to be able to perform reliable comparisons, in particular when aligning cells along a pseudo-time. The authors need to analyze at least the same number of patient samples for each condition (7). The authors comment on the inability to capture cancer stem cells due to their low number. An increased sample size will facilitate to confidently rule out that lack of stem cell capture is due to underrepresentation of samples or small sample size.
Seven of the selected patients were under combined chemotherapy as well as surgical therapy. Cellular heterogeneity and underlying transcriptional signatures must therefore be seen in the light of this continuous drug treatment. It would be of great interest if the authors investigated further which cell populations are particularly affected by the drugs and how combinatorial drug treatment is encoded in the transcriptome of different cell populations. In addition, gene signatures related to sample type, isolation strategy and treatment might confound cell type calling- this needs to be discussed and addressed by the authors. Furthermore, the authors need to demonstrate the degree of correlation of each cell type between the different patient samples.

The authors need to provide more detailed information in the methods how quality control was performed to exclude single cell libraries from further analysis. They nicely show representations of the main cell populations for each patient sample used. Since a fixed embedding is used for this visualization, it will be important to see a t-SNE or UMAP representation of all patient samples labeled by experiment/ patient ID/ experimental run to demonstrate the absence of batch-to-batch variation. They also need to state average sequencing depth, UMI and gene count per cell.
In Figure 1A, the myeloid cell cluster is already split up in initial t-SNE representation suggesting either sub-clusters to myeloid cluster or entirely new populations. The authors need to computationally show that after initial clustering, there is not more biological diversity in the myeloid cluster and that this cluster does not contain more distant cell types. The authors state that the TP53 pathway is markedly "disordered in subgroup 5" in Figure S2A- the use of the word "disordered" needs to be explained.
Furthermore, the authors use GO terms to infer putative signaling or functionality onto the transcriptome data. However, the GO term analysis in Figure S2A does not provide a clear distinction

between the different populations and is therefore not informative. E.g. metabolic and proliferative OS as well as bone-matrix remodeling and ossification OS greatly overlap. The separation of these populations by GO term analysis is not conclusive and needs to be substantiated. Cluster analysis as in Figure S2B allows to separate those cells however, only rather few genes actually contribute to this separation.
The exploration of trajectories is very interesting, however, the referencing to individual genes in the actual figure needs to be made clearer as well as the figure themselves: Figure 2D needs to be subdivided and genes as well as GO terms which do not contribute to further analysis, should be remove to deconvolute the figure.
Pseudo-time analysis of OC maturation nicely shows highest expression of CD74 and TOP2A in progenitor OCs. These cells subsequently serve as the origin for trajectory building. The authors need to provide more information about selecting seed cells plus gene signatures which serve as the origin of the trajectory. Furthermore, since trajectory building is not trivial and strongly depends on the trajectories to be build and can differ between available methodologies (bifurcating vs multifurcating trajectories), the author must test different available solutions and carefully evaluate the trajectory building outcome. Since many conclusions of this manuscript are founded on this type of analysis, it is absolutely crucial to ensure that the utilized algorithm reflects the underlying biological processes in the most conclusive way.
In Figure 3E, the authors describe GO terms to describe biological process as they change following OC differentiation. As opposed to earlier analyses in this manuscript, the selected GO terms are either sharply present or absent at both endpoints of the trajectory. None of the GO terms show gradual acquisition or decline along differentiation axis. The authors need to provide relevant GO terms which gradually alter over time as it now appears to be the transition between two cell stages only. Figure S4 is not mentioned in the text.
Next, the authors use Seurat to cluster myCAF and apCAF. Unfortunately, based on the clustering they present, not two but rather 7 distinct gene signatures can be identified (Figure 4C). The authors must clarify the discrepancy between the t-SNE distribution of two populations compared to the finer, but unfortunately ignored clustering by Seurat. The results of this part are as of now inconclusive.
In figure 6 C, CD8+ T-cells and CD4-/ CD8- T-cells basically do not differ in gene expression. In the t-SNE representation (6A) both cell populations might separate. Gene signatures need to be provided which actually delineate both cell populations.
The cell-based assay to assess blocking of TIGIT signaling on cytotoxicity is presented as being statistically significant. However, in Figure 7B, none of the comparisons Control vs. TIGIT inhibition shows a significant effect on cytotoxicity. In addition, only two patient samples were used which is not conclusive. A larger number of patient and control samples needs to analyzed (e.g. 7 vs 7).
While one key finding of the article is the elevated expression of TIGIT in T-reg cells in OS tumors, the confirmation of its significance, the functional validation, did not produce significant results. This must be substantiated.

Finally, the overall English in the article text as well as figure legends and figures themselves needs to be improved. Furthermore, great care should be taken to avoid typing mistakes in the main text or figures (e.g. Fig S2A “Paekinsons Disease”).

Overall, the present study contributes a novel single cell transcriptomics data set to the field, which indeed might be of interest to other researchers to be used as a reference. It is also the first study to employ this technology to study OS. The computational analyses of this data set however require substantial improvements as well as the experimental set up regarding sample size (see comments above), and a clearer, more detailed description in the methods section. Despite the usage of trajectory inference and GO term analysis, the present study falls short in conclusively describing the molecular signatures of all cell populations in all different samples as well as to perform a representative analysis across similar sample sizes per sample type. The possible effect of drugs from combined chemotherapy on transcriptional signatures but also cellular heterogeneity is not taken into account and is a weakness of this study. It is furthermore often unclear, whether identified cell populations can be further subdivided into different subclasses or cell states, which in turn might be

relevant to describe the tumor microenvironment in a more refined fashion. Albeit performing single cell analysis in a novel patient tissue, the conclusions regarding cell populations but also the claimed clinical utility are rather weak.

The following are our point by point responses to the comments of the reviewers.

Reviewers' Comments:

Reviewer #1 (Remarks to the Author):

This manuscript presents a novel and worthwhile study of heterogeneity in osteosarcoma, using single cell RNA sequencing suggesting the presence of various cell types in osteosarcoma tumors. While the results are interesting several issues need to be addressed in order for the manuscript to be considered for publication. Specifically, critical methodological details are missing, and the data and results need to be re organized and presented in a way that conveys the most important biologic or clinical inferences from the data. Critical algorithms and methods are not explained in adequate detail and abbreviations are not spelled out. The contrast between the various cell types is not presented in a clear enough manner, or in a manner that relates to features of osteosarcoma biology or clinical behavior. Finally, some terms and vocabulary is not always clearly explained.

ANSWER: 1A1:

We thank the reviewer for these helpful comments. As suggested, in the revised manuscript we have provided the missing methodological details, and reorganized the results to bring out the message of the article eloquently. We have also performed an intensively updated analysis, described and discussed their results along with the detailed analysis code and the source data. The language was carefully edited, and abbreviations are spelled out. We will actively respond to any further comments.

Some specific issues and examples that need to be addressed.

Perhaps a "summary figure" can be shown presenting all the subgroups and cell types and the methods used to identify them in one graph with their relative abundance as well as the main biologic themes represented and whether each subgroup corresponds to any specific biologic or clinical feature of the disease. Such a figure can be shown at the end of the Results, and may provide a more clear summary of the most important findings of the study. The currently presented detailed Figures, while containing potentially important information, do not contribute to a clear enough understanding of the main and more relevant findings of the study.

ANSWER: 1A2

According to the reviewer's suggestion, we have added a summary figure about the study design as Figure 1a. We have also provided more detailed information about the biological functions, distribution of the cellular clusters in individual patients, and other important information in the text and the figure in the revised manuscript. We have again generated a table and a figure (Suppl table 3, Suppl Figure 15) to summary the main clusters and the subclusters of the cells in OS tumors studied. We hope that the figures and tables will make our results clearer.

Line 76-78: "We found that the TME....suppressivity than primary tumor tissue". This meaning of this sentence is unclear.

ANSWER：1A3

We apologize for the unclear statement in our manuscript and we have revised this and other sentences thoroughly to remove the ambiguity, following the suggestion.

Line 130: What is UMI?

ANSWER: 1A4

The UMI is acronym for unique molecular index. The UMI counts represent the absolute number of observed transcripts (per gene, cell or sample) in the scRNA-seq data, which indicates the total sequenced molecules in the scRNA-seq. We have expanded the abbreviations in the revised manuscript.

Line 135. The Seurat program needs to be referenced with appropriate literature.

ANSWER: 1A5

We apologize for the missing information, and we have now cited the Seurat program in our revised manuscript (version 3.1.5; http://satijalab.org/seurat/) in R software (version 3.6.1).

Details of how the Seurat algorithm and the t-SNE method were applied are necessary. If these details are too many to easily report, they should be listed in a Supplementary Methods file. Findings from these methods are often very sensitive to specific parameters and tuning. Without more details of how the algorithms were applied, it is difficult to assess the validity of all results. For example, how did the algorithms decide about the number of subgroups and objects in each analysis and how was it concluded that these subgroups are statistically robust?

ANSWER: 1A6

The reviewer's comments are highly appreciated. We agree with the reviewer that the results are usually sensitive to specific parameters and the tuning in the single cell sequencing analysis. We applied the detailed protocols by [Butler et al. Nature Biotechnology, 2018 Jun;36(5):411-420] for the Seurat, and by [Trapnell et al. Nature Biotechnology, 2014 Apr;32(4):381-386] for monocle v2.0 programs, in our current study. These two bioinformatics analysis pipelines are well accepted and commonly used in the analysis of the subgroups in single cell sequencing experiments and the trajectory analysis in highly profiling studies. We divided the cells into clusters based on the results coming from t-SNE algorithm-based studies.

To validate the robustness of the subgroup, clusters and the identification of the clusters, we checked the gene expression profiling of specific markers among the subgroups, referring the well-accepted canonical markers (such as in Suppl Table 2). We then calculated the overall gene features for each clusters and subclusters (the top 20 genes are listed in Suppl Table 3). Further we checked these genes for the functions with regarding the identification of the clusters and subclusters, with publicly available data and reports as references. We not only validated the identification, but also revealed a series of substantial new information, and some of the result were validated experimentally. Please see the results and the discussions throughout the revised manuscript.

Meanwhile, we provided the original data and the code that we used in our analysis pipelines along with the manuscript. We also made these information publicly available, for the readers and the reviewers. We hope that these measures will take care of the reviewer's concerns.

Line 142: t-SNE needs to be spelled out.

ANSWER: 1A7

We apologize for the missing information. We have now provided the full spelling of each abbreviations at the first time when it is appeared, according to the reviewer's suggestions.

How was copy number variation (CNV) estimated using RNAseq data? Were there genomic DNA sequencing data also obtained?

ANSWER: 1A8

We apologize for the unclear statement in the manuscript. We inferred the copy number variation of single OS cells based on the scRNA-sequencing data with the inferCNV package by R package developed by the broad Institute [https://github.com/broadinstitute/inferCNV/wiki]. This method has been widely used in recent years to obtain the CNV patterns of the scRNA-seq data, such as in glioblastoma, head and neck cancer, pancreatic cancer, melanoma and lung cancer [Tirosh, et al., Science. 2016 Apr 8;352(6282):189-96; Puram, et al., Cell. 2017 Dec 14;171(7):1611-1624.e24; Peng, et al., Cell Res. 2019 Sep;29(9):725-738; Durante, et al. Nat Commun. 2020 Jan 24;11(1):496]. A recent study published by Durante et al. has identified a good consistency between the scRNA-seq data and the single cell DNA sequencing method, suggesting that the values of scRNA-seq analysis can estimate the copy number variation[Durante, et al. Nat Commun. 2020 Jan 24;11(1):496]. In their study, the authors have also developed a sophisticated method named Uphyloplot2 plotting algorithm to view and get the evolutionary tree of the cluster tumor cells for the uveal melanoma.

In our current study, we performed the CNV analysis in the osteosarcoma cells following guidelines of Durante et al. [Durante, et al. Nat Commun. 2020 Jan 24;11(1):496]. We noticed that there were distinct subclusters of tumor cells with gain or loss of different chromosome arms. Importantly, we noticed the osteoblastic and chondroblastic tumor cells in the same OS lesions possess the same pattern of canonical CNV events and non-canonical CNV status in the subclusters (Figure 3), suggesting that the two types of malignant cells share the same origin, and that osteoblastic tumor cells might have been derived from the trans-differentiation from the chondroblastic tumor cells (Figure 3). This result is the first report for malignant OS cells, but not surprising, referring the previous findings that the (normal, not malignant) chondrocytes could undergo a direct transdifferentiation process into the (normal)

osteoblast during the endochondral ossification in healthy bone formation [Aghajanian P. Bone Res. 2018 Jun 14;6:19; Liu CF. Semin Cell Dev Biol. 2017 Feb;62:34-49]

We acknowledged that the inferCNV method is still short of high resolution compared to the genomic DNA sequencing data due to low coverage of the genes, and that the single cell whole-genomic sequencing analysis are warranted to comprehensively detect the genomic mutations in the osteosarcoma patients, which is our ongoing project and we hope to get a solid picture for the genomic CNV pattern and evolution of the 2 malignant OS lineages, in the osteosarcoma patients in the near future.

Line 169. What is the Monocle 2 program and is there a citation for this?

ANSWER: 1A9

We apologize for the missing information in our manuscript. Monocle 2 (version 2.14.0, embedded in as R package) is a commonly used unsupervised algorithm to infer the transcriptome dynamics with the single-cell RNA-Seq data. The monocle 2 has been widely used to recover single-cell gene expression kinetics from a wide array of cellular processes, such as differentiation, proliferation and oncogenic transformation [Nature Biotechnology volume 32, pages381–386(2014)]. We have now added the related information and the references in our revised manuscript.

Line 255: The concept of trajectory analysis is unclear. What is "pseudo-time"? This analysis may also benefit from a dedicated graph showing how it is done and summarizing what it reveals.

ANSWER: 1A10

The pseudo-time is defined by the trajectory analysis to infer the development or differentiation status of the cells between two cell states or cell groups. During the biological transition of the cells, the gene expression pattern is reconstructed, with some genes getting silenced while the others are activated, which usually is a gradual or step by step process of linear or with branches when all cells are considered.

However, it was usually hard to get the pure cells in the distinct status or differentiation time. In the single cell sequencing studies, it was hypothesized that the single cells we analyzed may have different transitional status, some of the cells may be at the end of the transition, some cells may be at the initiative phase, and some others may still be under the ongoing process. The cells were aligned according to the gene expression patterns, so that the cells could reflect the trajectory of the transitional process involved, with gene expression pattern regulating the process. The cells were ranged according to the gene expression pattern, but not as per the real differentiation time of the cells. Thus, the status of the cells was named as the “pseudo-time”, which indicates the earlier or the later state in the transition. The "pseudo-time" trajectory analysis can reflect the transitional patterns of the system, with the genes contributing to each part of the process.

Abbreviations such as CAFs (and all its variants such as capCAFs, myCAFs, apCAFs) need to be spelled out not just in the Abstract, but also the first time they appear in the main manuscript.

ANSWER: 1A11

We have now added the expansions of the abbreviations in the revised manuscript to make it easier for the reader.

The TIGIT blockade section is confusing. What type of cells were used? The body of the manuscript reports that this was done on "OS cells", whereas Figure 7 legend states that it was done on "breast cancer cell lines" and the Figure text reports the U2OS cell line.

ANSWER: 1A12

We apologize for this error. It was a clerical error that was overlooked. The method was modified from the article [David Stahl et al. OncoImmunology. Volume 8(12), 2019. e1674605], which studied breast cancer cell lines. In our experiment, we used definitely the OS cells. We did the experiment with the donated bloods from patients

BC3 and BC16 for anti-TIGHT blockage. Now we modified the legend of Fig. 7(f) clearly: (f) Blockade of T cell immunoreceptor with Ig against TIGIT increases the specific lysis, tested with the CD3+ lymphocyte from the peripheral blood of the 2 patients BC3 and BC16.

What was the blood that one study subject donates (line 104) used for? In the Methods section, it is stated that it was used to explore the effect of anti-TIGIT blockage but the respective paragraph in the Results section does not include any reference to blood.

ANSWER: 1A13

We apologize for failing in giving the full details. In fact, we used the methods for blood isolation and for anti-TIGIT blockage in accordance with reference [Stamm H, et al., Oncoimmunology.2019 Oct 12;8(12):e1674605]. We did the experiment using the donated bloods for anti-TIGHT blockage using blood from patients BC3 and BC16. Now we modified the legend of Fig. 7(f) clearly: (f) Blockade of T cell immunoreceptor with Ig against TIGIT increases the specific lysis, tested with the CD3+ lymphocyte from the peripheral blood of 2 patients BC3 and BC16.

Please also refer the ANSWER 2A12.

The overall language (use of English) of the manuscript needs to be reviewed in detail and improved.

ANSWER: 1A14

According to the reviewer's suggestion, we have revised the manuscript thoroughly, and hope that these measures meet the required standards.

Reviewer #2 (Remarks to the Author):

The authors correctly claim that their study is the first to employ single-cell RNA-sequencing to study Osteosarcoma (OS). They furthermore claim to utilize this technology to explore the molecular mechanism of OS as well as the tumor microenvironment (TME). They then describe the clusters they found which are formed by the 110 745 single cell profiles as well as state that TIGIT is expressed in 11 samples and is particularly overexpressed in T-reg cells. The then isolate CD3+ T cells and block TIGIT signaling in those cells which according to the authors yields improved cytotoxicity of Cytokine induced T cells. Their approach to use single cell resolution combined with trajectory inference methods to study OS cell heterogeneity is very interesting in particular since this study provides novel insight into the transcriptional cell types in OS and may indeed be of relevance as a reference for other studies.

ANSWER:

We very appreciate the very informative comments from the reviewer, which gives us a chance to improve the manuscript.

Q1. However, there are some shortcomings of the present study that need to be addressed. First of all, while the total number of single cell transcriptomes profiled appears reasonable, the representation of primary OS tumors (7) compared to recurrent OS tumors (2) and pulmonary metastasis (2) is much higher and it is absolutely crucial to include similar numbers of cells from each of the conditions to be able to perform reliable comparisons, in particular when aligning cells along a pseudo-time. The authors need to analyze at least the same number of patient samples for each condition (7). The authors comment on the inability to capture cancer stem cells due to their low number. An increased sample size will facilitate to confidently rule out that lack of stem cell capture is due to underrepresentation of samples or small sample size.

ANSWER: 2A1

We appreciate the suggestions from the reviewer. Indeed, the reviewer's point is valid. The number of samples from different types of lesions (*in situ* or primary, lung

metastatic and recurrent OS) were different, and the number of cells for lung metastatic OS and recurrent OS were less than that of the primary OS. And we believe that an increased sample size, as one of the possible alternative strategies, will help to confidently clarify the stem cell issue, which may be the next project to focus on (and we deleted any discussion on stem cell in this manuscript).

We planned to collect same number of the 3 types of lesions, and to collect a few paired /matched lesions, ex. primary OS lesion / metastatic OS lesion / recurrent OS lesion, from the same OS patients. However, in spite of lot of efforts (all were under the ethical regulation, and followed the international clinical standards for OS), it was very hard to collect samples for metastatic and recurrent OS, because these patients were much less likely to get operated according to the clinical philosophy. Meanwhile it is even harder to get paired / matched samples.

In addition, the bone cancer biopsy collected through fine needle aspiration, which is the standard procedure for the primary diagnostics of OS, is insufficient for isolation of living cells that meet the requirements for high-throughput single-cell RNA sequencing. Actually, even if one gets a good amount of biopsy from operation (operation follows the chemotherapy, which is required by the international standard protocol, so the tumor cells are usually not in a good condition), it is with little success one can isolate the required quality and quantity of living cells for the sequencing.

Therefore, this is the best panel of samples that are accessible, and probably this is the reason why there is no single cell sequencing report available for this disease by now. On the other hand, in the field of single cell RNA-sequencing, using replicate samples (2 independent samples) to represent a given status of a disease or biological process is not unusual (i.e. it is acceptable) in single cell sequencing researches, considering that thousands of cells are analyzed for each sample.

Importantly, we mostly focused on the cellular transition mechanism between different subgroups of the osteoblastic and chondroblastic cells within the same samples (same lesions) from patients BC20 and BC22 (Fig. 3). Besides others, we also traced the cellular trajectory involved in the differentiation from the myeloid cells into the

osteoclast cells in the revised manuscript (Figure 4). Furthermore, we identified the subclusters of the MSC cells that express the markers CXCL12, THY1, and CD10 (MME), which could be the progenitor cells in the osteoblast or the chondrocyte cells.

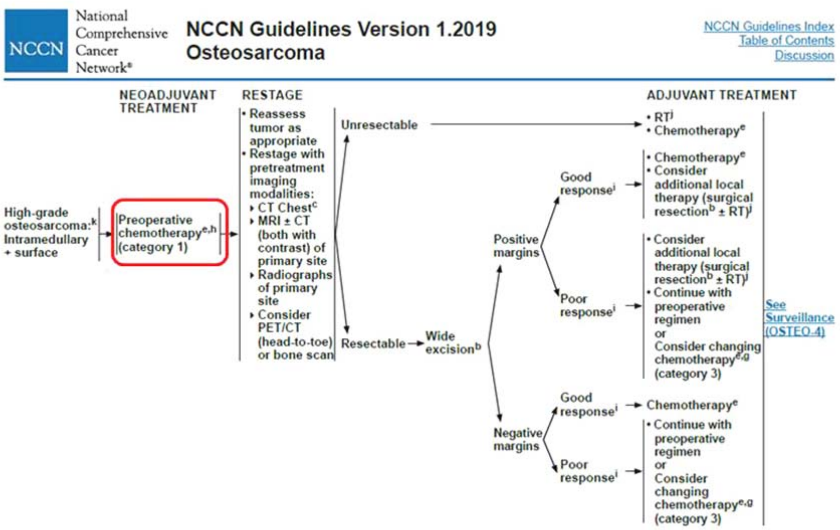

Nevertheless, in the manuscript, we also compared the differentially expressed genes and the related cell clusters between the lung metastatic OS cells to the primary osteoblastic OS cells, and the recurrence and the primary osteoblastic tumor cells (Figure 2). The enriched GO terms and pathways were also determined, which are interesting. Because of the consistency of the results obtained for the 2 independent lesions of metastatic OS, and of recurrent OS lesions compared to the 7 primary lesions in the analysis result, we trust that the features obtained for these 3 types of lesions are representative. Also, in the revised version, we pointed out that some results may have certain influence by the heterogeneity between the individuals, and an independent investigation with panel of paired samples or more samples may be required to valid the results, like most other firstly reported results (see DISCUSSION in the revised manuscript, and see more in answer 2A2, 2A3).

Seven of the selected patients were under combined chemotherapy as well as surgical therapy. Cellular heterogeneity and underlying transcriptional signatures must therefore be seen in the light of this continuous drug treatment. It would be of great interest if the authors investigated further which cell populations are particularly

affected by the drugs and how combinatorial drug treatment is encoded in the transcriptome of different cell populations. In addition, gene signatures related to sample type, isolation strategy and treatment might confound cell type calling- this needs to be discussed and addressed by the authors. Furthermore, the authors need to demonstrate the degree of correlation of each cell type between the different patient samples.

ANSWER: 2A2

The reviewer is correct that all the patients had received the combined chemotherapy treatment prior to collection of the tumor tissue samples for scRNA-sequencing analysis (Figure 1), please refer the answer in 2A1.

However, it is difficulty to collect the ideally expected samples, untreated OS lesions for the sequencing. Firstly, untreated samples were collected with fine needle before diagnosis of OS, and with this format of biopsy, it is impossible to get sufficient quality and quantity of cells for the high throughput scRNA-seq. Secondly, the OS patients were subjected to chemotherapy before operation, following the international standard. As we lack the untreated cells in each subgroup cells, it was impossible for us to determine the influences of the chemotherapeutic treatments on the subcluster cells in detail.

We completely agree with the reviewer that addressing the specific cell clusters and genes that are responsible for drug resistance is an important task, and is potentially very useful to improve the treatment of this cancer. We will manage to investigate this issue, for example using patient-derived xenograft (PDX) mouse model, *in vitro* cells obtained from the fine needle biopsy, or any other strategies permitted by the clinical standard and ethical rule, which could be an independent project.
Regarding a comparison of different combinations of chemotherapy, suggested by the reviewer, it may also applied when a sufficient number of cases for variants of treatments are collected.

Nevertheless, we considered that the cell clusters and gene regulation message obtained from the patients should have the influence of the medicine. Therefore, we

interpreted that the conclusions of this study should be regarded as the outcome of possible chemo-resistant selection onto these patients (Please see the DISCUSSION section). On the other hand, our samples all were collected from surgical operation, no comparison was applied.

The relationship of specific cell types and their subcluster was particularly analyzed in the revised manuscript, particularly between different the types of lesions (samples), and between the two types of OS (osteoblastic OS and chondroblastic OS). Also, please see the answer "2A3" for the analysis, including the correlation of samples and clusters.

The authors need to provide more detailed information in the methods how quality control was performed to exclude single cell libraries from further analysis. They nicely show representations of the main cell populations for each patient sample used. Since a fixed embedding is used for this visualization, it will be important to see a t-SNE or UMAP representation of all patient samples labeled by experiment/ patient ID/ experimental run to demonstrate the absence of batch-to-batch variation. They also need to state average sequencing depth, UMI and gene count per cell.

ANSWER: 2A3

The reviewer pointed out an important issue about the quality control, pre-processing of the cells and the batch effects of the scRNA-seq data analysis. In our current study, we followed standardized pipelines in the data processing, including the constructing the cell-gene matrix with the CellRanger pipelines provided by the 10x genomics and normalization, removal of potential doublets, and removal of those cells with relatively higher mitochondrial genes (>= 10%) and the low number of detected genes (< 300 per cell). We integrated the data from individual lesions with the Harmony algorithm, which were developed by Korsunsky.

et al. [Nat Methods. 2019 Dec;16(12):1289-1296]. We also checked the splitted t-SNE and UMAP results from individual patients and found a well concordance of the cell subgroups between individual lesions, with variants of rate of cells in different lesions, which are reasonable considering the inter-tumoral heterogeneity that is well documented for other cancers. This analysis is suggesting that the Harmony algorithm

could remove the batch effects between the patients. Therefore, the heterogeneity between individual lesions are clearly considered and it does not influence the conclusion.

According to the reviewer's suggestion, we have provided the average UMI, and the gene count per cell, and clustering result from individual patients in our revised manuscript (the RESULT section and Supplementary Figure 1).

In Figure 1A**,** the myeloid cell cluster is already split up in initial t-SNE representation, suggesting either sub-clusters to myeloid cluster or entirely new populations. The authors need to computationally show that after initial clustering, there is not more biological diversity in the myeloid cluster and that this cluster does not contain more distant cell types. The authors state that the TP53 pathway is markedly "disordered in subgroup 5" in Figure S2A- the use of the word "disordered" needs to be explained.

ANSWER: 2A4

We agree with the reviewer that the myeloid showed an already split up clusters in the initial t-SNE figures. In the revised manuscript, we extended the previous studies and further explored the subgroups of the myeloid cells and identified 10 subgroup cells in the myeloid cells including the monocytes, macrophages, DC cells and also the neutrophil cells. We performed the GSVA analysis to infer the biological activities in the individual cell group, and we also performed the GSVA analysis for the activation of 3 clusters of TAMs according to the methods used by [Chung et al., Nat Commun. 2017 May 5;8:15081]. We again compared the DC activities in the three identified DC clusters, and we noticed that $CCR7^+$ was associated the maturation state of the DC cells. Therefore, we confirmed with references from the publicly available data, canonical markers, and gene functional analysis that these current subclustering results are true and informative.

Regarding whether or not there is more biological diversity, more distant cell types in these subclusters obtained, this is a kind of philosophic question. When there are data for more cells available, and someone is interested to address further subsets for a

given subset/s, there will always be a space to study. We hope that the revised manuscript clarifies the cellular activities of the myeloid cells.

Regarding the statement that the TP53 pathway is markedly "disordered in subgroup 5" in previous Figure S2A, we apologize for the unclear statements in our manuscript, and we have now removed this unclear description accordingly and believe that the revised manuscript makes the sentences clear.

Furthermore, the authors use GO terms to infer putative signaling or functionality onto the transcriptome data. However, the GO term analysis in Figure S2A does not provide a clear distinction between the different populations and is therefore not informative. E.g. metabolic and proliferative OS as well as bone-matrix remodeling and ossification OS greatly overlap. The separation of these populations by GO term analysis is not conclusive and needs to be substantiated. Cluster analysis as in Figure S2B allows to separate those cells however, only rather few genes actually contribute to this separation.

ANSWER: 2A5

Very appreciate the comments from the reviewer, which lets us to re-analyze and doubly verify the results. According to the suggestions, we have re-performed the studies on the differentially expressed genes between osteoblastic tumor cell clusters and the chondroblastic OS tumor cell clusters using a modification of the competitive gene set enrichment analysis (GSEA) method developed by [Cillo et al., Immunity. 2020 Jan 14;52(1):183-199.e9]. In brief, this method determined the mean gene expression levels in each cell cluster, and assessed the log fold-change for the gene expression levels in the given cluster and the mean expression levels of the same gene in all cells outside the given cluster as the test statistic. Gene sets used for analysis were derived from the MSigDB collections of 50 hallmark gene sets (H: hallmark gene sets), which summarized and represented specific well-defined biological states or processes. Therefore, we removed the previous Figure S2A and B, and replaced them with new figures (Figure 2 c, d, f, and g), to respond to the comments.

We also compared the gene expression profiling from different types of lesions of osteoblastic OS cells (lung metastasis vs. primary and recurrent vs. primary; Figure 2E) and between chondroblastic vs. osteoblastic cells (Figure 2H) using the non-parametric Wilcoxon rank sum test. The output differentially expressed genes of the cell groups were further analyzed with GO terms enrichment using the hypergeometric tests implemented in the clusterProfiler package of R, and the results were now shown as Supplementary Figure 6h, I and J, respectively.

We have added the detailed methods in our revised manuscript, and we have updated the content in the figures along with the versed manuscript. We hope these bioinformatic analysis can classify the biological activities of the cells in each cluster.

The exploration of trajectories is very interesting, however, the referencing to individual genes in the actual figure needs to be made clearer as well as the figure themselves: Figure 2D needs to be subdivided and genes as well as GO terms which do not contribute to further analysis, should be remove to deconvolute the figure.

ANSWER: 2A6

According to the comments from the reviewer, we have thoroughly revised the manuscript, and Figure 2D in previous manuscript version has now removed.

In the revised manuscript, we performed the trajectory analysis to infer the cellular status transition for the trans-differentiation from the chondroblastic tumor cells into osteoblastic tumor cells (Supplementary 9) in patients BC20 and BC22. The genes differentially expressed along with the pseudo-time of cellular trajectory model were identified with the Monocle 2 algorithm [Saelens W. et al., Nature Biotechnology 2019 May; 37(5): 547–554], and these genes were grouped into clusters according to their gene expression pattern based on the Monocle 2 algorithm.

To provide an overview of the key biological functions of the genes in each cluster, the enriched GO terms for those genes in each cluster were analyzed with the

hypergeometric tests implemented in the clusterProfiler package of R. We also performed the trajectory analysis of the cellular transition of the OC derived from the myeloid cells in the TME of OS using Monocle 2 algorithm (Figure 4e and f) similarly. We hope the revised manuscript will address the reviewer's concern about the trajectory analysis results.

Pseudo-time analysis of OC maturation nicely shows highest expression of CD74 and TOP2A in progenitor OCs. These cells subsequently serve as the origin for trajectory building. The authors need to provide more information about selecting seed cells plus gene signatures which serve as the origin of the trajectory.

Furthermore, since trajectory building is not trivial and strongly depends on the trajectories to be build and can differ between available methodologies (bifurcating vs multifurcating trajectories), the author must test different available solutions and carefully evaluate the trajectory building outcome. Since many conclusions of this manuscript are founded on this type of analysis, it is absolutely crucial to ensure that the utilized algorithm reflects the underlying biological processes in the most conclusive way.

ANSWER: 2A7

Thank you for the insightful comments. It is true that the trajectory could be influenced by the methods that were used in the analysis. In the current study, we chose the monocle 2 algorithm to infer the trajectory analysis. A recent study reported in [Saelens W. et al., Nature Biotechnology 2019 May;37(5):547-554] has compared 45 trajectory inference approaches in 100 real and 229 synthetic datasets for cellular ordering, topology, scalability, and usability. They noticed that the performance of a method can vary between datasets. The monocle 2 applied an advanced nonlinear reconstruction algorithm called DDRTree, which can expose the branches more powerfully. In addition, the monocle 2 also provided branch expression analysis modeling (BEAM), a new test for analyzing specific branch points to identify branch dependent genes [Papalexi, et al. Nat Rev Immunol. 2018 Jan;18(1):35-45]. Furthermore, the Monocle 2

has a good publication track record in many high impact journals; therefore, we applied the monocle 2 to infer the trajectory of the cells in the current study.

It has been reported that the osteoclasts (OCs) were derived from the monocyte/macrophage lineage (covered in myeloid lineage) upon stimulation of two essential factors, the monocyte/macrophage colony stimulating factor (M-CSF) and receptor activation of NF-κB ligand (RANKL) [Feng, et al. Bone Res. 2013 Mar 29;1(1):11-26]. M-CSF binds to its receptor c-Fms and activates distinct signaling pathways that stimulate the proliferation and survival of OC precursors and their mature cells. RANKL, is the primary OC differentiation factor, and promotes OC differentiation mainly through controlling gene expression by activating its receptor, RANK. Basing on the message described above, the myeloid cells that expressed the myeloid cell markers including CD74 and CD14 could be set as the root stage of the trajectory for our trajectory analysis of the cellular differentiation of osteoclast. Interestingly, we found that these progenitor cells also have relatively higher expression levels of TOP2A and HMGB1, the markers of cellular proliferation, suggesting that these cells may be stimulated by the M-CSF and RANKL secreted by the osteoblasts in the OS tumor microenvironment [Akiyama, et al. Mol Cancer Ther. 2008 Nov;7(11):3461-9], and under the differentiation process into OCs.

The OC cells have been suggested to be involved in the bone destruction and invasion in the osteosarcoma lesions, which may serve as novel therapeutic target for OS in future. So they are clinical important. In the current study, we in single cell level found that the CD74 levels were relatively high in the OC progenitor, and were low in the mature osteoclast cells, suggesting that the antigen processing activities of the cells were lost during the differentiation from OC progenitor) into mature OC cells. Combining with other analyses, our data suggests that the OC progenitor possess the function for antigen presentation, while mature OCs lose this function for antigen presentation while gain the function for bone destruction and invasion, which is associated with lung metastasis OS. Please see also the ANSWER 2A8.

In Figure 3E, the authors describe GO terms to describe biological process as they change following OC differentiation. As opposed to earlier analyses in this manuscript,

the selected GO terms are either sharply present or absent at both endpoints of the trajectory. None of the GO terms show gradual acquisition or decline along differentiation axis. The authors need to provide relevant GO terms which gradually alter over time as it now appears to be the transition between two cell stages only. Figure S4 is not mentioned in the text.

ANSWER: 2A8

In the revised manuscript, we added a series of new figures, including we also added the cell numbers and ratios in each cluster in the new Figure S5, which display the detail of the old Figure S4. Meanwhile, we removed the old Figure S4.

In the trajectory analysis, we wanted to trace the biological pathways that varied with the differentiation process of OC from CD74+/CD14+ monocytes (belonging to a type of myeloid) to mature OC, presented in the previous Figure 3E. We detected the genes that varied with the trajectory pseudo-time with the Monocle 2 algorithm [Nature Biotechnology volume 37, pages547–554(2019)], which indicated different differentiation status of the cells in order, and classified the genes into subsets according to the gene expression patterns (increase or decrease along with the pseudo-time). The updated results were showed in the new Figure 4, and Figure S10.

To provide an overview of the key biological functions of the genes in each cluster, we performed the gene functional GO enrichment analysis of the genes in subsets and provided the top enriched GO terms in the revised manuscript. We found that the down-regulated genes were associated with antigen processing and presentation, responses to interferon gamma treatment and the DNA replication and mitotic nuclear division (Figure 4e, cluster 3). In contrast, the genes contributed to bone resorption, osteoclast differentiation and hematopoietic stem cell differentiation were significantly increased along with the cellular differentiation to mature OC, which was inconsistent with our expectation (Figure 4e, cluster 1 and 2). Furthermore, we noticed that the critical transcription factors involved in OC differentiation including NFATC1, SPL1 and FOSL2 were significantly increased (Figure 4f), while the genes related to DNA replication and genes related to monocyte activation including MAF, MEF2C and the differentiation related transcriptional factors including JUN, FOS etc. were

significantly reduced (Figure 4f). These results provided a comprehensive view of the differentiation process of OC from monocytes.

We also apologized for the unclear statement in our previous manuscript. In the updated Figure 4e, each line indicated the scaled expression level of individual genes that changed along with the pseudo-time of the cellular trajectory. These genes were clustered into 4 clusters based on their gene expression pattern according to the Monocle 2 algorithm. The GO terms of the genes in each cluster were analyzed with the hypergeometric tests implemented in the clusterProfiler package of R. As the genes were gradually increased or reduced along with the trajectory, we hypothesized that the GO terms show gradual acquisition or decline along differentiation axis. We hope these responses could address the reviewer's concerns.

Next, the authors use Seurat to cluster myCAF and apCAF. Unfortunately, based on the clustering they present, not two but rather 7 distinct gene signatures can be identified (Figure 4C). The authors must clarify the discrepancy between the t-SNE distribution of two populations compared to the finer, but unfortunately ignored clustering by Seurat. The results of this part are as of now inconclusive.

ANSWER: 2A9

Appreciate the comments. We have updated the analysis pipeline, and three subclusters of CAF were identified, as suggested by t-SNE clusters (now the early Figure 4C was removed, please see the replacement: the new Figure 5d, e and f). Please note that the subcluster 1 seems separated in the plot, but we must consider that this plot is a 2D plot while the actual relationship is captured in 3D. Specifically, the COL14A1+ matrix fibroblast, the smooth muscle -like cells and the third type. The gene patterns of each CAF subcluster were also dissected (Supplementary Fig. 12f). Compared to the other 2 clusters, the cluster 3 showed the feature of myofibroblast. Interestingly, the subcluster 3 showed relatively high expression of osteoblast markers IBSP and SPP1, suggesting that this subcluster in the osteosarcoma lesions may play an osteoblast-like function. The functions of the distinct cell groups were shown in the revised manuscript.

In figure 6 C, CD8+ T-cells and CD4-/ CD8- T-cells basically do not differ in gene expression. In the t-SNE representation (6A) both cell populations might separate. Gene signatures need to be provided which actually delineate both cell populations.

ANSWER: 2A10

We seriously considering the comments of the reviewer with the differentially expressed genes between CD8$^+$T and CD4$^-$/CD8$^-$T cells, and re-checked the data with the methods said above. In the revised manuscript, we have updated the analysis according to our latest result and changed the previous Figure6 C to current Figure7 c for this issue. We found that the CD8+T cells and CD4-/CD8-T cells were clearly separated in clusters. We identified the genes that were differentially expressed between the 2 cell subclusters. We also performed the gene set variation enrichment analysis (GSVA) [Hänzelmann, et al. BMC Bioinformatics. 2013 Jan 16;14:7] to identify the enriched biological functions of the cells, in particular, the cytotoxic, exhausted, regulatory, naïve and co-stimulatory T cell functions. The results suggested that CD8+T cells were enriched with the cytotoxic activities and a small proportion of the cells were associated with the exhausted status, highlighting the tumor suppressive environment of the osteosarcoma tumor.

Interestingly, we also found that T-reg cells have shown a significant enrichment of the exhausted activities. The T-reg cells were characterized with relatively higher IL2RA, FOXP3 than other cells, and the cells in the cluster also have relatively higher TIGIT and CTLA4 expression, suggesting that the T-regs have significant tumor-suppressor activities in the tumor microenvironment of osteosarcoma tissues.

The cell-based assay to assess blocking of TIGIT signaling on cytotoxicity is presented as being statistically significant. However, in Figure 7B, none of the comparisons Control vs. TIGIT inhibition shows a significant effect on cytotoxicity. In addition, only two patient samples were used which is not conclusive. A larger number of patient and control samples needs to analyzed (e.g. 7 vs 7).

ANSWER: 2A11

Thanks for the reviewer's helpful comments. We had changed the previous Figure7B to Figure7f now.

Regarding the concern of the reviewer about the cellular cytotoxic activities of TIGIT blocking in tumor cells, so far, many clinical trials involving the immune check point inhibitors, such as Pembrolizumab, Nivolumab, showed promising treatment effect on OS, but less than 10% patients were benefited from this therapy. The results of SARC028 indicated that only 2 out of 40 (5%) patients with bone sarcoma had an objective response[Pembrolizumab in advanced soft-tissue sarcoma and bone sarcoma (SARC028): a multicentre, two-cohort, single-arm, open-label, phase 2 trial [Hussein , et al. Lancet Oncol. 2 017 Nov;18(11):1493-1501]. It has been a challenge to identify a better molecular target for OS therapy.

In this study, we tried to test whether TIGIT blocking would inhibit the tumor suppressive activities of T-regs and whether it would reverse the cytotoxicity of the exhausted $CD8^{+}$T. Basing on the concept of precision medicine, not all patients may be responsive to anti-TIGIT antibody, like the case in PD1-based therapy. Here based on the scRNA-seq analysis, we chose 2 patients who had more than 10% of TIGIT in $CD3^{+}$ T cells for the test, and we expect that these type of patients would be the appropriate sub-set of patients for potential TIGIT therapy in the future.

In the current study, the cytotoxicity was evaluated according to the article titled [Stamm H, et al., Oncoimmunology.2019 Oct 12;8(12):e1674605]. With the cells from the two patients showing relatively higher (than other patients) TIGIT expression of the immune cells based on scRNA-seq, our experiment showed that the TIGIT blocking significantly increased the T cytotoxicity activities in the cellular models. For the current study we could not add more patients due to aforementioned reasons. We highly appreciate the suggestion of the reviewer, and we will further validate the efficiency of anti-TIGIT assay, and explore this mechanism with patient cells and with humanization animal model in a systematic fashion in our future study.

However, on this stage, the patient availability is limited because the incidence of OS was very low (ex. less than one-tenth of breast cancer), and due to the spread of novel coronavirus. A systematic investigation of the effect of TIGIT-blocking with a big panel of patients would be a different project.

While one key finding of the article is the elevated expression of TIGIT in T-reg cells in OS tumors, the confirmation of its significance, the functional validation, did not produce significant results. This must be substantiated.

ANSWER: 2A12

We highly appreciate the reviewer's comment. Besides a series of other discoveries, we have identified that FOXP3^{+} T-reg cells showed high mRNA expression levels of TIGIT. We have further checked the public data available and confirmed this finding. As indicated in Figure 7, we identified a cell cluster with FOXP3 expression (the marker of T-reg cells), in which the TIGIT expression levels were relatively higher than the other cells. These data suggested that the T-reg cells have relatively higher TIGIT levels in osteosarcoma tumor cells, which may serve as a novel therapeutic target for osteosarcoma. We did a primary test and in principle proved the hypothesis. Due to the limitation of the coverage and the focus of this study, we did not determine the biological functions of the TIGIT^{+} T-reg cells in this study. We are going to extend the investigation further in an independent project for re-confirmation of its significance, validation of the functional relevance, and the potential application in the therapy of OS patients (including identification of the subgroup of patients eligible for the therapy). The comments from the reviewer greatly encourage us to systematically accomplish this mission at the earliest.

Finally, the overall English in the article text as well as figure legends and figures themselves needs to be improved. Furthermore, great care should be taken to avoid typing mistakes in the main text or figures (e.g. Fig S2A "Paekinsons Disease").

ANSWER: 2A13

We apologize for the spelling mistakes and grammar issues in the manuscript. We have further revised our manuscript thoroughly and hope it will meet the standards required.

Overall, the present study contributes a novel single cell transcriptomics data set to the field, which indeed might be of interest to other researchers to be used as a reference. It is also the first study to employ this technology to study OS. The computational analyses of this data set however require substantial improvements as well as the experimental set up regarding sample size (see comments above), and a clearer, more detailed description in the methods section. Despite the usage of trajectory inference and GO term analysis, the present study falls short in conclusively describing the molecular signatures of all cell populations in all different samples as well as to perform a representative analysis across similar sample sizes per sample type. The possible effect of drugs from combined chemotherapy on transcriptional signatures but also cellular heterogeneity is not taken into account and is a weakness of this study. It is furthermore often unclear, whether identified cell populations can be further subdivided into different subclasses or cell states, which in turn might be relevant to describe the tumor microenvironment in a more refined fashion. Albeit performing single cell analysis in a novel patient tissue, the conclusions regarding cell populations but also the claimed clinical utility are rather weak.

ANSWER: 2A14

We thanks the reviewer for helping us improve the manuscript through his kind and informative comments. According to the suggestions, we have thoroughly revised the manuscript. We added more detailed information, such as method description, further deeper analysis, and result interpretation, in our revised manuscript. The GO analysis of the differentially expressed genes and gene set enrichment analysis (GSEA) were now performed to get the molecular signatures of the cell subgroups in the revised manuscript.

We acknowledge the possible effects of the drugs on the gene expression profiling and the cellular heterogeneity and are largely unknown, which need to be evaluated with more studies. In our study, the scRNA-sequencing was performed on the patients who had previously received chemotherapeutic treatment, which is one of the weakness of

our study; however, the current study has the following clinical treatment guidelines, and the findings in our current study may provide potential therapeutic targets for osteosarcoma patients, such as TIGIT blocking may prove to be an effective therapeutic method clinically.

We agree with the reviewer that the sub-cluster cells could also be divided into more clusters which may provide a clearer picture of the TME in osteosarcoma; however, due to the limited cell numbers in the current study, we clustered the cells into canonical major cell subtypes based on the t-SNE algorithm.

Furthermore, the detected gene numbers were also limited in the 10x genomics method. In the future, it would be of great clinical importance to evaluate the detailed status of the tumor immune microenvironment in osteosarcoma with the enrichment of the myeloid cells and T and NK cells with high coverage of genes using techniques such as the SMART-seq method. These would be our future prospects to pursue.

Reviewer #1 (Remarks to the Author):

The authors have generally answered and addressed the questions posed in the first review. I have some minor edits and clarifications to suggest.

INTRODUCTION

Page 4:
They quote an Overall survival rate 25%. That is really not correct. It is only correct for metastatic OSA, not for all osteosarcomas and certainly not for localized osteosarcomas. They should correct this statement. Survival for all osteosarcomas (unselected) is above 50%.

Also, Radiation treatment is not part of the standard or optimal treatment plan for osteosarcoma, which is treated with surgery and chemotherapy. Radiation is only used when surgery is absolutely impossible or for palliation. This should be corrected.

RESULTS:

Page 5:
10, 897 cells: This must be a typo, this count does not agree with counts stated for the same result in other sentences.

METHODS

Page 27
How many patients received PD-L1 therapy?
The authors provide somewhat confusing information. One sentence states that it was one patient on a prior trial, and another sentence states that all patients received PD-L1 therapy. This should be clarified.

Reviewer #2 (Remarks to the Author):

ANSWER: 2A1
… and we deleted any discussion on stem cell in this manuscript.
The authors removed the statement about missing stem cells, which might be simply due to lack of under-sampling of cells from metastatic and recurrent OS. The authors also state that these samples are too difficult to obtain to process additional specimen as well as that the fine-needle aspiration of cancer biopsies does not provide cells of sufficient quality and quantity for high throughput analysis. Different platforms exist which enable processing small samples such as FACS sorting into PCR plates. Furthermore, confirming cell type annotations via an alternative method (e.g. plate-based Smart-seq) would further strengthen the methodology underlying this manuscript and might provide an even more refined cell atlas of a previously understudied tissue by single cell transcriptomics.
Also, if the authors are concerned about sample quality upon biopsy, profiling nuclear RNA has proven to be very informative and to be less affected by sample isolation compared to cells.
ANSWER: 2A2
The authors state the lack of untreated controls for each subgroup which makes it impossible to infer treatment induced transcriptional alterations. Instead, if no control tissue is available at their institution nor from an international biobank, they could use the primary OB cells and state that they are used as an approximation due to the lack of the proper control.
The authors should provide evidence for transcriptional signature changes upon treatment as this would add significant value to the manuscript.
ANSWER: 2A3
The authors provide the demanded answers in their reply as well as in the results section. The t-SNE

plots of individual samples show the different cell types which are present in all samples. However, the UMI counts are particularly low for BC3 and BC2 and indicate that mRNA capturing/ amplification did not work as well for those two samples. Where these samples processed differently? Why did these two samples generate fewer UMIs and gene counts compared to the other samples?
ANSWER: 2A4
Good. The authors have further analysed this cluster and provided a definition for 10 subtypes within the myeloid cluster.
ANSWER: 2A5
The authors address the comments and re-analyse the data set for GO term enrichment. Now they show genes which distinctly mark the different clusters (2c) as well as distinct GO terms which show a distinct, cell type-specific pattern (2g). Unfortunately, in Fig 2d, the alphabetical ordering of GO terms makes it difficult to spot overall changes between the cell types. If possible, one could re-order the GO terms in 2c and 2g- similar to Fig. 2c.
ANSWER: 2A6
The authors addressed the comment by providing detailed description on how Seurat clustering and monocle 2 trajectory inference were performed.
ANSWER: 2A7
The authors did not test trajectory building using another algorithm, as suggested, and focused on monolce2. Their explanation is, that monocle2 is one of the most widely used software with a good track record. They reference Saelens 2019, which concludes that the choice of trajectory tool is dependent on the data set dimensions and trajectory topology. They state themselves that trajectory tool performance may depend on It would be interesting to see, whether another trajectory inference method would provide a similar picture of developmental trajectories in this particular case. Since central conclusions of this study are based on trajectory analyses, a scientific reasoning for the chosen method would appear natural.
ANSWER: 2A8
The genes which are strongest down regulated on Fig 4f are not the genes stated by the authors in the result section. They should comment on those genes (e.g. DBP, ID3,HES4, GLMP) and their biological relevance for the differentiation processes studied here. In the text, the authors state the following genes to be downregulated HMGB1, HMGB2, MEF2C, CREM and LITAF but it is unclear from the text to which figure they refer to. Gene LITAF for example is not in Fig 4f. Please comment and correct.
ANSWER: 2A9
OK.
ANSWER: 2A10
OK.
ANSWER: 2A11
In Fig 7f, graphs U2OS:BC16 and 143B:BC16 are exactly identical. Please explain. The authors need to clearly state in the results, that the two patients were selected based on high TIGT expression in CD3+ T cells. This is relevant to follow the rationale of testing for T cell cytotoxicity in their cellular model.
Given that suitable samples are sparse, the explanation for using these two patients is relevant. However, it would be crucial to see patients' cells performance in this assay with low TIGIT expression- based on the conclusion of the authors, the observed cytotoxicity should be less. This is a logic and necessary experimental confirmation of their hypothesis.
ANSWER: 2A12
See comment above to substantiate the conclusion that targeting TIGIT-high T-reg cells may present a novel therapeutic approach to OS, the authors should at least show a milder effect in their cellular assay in patient samples with lower fraction of TIGIT-high T-reg cells.
ANSWER: 2A13
OK.
ANSWER: 2A14
OK.
OTHER COMMENTS:

Typo line 100: total number of cells is too low, does not add with the following cell counts
Line 150: typo --> BC17 instead of BC107
Ordering of Fig 2d to be changed --> such as in “stair-case” like plot Fig 2c
Chromosomal lesions: 1p gain, 1q gain, 2q gain, 17q gain, 201 and 21q gain --> were they observed in OS earlier? Not entirely clear from the reference CGH studies on line 203.
Figure 3a: samples BC6 and BC10 have the same color code. Usage of unique colours for each sample necessary.
The inference of genomic variation from mRNA data is interesting and informative. The authors should discuss clearly that the variation found here reflects aberrations present in the mRNA and that additional genomic variation might underlie the different cell populations and thereby explaining additional relationships.
Low number of OS cells in ondroblastic OS lesions and the lung metastatic lesions --> could this be due to lack of capturing via single cell mRNA seq? Low sample quality? The authors should discuss this.
Improve readability of legends in suppl. Fig 8.
Suppl. Fig 10d: samples BC11, BC20 and BC22 do not contain mature OC cells- please state why this might be the case.
Suppl Fig 11a/ 14b – proper scale bars necessary.
Suppl Fig 11b – higher magnification necessary, cells are difficult to visualize, scale bar necessary.
Suppl. Fig 15: distinguish between Osteoblast and proliferative osteoblast cells. Overall this figure is helpful but could be slightly re-organized to improve its function to provide an overview.
In supplementary table 1, the cell numbers per patient are listed- the myoblast cluster from fig 1 is basically only comprised of cells from sample BC17- if this cell type is only present in this sample, it cannot really be used as a general subtype across all samples analysed!! This needs to be explained and corrected. In BC5 and BC22, there are 2 myoblast cells found in each sample. They are absent otherwise. This cell type cannot be used to compare all samples. Why is this cell type not present on the majority of the samples? This might be due to under-sampling, and introduction of sampling bias during sample preparation.

While the sample size has not been increased, so that some of the original concerns regarding comparability of data sets from patients remain, the authors have removed statements (e.g. regarding the absence of an expected stem cell population) as well as provided in – depth explanations for the various different computational analyses performed. The authors nicely decipher the various different cell types present, making this manuscript a relevant contribution with regards to classifying this type of tissue. However, lack the cellular throughput and varying gene capturing depth (ca. 450 – 4500 genes) may negatively impact the generation of a refined cell atlas resolving the majority of cell subtypes. Cluster analysis and cell type annotation use main/ canonical markers. More consistent gene capturing across all samples (e.g. >2000genes/ cell) may provide a more refined cell atlas.

The lack of control/ untreated tissue and therefore the missing analysis of chemotherapy- induced transcriptional and genomic alterations is a weakness. Especially since the authors claim high clinical relevance for their findings. While the cell type, trajectory and inferred clonotype analyses are interesting (cell type annotations are limited by the cell number and varying gene capturing), the demonstration of clinically relevance could be stronger.

The following are our point by point responses to the comments of the reviewers.

REVIEWER COMMENTS

Reviewer #1 (Remarks to the Author):

The authors have generally answered and addressed the questions posed in the first review. I have some minor edits and clarifications to suggest.

INTRODUCTION

2_1Q1

Page 4:

They quote an Overall survival rate 25%. That is really not correct. It is only correct for metastatic OSA, not for all osteosarcomas and certainly not for localized osteosarcomas. They should correct this statement. Survival for all osteosarcomas (unselected) is above 50%.

Also, Radiation treatment is not part of the standard or optimal treatment plan for osteosarcoma, which is treated with surgery and chemotherapy. Radiation is only used when surgery is absolutely impossible or for palliation. This should be corrected.

A1

Response: We thank the reviewer for the valuable comments. We agree with the reviewer that the 5-year overall survival is less than 25% for osteosarcoma patients with metastatic osteosarcoma and about 60-68% for all patients [*J Clin Oncol 2002; 20(3): 776–790; Eur J Surg Oncol 2015; 41(3): 407–412.; Cancer. 2006 Mar 1;106(5):1154-61*], and that radiation is recommended for the treatment of patients with unresectable tumors, who had surgery but show positive margins, or as palliative

intervention, according to the National Comprehensive Cancer Network (NCCN) guidelines (version 1.2020; see below). In all cases that we collected, the patients were all treated with chemotherapy plus surgical therapy, but no radiation therapy. We have further revised these statements in the revised version of the manuscript (Line 70).

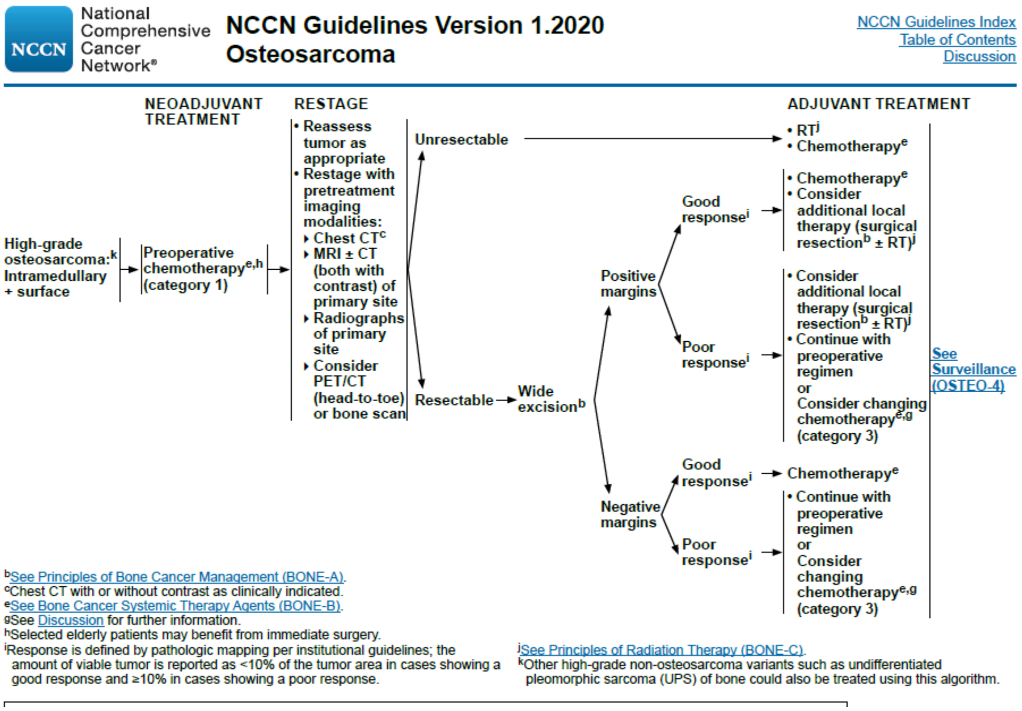

Letter Figure 1. The NCCN guidelines (V1.2020) for the treatment of osteosarcoma. Radiation treatment is recommended for patients with unresectable tumors and those with poor responses to chemotherapy.

RESULTS

2_1Q2:

Page 5:

10, 897 cells: This must be a typo, this count does not agree with counts stated for the same result in other sentences.

A2:

Response: The total single cell number was 100,987 cells in the present study. We apologize for this typo and we have revised this information in the new version of the manuscript (Line 100). On the other hand, in spite that this number referred the total cell number, we described other cell numbers in different categories of cells, ex. different type of cells, and different subcluster of cells.

METHOD

2_1Q3:

Page 27

How many patients received PD-L1 therapy?

The authors provide somewhat confusing information. One sentence states that it was one patient on a prior trial, and another sentence states that all patients received PD-L1 therapy. This should be clarified.

A3:

We apologize for the unclear statement in our manuscript. The fact is that we enrolled one patient (BC17) from a registered clinical trial, for our scRNA-seq study; except this patient, all other cases that we studied were not from this clinical trial. Now we restated it as: "In addition to other patients enrolled for our study, we recruited one patient (BC17) from the clinical trial NCT03676985, in which all patients had undergone neoadjuvant chemotherapy, surgery, and adjuvant chemotherapy, and they all had received anti-PDL-1 therapy for one year until the disease progressed." (Line 628 to 632).

Reviewer #2 (Remarks to the Author):

2_2Q1

ANSWER: 2A1

… and we deleted any discussion on stem cell in this manuscript.

The authors removed the statement about missing stem cells, which might be simply due to lack of under-sampling of cells from metastatic and recurrent OS. The authors also state that these samples are too difficult to obtain to process additional specimen as well as that the fine-needle aspiration of cancer biopsies does not provide cells of sufficient quality and quantity for high throughput analysis.

Different platforms exist which enable processing small samples such as FACS sorting into PCR plates. Furthermore, confirming cell type annotations via an alternative method (e.g. plate-based Smart-seq) would further strengthen the methodology underlying this manuscript and might provide an even more refined cell atlas of a previously understudied tissue by single cell transcriptomics.

Also, if the authors are concerned about sample quality upon biopsy, profiling nuclear RNA has proven to be very informative and to be less affected by sample isolation compared to cells.

2_2A1:

Response: We thank the reviewer for these valuable comments, which allow us to re-consider our experiment design, and any additional experiment that may be added.

The plate-based SMART-Seq method provides deeper information about the transcriptomic profiling of single cells, which includes alternative splicing and possibly single nucleotide mutation message for transcripts besides difference of expression, and may provide more refined cell atlas of osteosarcoma. Compared with the droplet scRNA-seq method, such as 10× Genomics technology, the plate-based SMART-seq method is usually of much lower throughput and is associated with much higher costs. The current study (this manuscript) aimed to depict the cellular heterogeneity atlas in osteosarcoma tissues, therefore we must use a much higher throughput method on a large number of cells, with an affordable cost.

Nevertheless, SMART-Seq method is particular useful for a more intensive study on a much more focused cell type (types), for different purpose, such as transcript mutation and splicing analysis in the regulation of the OS. In the near future when there is a chance, we would like to provide deeper transcriptomics profiling of the FACS sorted osteosarcoma tumor cells, immune cells, or stromal cells etc., with SMART-seq in together with other high throughput scRNA-seq methods, following the reviewer's valuable suggestions.

We also thank the reviewer for the valuable suggestions of single cell nuclear RNA sequencing (snRNA-seq) method, which may be helpful to overcome the cell damage caused during the sample preparation protocol. This approach may represent another helpful platform to depict the transcriptomic landscape of osteosarcoma. We expect to setup the snRNA-seq platform in our lab, which could provide comparable transcriptomics profiling data of osteosarcoma in the near future when only partially damaged samples are available while cell nuclei are intact.

2_2Q2

ANSWER: 2A2

The authors state the lack of untreated controls for each subgroup which makes it impossible to infer treatment induced transcriptional alterations. Instead, if no control tissue is available at their institution nor from an international biobank, they could use the primary OB cells and state that they are used as an approximation due to the lack of the proper control.

The authors should provide evidence for transcriptional signature changes upon treatment as this would add significant value to the manuscript.

2_2A2:

We agree with the reviewer that systematically evaluated the transcriptomics change upon the treatment may provide deep insight into the cellular responses of osteosarcoma cells to the systematic chemotherapy in clinic. We tried to gain the untreated osteosarcoma tissues for each patient; however, the neoadjuvant chemotherapy is a standard treatment for OS and the biopsy before any treatment would possibly promote the malignant cell into circulation, which would increase the risk of metastasis. With ethical concern, we are obligated to minimize the risks in clinic practice and we could only insert a biopsy needle to grab a tiny bit of tissue for pathological diagnosis, in which however the cell number and quality (when dissociated) are not sufficient for a high throughput scRNA-seq analysis.

We also searched the GEO database for transcriptomics data of osteoblasts and chondrocytes, but we did not find any scRNA-seq dataset of human available. In deed we found RNA-seq data on human normal bone biopsies (GSE accession number: GSE141595), normal osteoblast cell lines (GSE89179), osteosarcoma cell lines (GSE118488 comprising 13 cell lines), and osteosarcoma tissues (GSE140131 and GSE124768); however, they were performed with the conventional bulk RNA-seq methods on heterogenous cell types, or on cell lines. The current study was performed

using the scRNA-seq method based on the 10× Genomics platform, which possesses lower coverage of the transcriptomics for a large number of single cells. Because these datasets were different in nature, and were with too many variables, the result would be very likely biased or misleading if we compared the RNA-seq data from the scRNA-seq and the conventional bulk RNA-seq with different natures of samples.

Although drug resistance is not the purpose in this manuscript, we are interested in the topic. In the future, when we have chance we will study the cellular and molecular responses to chemotherapy, radiation, or the immunotherapy using organoid models, patient-derived-xenograft (PDX) models, and other animal models based on the scRNA-seq methods as pointed out by the reviewer.

2_2Q3

ANSWER: 2A3

The authors provide the demanded answers in their reply as well as in the results section. The t-SNE plots of individual samples show the different cell types which are present in all samples. However, the UMI counts are particularly low for BC3 and BC2 and indicate that mRNA capturing/ amplification did not work as well for those two samples. Where these samples processed differently? Why did these two samples generate fewer UMIs and gene counts compared to the other samples?

2_2A3:

Response: We agree with the reviewer. In our study, the UMI counts of BC2 and BC3 are relatively lower than those of other samples, which could be due to the relatively lower sequencing depth in our initial experiments. We followed the general recommendation of the 10×Genomics platform for BC2 and BC3, and because the UMIs were low, we sequenced it at a higher depth and obtained better sequencing results for the resting 9 samples.

We agree with the reviewer that caution should be taken when assessing these two samples. In our bioinformatics analysis pipeline, we have performed several steps to avoid the bias that may derive from BC2 and BC3 samples. First, in the integration step, we applied the Harmony algorithm to account for the batch effect. As shown in Supplementary Figure 3 (the sample stratified t-SNE plot of the cellular clusters) and Supplementary Figure 4 (the sample stratified UMAP plot of the cellular clusters), we noticed a good concordance of the cellular components for each patient, suggesting that the samples BC2 and BC3 have sufficient data in the integrated cellular annotation. Second, the gene expression counts have been normalized and scaled during the t-SNE, UMAP, and the trajectory analysis (with Monocle 2) in the scRNA-seq analysis pipelines, which reduced the bias of sequencing depth of the samples. These results indicated that the relatively lower UMI of BC2 and BC3 had minimal influence on the conclusions in our study.

2_2Q4

ANSWER: 2A4

Good. The authors have further analysed this cluster and provided a definition for 10 subtypes within the myeloid cluster.

2_2A4:

Response: We thank the reviewer for the positive comment.

2_2Q5

ANSWER: 2A5

The authors address the comments and re-analyse the data set for GO term enrichment. Now they show genes which distinctly mark the different clusters (2c) as well as distinct GO terms which show a distinct, cell type-specific pattern (2g). Unfortunately, in Fig 2d, the alphabetical ordering of GO terms makes it difficult to spot overall changes between the cell types. If possible, one could re-order the GO terms in 2c and 2g- similar to Fig. 2c.

2_2A5:

Response: Thanks to the reviewer's valuable suggestions. We have now performed the row clustering analysis in Figure 2d and Figure 2g to make the figure clearer.

2_2Q6

ANSWER: 2A6

The authors addressed the comment by providing detailed description on how Seurat clustering and monocle 2 trajectory inference were performed.

2_2A6:

Response: We appreciate the reviewer for the comment.

2_2Q7

ANSWER: 2A7

The authors did not test trajectory building using another algorithm, as suggested, and focused on monolce2. Their explanation is, that monocle2 is one of the most widely

used software with a good track record. They reference Saelens 2019, which concludes that the choice of trajectory tool is dependent on the data set dimensions and trajectory topology. They state themselves that trajectory tool performance may depend on It would be interesting to see, whether another trajectory inference method would provide a similar picture of developmental trajectories in this particular case. Since central conclusions of this study are based on trajectory analyses, a scientific reasoning for the chosen method would appear natural.

2_2A7:

Response: We understand that the reviewer showed concerns regarding the trajectory analysis method used in our study. Up to date, multiple analysis tools have been developed to infer the cellular trajectory. As pointed by Saelens et al. [*Nature Biotechnology 37, 547–554(2019)*], the choice of the analysis method should depend mainly on the dataset dimensions and trajectory topology. In the current study, we applied the Monocle 2 algorithm to infer the cellular trajectory during osteoclast maturation and the transdifferentiation of malignant chondroblasts into osteoblast cells in osteosarcoma tissues. As noted, Monocle 2 is one of the most widely used software with a good track record. It fits the simple linear (such as the osteoclast maturation in our study), bifurcation (such as the chondroblast transdifferentiation in patients BC20 and BC22), and the multifurcation trajectory types [*Nature Biotechnology 37, 547–554(2019)*]. Nevertheless, following the reviewer's suggestion, we re-analyzed the trajectory data with other tools to check the robustness of the results inferred by Monocle 2.

For osteoclast maturation, we noticed a linear trajectory topology using Monocle 2, which was confirmed by the SCORPIUS algorithm (See Letter Figure 2 below). The SCORPIUS algorithm is an unsupervised approach for inferring linear developmental chronologies from single-cell RNA sequencing data [*Preprint at bioRxiv https://doi.org/10.1101/079509 (2016); Nature Immunology 16 (7): 718–26*]. With

SCROPIUS, we validated the linear transition of osteoclast from the OC progenitor cells and OC immature cells into mature osteoclast in osteosarcoma tissues (Letter Figure 2A). Along the pseudotime, the top differentially expressed genes are presented in Letter Figure 2B (the top 50 genes are shown). Among them, the genes related to myeloid progenitor cells, including CD74, CD14, HLA-DPB1, and HLA-DPA1, were significantly downregulated along the pseudotime, while genes related to mature osteoclast and osteoclast differentiation, such as ACP5, CTSK, CTSB, and SPP1, among other, were significantly upregulated. Furthermore, we also noticed that cell proliferating markers, including HMGB1, TYMS, and MKI67, were significantly downregulated along the trajectory. These results were in agreement with our Monocle 2 inferred results (Figure 4 in the main manuscript).

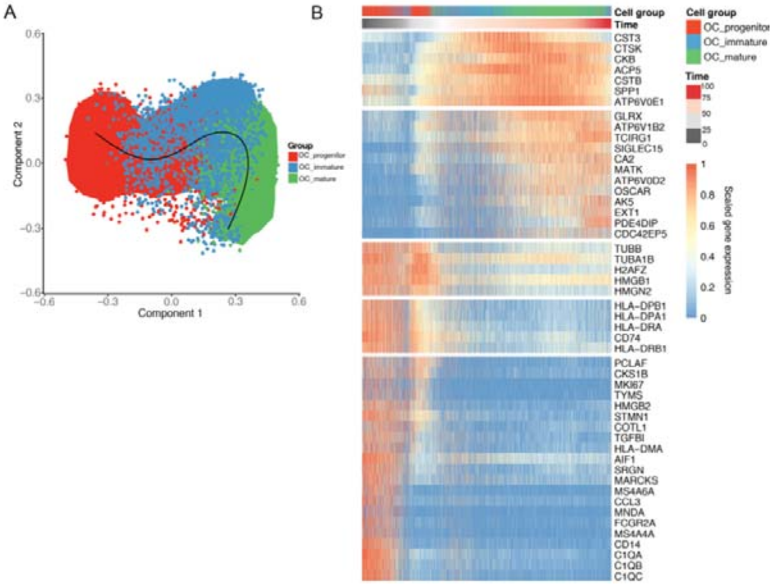

Letter Figure 2. Analysis of single osteoclasts during the cellular differentiation by the SCORPIUS algorithm. (A) Trajectory plot. (B) Expression profile of the top 50 differently expressed genes along the pseudotime trajectory of osteoclast maturation. These genes were grouped into five clusters based on their expression pattern.

For the cellular transdifferentiation of malignant chondroblasts, we noticed the bifurcation trajectory in patients BC20 and BC22, as suggested by the Monocle 2 algorithm. Using the slingshot algorithm [*BMC Genomics. 2018 Jun 19;19(1):477*], another popular trajectory analysis tool that fits the bifurcation trajectory, we

reanalyzed the cellular trajectory of chondroblast osteosarcoma cells (Letter Figure 3 at below). For each cell fate branch, we also determined the genes that significantly changed along the pseudotime using the general additive model (GAM), as suggested by the slingshot guidelines. The 100 most significantly expressed genes are shown (Letter Figure 3B and 3C). In BC20, we also noticed a bifurcation trajectory of the tumor cells, with one branch indicating a transdifferentiation into osteoblastic cells from chondroblastic cells (cell fate 1), while the other branch consisted of chondroblastic cells (cell fate 2). In cell fate 1 branch, we noticed that the top differentially expressed genes were biomarkers of chondroblastic cells, including COL9A1, ACAN, and COL11A1 (Letter Figure 3B, left panel), and an increment on osteoblastic cell biomarkers including COL1A1, SPP1, COL3A1 *etc.* (data not shown) along with the trajectory pseudotime. In the cell fate 2 branch, genes related to osteosarcoma growth and progression, such as PLOD1, MMP2, and STMN1 (Letter Figure 3B, right panel), were significantly upregulated along with the cellular trajectory, suggesting the malignant progression of the chondroblastic cells within osteosarcoma tissues. For BC22 (Letter Figure 3A, right panel), we noticed a bifurcation trajectory of the chondroblastic cells into two distinct osteoblastic cells, which consisted of osteoblasts 1, 2, 3, and 4 (cell fate 1; Letter Figure 3C), and the osteoblasts 5 and 6 (cell fate 2; Letter Figure 3C). Along the pseudotime trajectory, we noticed the downregulation of chondroblastic biomarker genes including COL3A1, SOX9, COL9A1, WWP2, and SNORC, in both branches, and an increased expression of obsteoblast biomarkers including COL1A1, COL1A2, COL3A1, and PTN. These results were in agreement with the Monocle 2 trajectory analysis (Supplementary Figure 9 in the main manuscript). In our slingshot analysis, we determined the genes that were differentially expressed along the pseudotime trajectory in either branch of BC20 and BC22 (Letter Figure 3B and 3C); however, the differences in expression of these genes between the branches were not determined with slingshot method. Monocle 2 uses the BEAM (branched expression analysis modeling) algorithm to identify those genes with branch-dependent expression patterns, which can help identify the mechanism by which the fate decision is made.

Taken together, we prefer to present the trajectory analysis results obtained by Monocle 2 for osteoclast maturation and chondroblast transdifferentiation analysis in our main manuscript. We have provided the bioinformatics scripts of slingshot and SCORPIUS on github (https://github.com/ChenPeizhan/osteosarcoma.git) together with the other scripts generated in the current study for reference. We hope this will meet the reviewer's requirements.

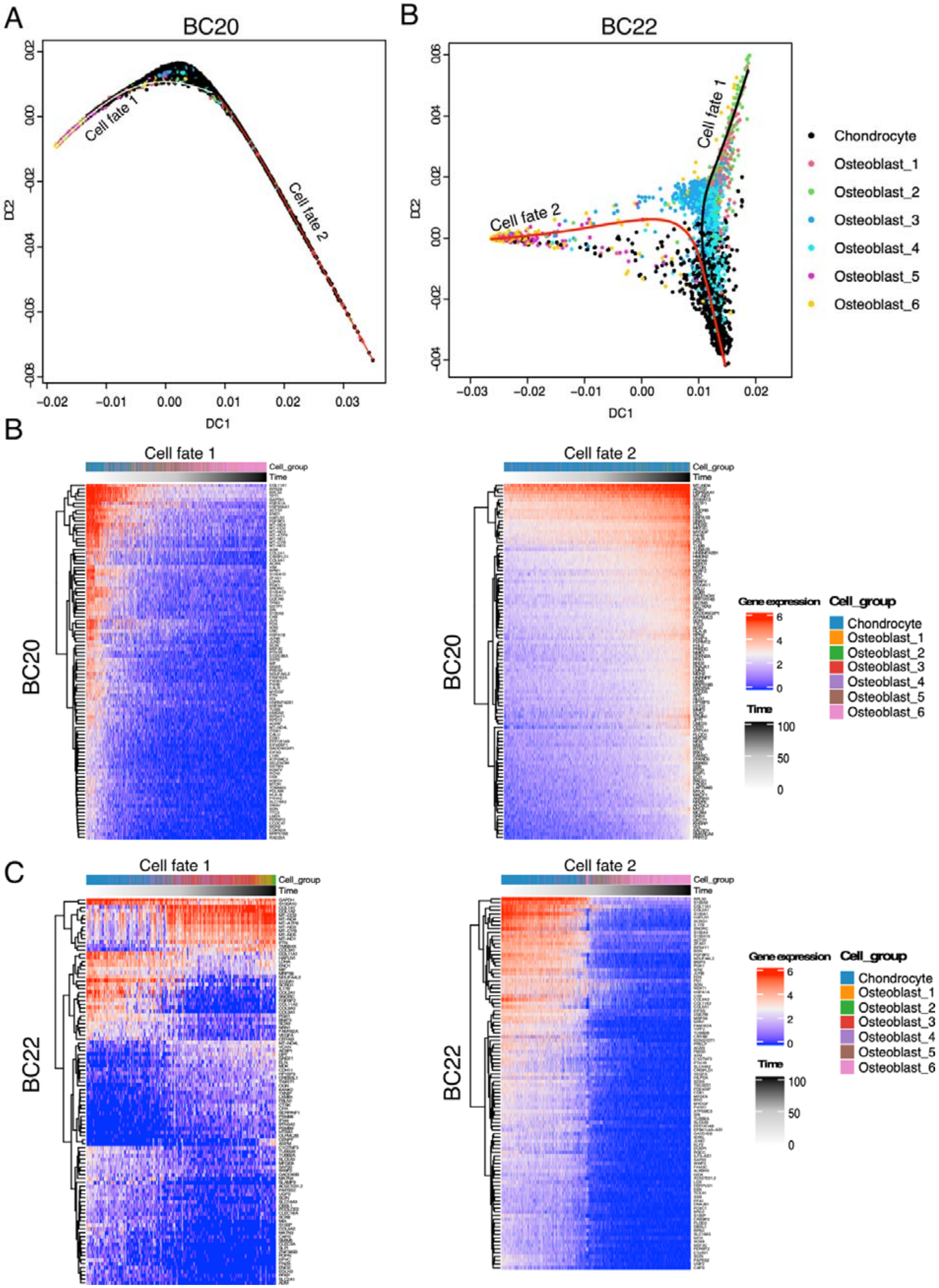
A
BC20
Cell fate 1
Cell fate 2
DC1
DC2
B
BC22
Chondrocyte
Osteoblast_1
Osteoblast_2
Osteoblast_3
Osteoblast_4
Osteoblast_5
Osteoblast_6
B
Cell fate 1
Cell fate 2
Cell_group
Time
BC20
Gene expression
C
BC22

Letter Figure 3. Analysis of the malignant cells in BC20 and BC22 patients by the slingshot algorithm. (A) Cellular trajectory. The dot color indicates the cellular subgroup identified by the t-SNE analysis. (B) Heatmap of the top 100 genes that were differentially expressed along the pseudotime in each cell fate branch in BC20. (C) Heatmap of the top 100 genes that were differentially expressed along the pseudotime in each cell fate branch in BC22.

2_2Q8

ANSWER: 2A8

The genes which are strongest down regulated on Fig 4f are not the genes stated by the authors in the result section. They should comment on those genes (e.g. DBP, ID3,HES4, GLMP) and their biological relevance for the differentiation processes studied here. In the text, the authors state the following genes to be downregulated HMGB1, HMGB2, MEF2C, CREM and LITAF but it is unclear from the text to which figure they refer to. Gene LITAF for example is not in Fig 4f. Please comment and correct.

2_2A8:

Response: We apologize for the unclear statement in our manuscript. We have now added the relevant figures along with the supporting text in the revised version of the manuscript to indicated the genes significantly changed along with the osteoclast maturation including HMGB1 (line 33 in gene cluster 2 of Figure 4f), HMGB2 (line 14 in gene cluster 2 of Figure 4f), MEF2C (line 59 in gene cluster 2 of Figure 4f), CREM (line 27 in gene cluster 2 of Figure 4f) and LITAF (Figure 4f; Line 751). LITAF is ranked as the second to last of the cluster 2 in Figure 4f, which showed significantly downregulated genes along the pseudotime of the trajectory.

We have stated the genes that showed strongest down- or up-regulation in our revised manuscript and their molecular relevance to the osteoclast differentiation. The reviewer suggests to comment the molecular mechanisms of those significantly downregulated

genes such as DBP, ID3, HES4, and GLMP in the osteoclast differentiation. Some of them have been reported to be associated with the osteoclast differentiation, but others have not been associated with this process. For example, ID1 and ID3 (inhibitors of DNA binding 1 and 3, respectively) were reported to be negative regulators of osteoclast differentiation [*Life Sci, 2015 Dec 15;143:1-7*], whereas DBP could be converted into a potent macrophage-activating factor DBP-MAF, which may promote osteoclast activation and bone resorption [*Biochem Biophys Res Commun, 1998 Aug 28;249(3):668-71*]. However, to date, the roles of HES4 and GLMP in the osteoclast maturation have not been reported. Basing on our trajectory analysis, multiple novel transcription factors whose expression was significantly changed along the pseudotime were identified, which may regulate the osteoclast maturation in the osteosarcoma tumor microenvironment. These genes could serve as potential therapeutic targets for osteosarcoma treatment and provide deeper insights into the osteoclast maturation process within osteosarcoma tissues. However, more studies are warranted to elucidate their underlying mechanisms and contribution for osteosarcoma progression. We have now revised these in our manuscript according to the reviewer's suggestions (Line 287 to 292).

ANSWER: 2A9

OK.

ANSWER: 2A10

OK.

2_2Q9

ANSWER: 2A11

In Fig 7f, graphs U2OS:BC16 and 143B:BC16 are exactly identical. Please explain. The authors need to clearly state in the results, that the two patients were selected based

on high TIGT expression in CD3+ T cells. This is relevant to follow the rationale of testing for T cell cytotoxicity in their cellular model.

Given that suitable samples are sparse, the explanation for using these two patients is relevant. However, it would be crucial to see patients' cells performance in this assay with low TIGIT expression- based on the conclusion of the authors, the observed cytotoxicity should be less. This is a logic and necessary experimental confirmation of their hypothesis.

2_2A9:

Response: We apologize for the mistakes in the figure preparation of the right two panels on Figure 7, and we have now revised it based on the original data. As the reviewer pointed out, we only performed cellular cytotoxicity analysis based on the patients BC3 and BC16, who had relatively high $TIGIT^+$ $CD3^+$ T cells infiltration, to evaluate the impact of TIGIT blockage on the T cell cytotoxicity on osteosarcoma cells. Following the reviewer's suggestion, we have evaluated the effect of TIGIT blockage on T cell-mediated cytotoxicity in patients (BC5 and BC6) with low tumor-infiltrating $TIGIT^+$ $CD3^+$ T cells (Figure4). In such cases, only margin effect of T cell-mediated cytotoxicity was observed, suggesting that TIGIT blockage may augment the T cell-mediated cytotoxicity in osteosarcoma patients with relatively higher TIGIT-expressing T cells (Figure 4). We have now added these results in our manuscript as Figure 7f (line 453 to 460).

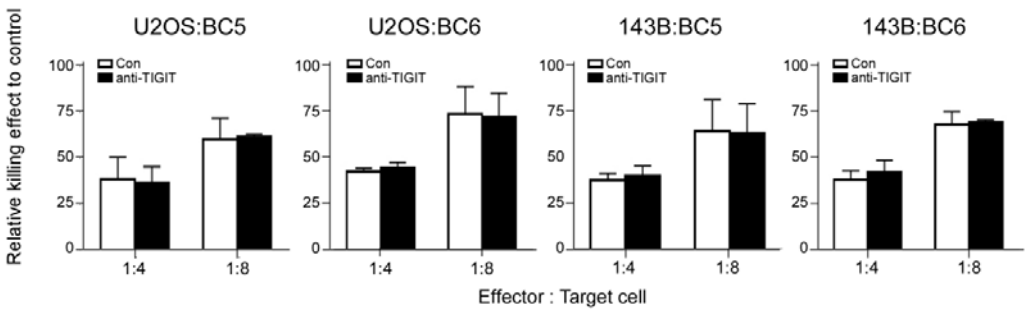

Letter Figure 4. The T cell mediated cytotoxicity activities of CD3+T cells from peripheral blood cells in patients with low TIGIT+ CD3+T cells infiltration on osteosarcoma cell lines U2OS and 143B with TIGIT blockage.

2_2Q10

ANSWER: 2A12

See comment above to substantiate the conclusion that targeting TIGIT-high T-reg cells may present a novel therapeutic approach to OS, the authors should at least show a milder effect in their cellular assay in patient samples with lower fraction of TIGIT-high T-reg cells.

2_2A10:

Response: The reviewer showed concerns about the T cell-mediated cellular cytotoxicity activities upon TIGIT blockage in osteosarcoma patients. As we responded in 2A9, we performed cellular cytotoxicity analysis to evaluate the impact of TIGIT blockage on T cell cytotoxicity on osteosarcoma cells based on samples collected from the patients BC3 and BC16, who had high tumor-infiltrating $TIGIT^{+}CD3^{+}$ T cells infiltration. Significantly enhanced T cell-mediated cytotoxicity was noticed in these two patients. Following the reviewer's suggestion, we also evaluated the impact of TIGIT blockage on T cell cytotoxicity in patients BC5 and BC6 with low tumor-infiltrating $TIGIT^{+}CD3^{+}$ T cells infiltration (See Letter Figure 4 above). In this case, only margin effect cellular cytotoxicity was observed, which suggests that TIGIT

blockage may augment the T cell-mediated cytotoxicity in osteosarcoma patients with high tumor-infiltrating $TIGIT^+$ $CD3^+$ T cells.

We have now included this new information in our manuscript (Line 628 to 642). Meanwhile we acknowledged that a good space is available to extend this study, and the new study requires additional subjects and a systematic design.

ANSWER: 2A13

OK.

ANSWER: 2A14

OK.

2_2Q11

OTHER COMMENTS:

Typo line 100: total number of cells is too low, does not add with the following cell counts

2_2A11

Response: The total cell number of our current study was 100,987. We apologize for the typing mistakes and we have further revised the manuscript (Line 100).

2_2Q12

Line 150: typo --> BC17 instead of BC107

2_2A12:

Response: We apologize for the typing mistakes and we have further revised the manuscript accordingly.

2_2Q13

Ordering of Fig 2d to be changed --> such as in "stair-case" like plot Fig 2c

2_2A13:

Response: Following the reviewer's suggestions, we applied the row clustering analysis presented for the GSEA results to the hallmark gene sets in Figure 2d and Figure 2g.

2_2Q14

Chromosomal lesions: 1p gain, 1q gain, 2q gain, 17q gain, 201 and 21q gain --> were they observed in OS earlier? Not entirely clear from the reference CGH studies on line 203.

2_2A14:

Response: In the current study, we used the inferCNV algorithm (inferCNV of the Trinity CTAT Project (https://github.com/broadinstitute/inferCNV) to infer the copy number variation (CNV) of the 11 osteosarcoma patients (Figure 3 and Supplementary Figure 8). With this approach we identified several frequent canonical CNV events in these patients, including the 1p gain, 1q gain, 2q gain, 17q gain, 19p gain, and 21q gain, as pointed out by the reviewer. To date, there are several whole-genome sequencing (WGS) and comparative genomic hybridization (CGH) studies [*Genes Chromosomes Cancer. 2005 Feb;42(2):158-63 (ref19); Mol Cancer Res. 2008 Jun;6(6):937-46; Cell Rep. 2014 April 10; 7(1): 104–112; Cancer Discov. 2019 Jan;9(1):46-63*] have evaluated the CNVs in the osteosarcoma tissues. In the study performed by Atiye et al.

[*Genes Chromosomes Cancer. 2005 Feb;42(2):158-63 (ref19)*] reported the 1p34-36, 1q21, 17q25, 8q24, 19q13, and 21q22 gain in osteosarcoma tissues, which are partially overlapped with our current study.

As pointed out by Sayles et al. [*Cancer Discov. 2019 Jan;9(1):46-63*], osteosarcoma is characterized by significant somatic copy-number alteration (SCNA) and structural variation (SV) with few recurrent point mutations in protein-coding genes, with the exception of the tumor suppressors RB1 and TP53. The SCNAs and structural rearrangements are highly heterogeneous across osteosarcoma cases, which were further confirmed in our inferCNV analysis by identifying distinct canonical CNVs in individual patients. Based on the inferCNV data, we also performed clonality analysis of single tumor cells from individual patients, revealing high intratumoral heterogeneity of the osteosarcoma patients. We have further discussed these results and added more references to support this in our revised manuscript (Line 222-228 and line 504-515).

2_2Q15

Figure 3a: samples BC6 and BC10 have the same color code. Usage of unique colours for each sample necessary.

2_2A15:

Response: The reviewer's suggestion is highly appreciated! Following the reviewer's suggestion, we have revised the barcode indicating the sample ID in the Figure 3a.

2_2Q16

The inference of genomic variation from mRNA data is interesting and informative. The authors should discuss clearly that the variation found here reflects aberrations present in the mRNA and that additional genomic variation might underlie the different cell populations and thereby explaining additional relationships.

2_2A16:

Response: We agree with the reviewer that as the CNV events identified in the current study were derived from the single cell mRNA expression data, which however actually reflected the somatic copy-number alteration (SCNA) at the genomic level.

Indeed, a significant part of the CNVs identified from the inferCNV were reported in previous whole-genome sequencing (WGS) and comparative genomic hybridization (CGH) studies [*Genes Chromosomes Cancer. 2005 Feb;42(2):158-63 (ref19); Mol Cancer Res. 2008 Jun;6(6):937-46; Cell Rep. 2014 April 10; 7(1): 104–112; Cancer Discov. 2019 Jan;9(1):46-63*]. This inferCNV result (when a genomic region is monoploid or delected or duplicated) is not based on one individual mRNA, but based on a series of mRNAs coded by a series of corresponding genes located in a continuous genomic region. Only when a genomic region is alternated in CNV, the multiple mRNAs (usually are with different functions, and associated to different pathways or GOs) coded by the region will consistently changed accordingly. Therefore the CNV calling is not determinated by the changes of some of the mRNAs in this regions, but all mRNAs in the region. Previous studies have validated the CNV data inferred from the scRNA-seq with the single cell DNA sequencing (scDNA-seq) method, and a good concordance was reported between the two methods [such as: *Nat Commun. 2020 Jan 24;11(1):496*].

Based on the inferCNV results, we analyzed the clonality profile of single osteosarcoma cells (Figure 3 and Supplementary Figure 8), which was found to be significantly heterogeneous between cells with certain patterns across the cells. In

addition, the clonality analysis results revealed the previously unappreciated complexity of both canonical and non-canonical CNVs in osteosarcoma (Figure 3a and Figure 3b). As expected, the canonical CNVs dominated the chromosomal landscape. Nevertheless, there are still multiple subclonal canonical and noncanonical CNVs across osteosarcoma patients, which may underlie the subclonal cell populations in the tumor cellular evolution (Figure 3a and 3b). We hope these will meet the reviewer's requirements (Line 222-228).

2_2Q17

Low number of OS cells in ondroblastic OS lesions and the lung metastatic lesions --> could this be due to lack of capturing via single cell mRNA seq? Low sample quality? The authors should discuss this.

2_2A17:

Response: We agree with the reviewer that relatively few osteoblast tumor cells were identified in the chondroblastic OS lesions and lung metastasis lesions (Figure 1e and Supplementary Figure 3 and Figure 4). As the study revealed in the manuscript that the malignant osteoblasts in the chondroblast osteosarcoma were derived from the malignant chondroblast, there were both malignant osteoblastic and chondroblastic cells in the chondroblastic lesions (BC20 and BC22). Therefore, it is not unreasonable that lower OS cell numbers were detected in these two patients. We do not think it is due to any technical bias, because the 10x sequencing system is well validated worldwide in the field, neither due to low quality of sample because we have performed QC test and we did not find any sign showing that it is a bad sample.

On the other hand, in the lung metastasis lesion of the BC17 patient, we noticed that stromal fibroblast was enriched to a relatively higher number in the lung metastasis lesion than other samples.

2_2Q18

Improve readability of legends in suppl. Fig 8.

2_2A18:

Response: We apologize for the unclear statement on the figure legends and we have further revised it for Supplementary Figure 8. The scaled CNV scores were annotated by hierarchical clustering to indicate the 22 chromosomes according to the inferCNV analysis. The bar shows the scaled CNV score of the individual gene region on single cells. We have now added the information in the figure legend according to reviewer's suggestion (Supplementary Figure 8).

2_2Q19

Suppl. Fig 10d: samples BC11, BC20 and BC22 do not contain mature OC cells- please state why this might be the case.

2_2A19:

Response: We agree with the reviewer that no mature OC (osteoclast) was detected in BC11, BC20, and BC22 in the osteosarcoma tissues (Supplementary Figure 10) as annotated in the scRNA-seq analysis. Although OC progenitor and immature cells were observed in these two patients, the cellular proportion of osteoclast was relatively low in these patients (Figure 1e). BC11 was a recurrent osteoblastic osteosarcoma patient, whereas BC20 (recurrent tumor) and BC22 (primary tumor) were both chondroblastic osteosarcoma. We have reason to believe that this information reflect that the heterogeneous tumor microenvironment modulates the cellular differentiation of the

osteoclast from myeloid cells, in particular in chondroblast osteosarcoma (BC20 and BC22).

It is well known that osteoblast cells can stimulate the osteoclast differentiation and activation through the RANKL/RANK signaling pathway [*Endocrinology. 2001 Dec;142(12):5050-5*], suggesting that the osteoclast maturation can be stimulated by the RANKL expressed by malignant osteoblast cells in osteosarcoma tissue. Compared to osteoblastic cells, the chondroblast cells were found to have lower RANKL expression, and few osteoclasts were noticed in the chondroblastic-riched resting zone and hypertrophic zone compared to the osteoblastic cells riched calcification zone in human growth plate [*BMC Dev Biol. 2014 Aug 28;14:36*]. Thus, the tumor microenvironment of chondroblast osteosarcoma may hinder the OC mature by the chondroblasts in patients BC20 and BC22.

Furthermore, chemotherapeutics may also influence the osteoclast maturation in osteosarcoma tissues. For example, the chemotherapeutic agent gemcitabine used in the treatment of osteosarcoma patients was found to reduce the number of myeloid-derived osteoclast progenitor cells *[J Immunol. 2007;179:977–983; Cancer Res 2013 Jan 15;73(2):672-82; Cancer Res. 2013 Aug 1; 73(15): 4606–4610]*. In agreement with these reports, three patients in our study (BC10, BC11, and BC17) who received gemcitabine treatment showed relatively low levels of mature osteoclasts. Altogether, it is reasonable to hypothesize that the tumor microenvironment and the chemotherapy treatment may modulate the osteoclast maturation in osteosarcoma tissues; however, further studies are warranted. We have discussed these aspects in our revised version of the manuscript and in hope these will meet the reviewer's requirement (Line 531 to 552).

2_2Q20

Suppl Fig 11a/ 14b – proper scale bars necessary.

2_2A20:

Response: We thank the reviewer for the helpful comment. We have now added the scale bar in Supplementary Figure 11a and Supplementary Figure 14b according with the reviewer's suggestion.

2_2Q21

Suppl Fig 11b – higher magnification necessary, cells are difficult to visualize, scale bar necessary.

2_2A21:

Response: We have included the scale bar in the new Supplementary Figure 11b and also provided an immunofluorescent image of higher magnification (×100 magnification of the optic microscope) in the edited version of the manuscript.

2_2Q22

Suppl. Fig 15: distinguish between Osteoblast and proliferative osteoblast cells. Overall this figure is helpful but could be slightly re-organized to improve its function to provide an overview.

2_2A22:

Response: We thank the reviewer for these helpful comments. We have modified Supplementary Figure 15 in light of the reviewer's suggestion. We hope it now meets the reviewer's expectations.

2_2Q23

In supplementary table 1, the cell numbers per patient are listed- the myoblast cluster from fig 1 is basically only comprised of cells from sample BC17- if this cell type is only present in this sample, it cannot really be used as a general subtype across all samples analysed!! This needs to be explained and corrected. In BC5 and BC22, there are 2 myoblast cells found in each sample. They are absent otherwise. This cell type cannot be used to compare all samples. Why is this cell type not present on the majority of the samples? This might be due to under-sampling, and introduction of sampling bias during sample preparation.

2_2A23

Response: We agree with the reviewer that the myoblast cell number is relatively small in the osteosarcoma tissues, with 108 cells were annotated as the myoblast in BC17 (3.24%), 2 in BC5 and 2 in BC22 (Figure 1e and Supplementary Figure 3 and 4), and no myoblast cell was noticed in the other samples. These cells were annotated as the myoblast as they showed relatively high expression of MYLPF and MYL1 (Cells. 2020 Apr 22;9(4):1045; Elife. 2016 Sep 23;5:e17985.). As we know, the osteosarcoma is a highly heterogeneous disease between patients, which may be developed from different bone sites in human body; the composition and ratio of cell types could be varied between samples as well as patients. As we aimed to depict an intact cellular atlas of the osteosarcoma tissues and we detected these cells in 3 patients (at least one patient BC17, if BC5 and BC22 are not considered), we did not exclude these cells in our bioinformatics pipelines, but faithfully reflected the truth and gave our interpretation.

Reconsidering the reviewer's comments, we suggest that the rarely detected myoblasts may due to heterogeneity of the OS tissues (including metastatic OS), under-sampling,

but less likely any contamination in sampling. Taking all together, the myoblasts may not be regarded as a typical subcluster of cells in OS, but we could not exclude the possibility that myoblasts may be a part of OS cells in some samples. Also, because the ratio of myoblasts is low (it is 3.24% in BC17), any comparison of cell subclusters across sample will not cause a significant distortion with or without counting of myoblasts. Finally, following the reviewer's suggestion, we turned down the statements of myoblast cells in the revised manuscript, and hope it will meet the reviewer's requirements. (Line 132 to 138).

2_2Q24

While the sample size has not been increased, so that some of the original concerns regarding comparability of data sets from patients remain, the authors have removed statements (e.g. regarding the absence of an expected stem cell population) as well as provided in – depth explanations for the various different computational analyses performed. The authors nicely decipher the various different cell types present, making this manuscript a relevant contribution with regards to classifying this type of tissue. However, lack the cellular throughput and varying gene capturing depth (ca. 450 – 4500 genes) may negatively impact the generation of a refined cell atlas resolving the majority of cell subtypes. Cluster analysis and cell type annotation use main/ canonical markers. More consistent gene capturing across all samples (e.g. >2000genes/ cell) may provide a more refined cell atlas.

2_2A24

Response: We agree with the reviewer that the gene coverage derived from the 10× Genomics platform is usually lower, which may restrict our analysis to identify novel cellular clusters and to generate a more refined cellular atlas of osteosarcoma. Indeed in the recent two years this range of gene number (mean detected gene number range from 704 to 4,543 for current study) is well acceptable in cell clustering analysis in many

decent publications. Along with the reduced sequencing cost and the improvement of the single cell capturing methods (such as the SMART-seq method), researchers may perform single cell transcriptomics with deeper coverage of the genes in the near future, which may provide a more refined cell atlas of osteosarcoma or for different purpose if they wish.

2_2Q25

The lack of control/ untreated tissue and therefore the missing analysis of chemotherapy- induced transcriptional and genomic alterations is a weakness. Especially since the authors claim high clinical relevance for their findings. While the cell type, trajectory and inferred clonotype analyses are interesting (cell type annotations are limited by the cell number and varying gene capturing), the demonstration of clinically relevance could be stronger.

2_2A25

Response: Again we thank the reviewer for the suggestive comments. We acknowledged that with untreated tissue and chemotherapy-induced transcriptional and genomic alterations, this study would be even stronger. However, the available data firmly support the current research purpose and conclusion, and the additional mission may be obtained possibly in an independent project, in which we will specifically design it such that the obstacles in sample preparation and clinical ethic requirement are resolved. We would like to perform more studies to evaluate the cellular transcriptomics upon chemotherapy with the organoid models or in patient-derived xenograft (PDX) models in our future research, as we mentioned in response 2_2A2.

Thanks a lot for all the great suggestions to improve our work.

Reviewer #2 (Remarks to the Author):

The authors have generally answered and addressed the questions posed in the previous Review and have improved both text and figures. Below are some minor comments.

2_2A5:

Figures 2 c and 2g are now much clearer and allow the reader to grasp the plots easier.

2_2A7:

The authors nicely demonstrate the robustness of their trajectory analyses by using a alternative approaches. They should state in the results and/or methods section that different trajectory inference methods were used to confirm their results. Good!

2_2A8:

The authors satisfactorily answered the comments.

2_2A9:

The authors corrected the erroneous figure 7f and corrected the graphs U2OS:BC16 and 143B:BC16. They also performed cytotoxicity analysis on TIGIT low expressing samples. No further comments.

2_2Q15
the authors changed the color coding which is good; samples BC20+21 have almost the identical color. If possible, change one of them to more readable colour. Otherwise OK.

2_2Q21
Suppl Fig 11b: are the images labelled correctly? 400 and 630X? or rather 40 and 63X?

2_2Q22
OK.

2_2Q23
It is good that the authors state openly that the myoblast cells are mainly present in BC17 and that they provide possible explanations why this might be the case: heterogeneity and possibly sampling bias. Good.

2_2Q24
OK.

Reviewer #2 (Remarks to the Author):

The authors have generally answered and addressed the questions posed in the previous Review and have improved both text and figures. Below are some minor comments.

2_2A5:

Q. Figures 2 c and 2g are now much clearer and allow the reader to grasp the plots easier.

A. We thank the reviewer for the encouraging comments.

2_2A7:

Q. The authors nicely demonstrate the robustness of their trajectory analyses by using a alternative approaches. They should state in the results and/or methods section that different trajectory inference methods were used to confirm their results. Good!

A. We thank the reviewer for the positive comments. According to editor`s advice we added this part as Supplementary Fig. 10 and Supplementary Fig. 12 in the revised manuscript.

2_2A8:

Q. The authors satisfactorily answered the comments.

A. We thank the reviewer for the positive comments.

2_2A9:

Q. The authors corrected the erroneous figure 7f and corrected the graphs U2OS:BC16 and 143B:BC16. They also performed cytotoxicity analysis on TIGIT low expressing samples. No further comments.

A. We thank the reviewer for the positive comments.

2_2Q15

Q. the authors changed the color coding which is good; samples BC20+21 have almost the identical color. If possible, change one of them to more readable colour. Otherwise OK.

A. We thank the reviewer for the positive comments, and we have further revised the Figure 3 accordingly.

2_2Q21

Q. Suppl Fig 11b: are the images labelled correctly? 400 and 630X? or rather 40 and 63X?
A. We thank the reviewer for pointing out the error. We have checked the data and confirmed the original magnification of the images. The magnification of the images is 400× and 630×, respectively.

2_2Q22
Q. OK.

A. We thank the reviewer for the positive comments.

2_2Q23
Q. It is good that the authors state openly that the myoblast cells are mainly present in BC17 and that they provide possible explanations why this might be the case: heterogeneity and possibly sampling bias. Good.

A. We thank the reviewer for the positive comments, which allow us further clarifying the issue.

2_2Q24
Q. OK.

A. We thank the reviewer for the positive comments.

Here we would like to express our sincerely thanks to the reviewers' comments and suggestions that help us to improve our work.
